# Retrospective Identification of Novel and Legacy Per- and Polyfluoroalkyl Substances in German Archived Fish Livers Using a Combined High-Resolution Mass Spectrometry Approach

**DOI:** 10.1021/acs.est.4c11600

**Published:** 2025-06-20

**Authors:** Silvia Dudášová, Urs Berger, Bettina Seiwert, Thorsten Reemtsma, Oliver J. Lechtenfeld, Qiuguo Fu

**Affiliations:** † Department of Environmental Analytical Chemistry, 28342 Helmholtz Centre for Environmental Research - UFZ, Permoserstraße 15, 04318 Leipzig, Germany; ‡ Institute for Analytical Chemistry, University of Leipzig, Linnéstrasse 3, 04103 Leipzig, Germany

**Keywords:** per- and polyfluoroalkyl substances, FT-ICR MS, LC-QTOF MS, fluorinated ionic liquids, freshwater fish

## Abstract

Per- and polyfluoroalkyl substances (PFAS) are persistent environmental pollutants with many unknown variants, posing potential ecosystem and human health risks. To comprehensively assess PFAS contamination and overcome the challenge of unknown PFAS identification, we combined Fourier transform ion cyclotron resonance mass spectrometry (FT-ICR MS) and liquid chromatography—quadrupole time-of-flight mass spectrometry (LC-QTOF MS) to identify novel and *Legacy* PFAS in archived bream liver samples collected in Germany between 1996 and 2020. By leveraging the ultrahigh resolution and mass accuracy of FT-ICR MS, we generated a mass list for cross-comparison with common precursor ion features from LC-QTOF MS. We identified 78 PFAS, including 68 classified into 12 homologue groups and 10 that did not fit into any homologue series, encompassing perfluoroalkyl acids, fluorotelomer-based substances, pentafluorosulfide perfluoroalkyl sulfonic acids, polyfluoroalkyl sulfinates, polyfluoroalkyl sulfonyl sulfonamides, and other novel compounds including ionic liquids. Spatial and temporal analysis revealed that perfluoroalkyl phosphinic acids (C6/C6, C6/C8) and polyfluoroalkyl sulfonic acids (n = 10) have a widespread presence, whereas tris­(pentafluoroethyl)­trifluorophosphate (FAP) and polyfluoroalkyl sulfonyl sulfonamides (n = 6) are more localized or have only recently emerged in specific regions. These findings underscore the need for continued monitoring to comprehensively understand exposure to PFAS and their long-term environmental impact.

## Graphical abstract

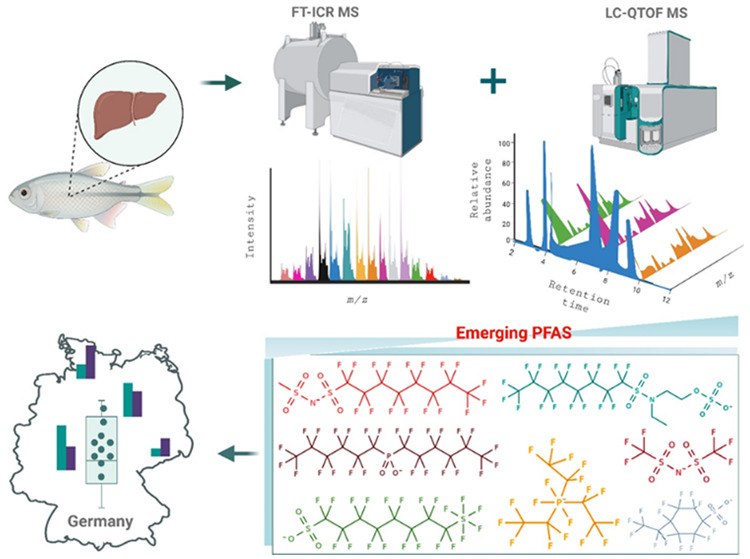

## 1 Introduction

Per- and polyfluoroalkyl substances (PFAS) represent a diverse group of man-made chemicals that have been extensively used in various industries since the 1940s when their production scaled up to meet the demands of modernizing societies.^1,2^ The widespread use of PFAS has led to the global distribution of these chemicals, which are frequently found in water bodies, wildlife, and human populations around the world.^2–5^ Concerns with PFAS include their persistence and association with various health issues, such as cancer, hormone disruption, and immune system effects, prompting a reevaluation of their safety and environmental impact.^6–9^ Their omnipresence and the potential health risks associated with prolonged exposure have spurred scientific and regulatory communities to action.^10,11^

To effectively detect the extent of PFAS contamination, a variety of analytical techniques is typically utilized. Extractable organic fluorine (EOF) and the total oxidizable precursor assay (TOPA) play central roles in determining sum levels of PFAS within samples.^12,13^ EOF is used to measure all extractable fluorinated organic compounds, providing a comprehensive overview, whereas TOPA targets the conversion of PFAS precursors into measurable perfluoroalkyl acids, thus delivering more specific information on PFAS contamination. While useful for detecting PFAS precursors, TOPA has limitations, including incomplete oxidation and the inability to specifically identify individual precursor compounds. Therefore, for structure identification, advanced mass spectrometry techniques are utilized, including ultrahigh and high-resolution mass spectrometry (UHRMS, HRMS), e.g. Fourier transform ion cyclotron resonance (FT-ICR), Orbitrap and quadrupole time-of-flight (QTOF) mass spectrometry.^14–19^

Despite increased research efforts on *Legacy* PFAS, the screening and detection of the broader PFAS class, including less-studied compounds, in environmental samples continue to present substantial challenges. Traditional analytical methodologies mainly rely on target analysis, which is selective and sensitive for the analysis of known PFAS. However, this approach requires prior knowledge of specific chemicals and the availability of standards and misses novel or unexpected PFAS. Recent advances aim to address these limitations by developing suspect and nontargeted analysis techniques that are crucial for providing a comprehensive overview of pollution.^20–25^ However, even with these advancements, the detection of novel PFAS remains a challenging task. The complexity of environmental matrices, the large number of peaks/features from HRMS, and the limited analytical window of MS methods and techniques make comprehensive identification and structure elucidation extremely challenging.^26^

With advanced methods, we can retrospectively analyze samples to check for ongoing PFAS presence in the environment.^27^ Our study performs extensive PFAS screening on archived samples from four German river locations, chosen due to a high unidentified PFAS fraction seen in previous target analysis and TOPA results.^4^ Our goal is to identify both *Legacy* and *Emerging* PFAS using a combined HRMS method. By leveraging the ultrahigh resolution and mass accuracy of FT-ICR MS, we generate a mass list for cross-comparison with precursor ion data from LC-QTOF MS, enabling the identification of potential PFAS candidates. This approach streamlines analysis, reduces noise, and extracts accurate mass and isotopic information. It enables comprehensive screening of environmental samples, identifying both known compounds and potentially novel ones that are not listed in suspect lists or public databases like PubChem. We additionally compared PFAS distribution in bream livers collected from the Rhine river in Koblenz from 1996 to 2020, preselecting those with the highest peak areas to observe changes in their presence over time and across four different locations, offering a deeper understanding of their environmental burden to the aquatic environment.

## 2 Materials and Methods

### 2.1 Samples

The bream liver samples (lat. *Abramis brama*) analyzed in this study were part of the German Environmental Specimen Bank (UPB) monitoring program of the federal German Environment Agency (UBA), previously analyzed in the FLUORBANK project.^28^ The current study analyzed pooled bream liver samples from four locations: Blankenese (river Elbe), Dessau (river Mulde), Prossen (river Elbe), and Koblenz (river Rhine). For the first three locations, samples were collected in 2001 and 2018, while at Koblenz, 9 samples spanning 1996 to 2020 were analyzed to investigate known and unknown PFAS. Each composite sample represented at least 20 individuals per location and was cryomilled to ensure homogeneity. The sampling, pooling, and archiving procedures followed the standard operating procedure of the Environmental Specimen Bank, which outlines standardized methods for bream liver sample collection and processing.^29^

For results on total organofluorine analysis, the TOPA, and quantified data for 66 PFAS, refer to the study by Rupp et al.^4^ Detailed information about sample locations is available in the Supporting Information (SI)_A: Figure S1 and Table S1, respectively.

### 2.2 Chemicals and Standards

SI_A: Tables S2 and S3 provide details about the chemicals and standards. SI_A: 1.2 describes how the standard solutions were prepared.

### 2.3 Sample Preparation

The extraction method is detailed in the study by Rupp et al, 2025.^4^ Briefly, the bream liver sample was wetted with 3 mL of acetonitrile in a polypropylene vial and internal standard solution(s) (50 μL for PAP analysis (10 μg L^–1^ diPAP/50 μg L^–1^ monoPAP) and 25 μL for the other analyses (20 μg L^–1^)) were added. Then, an additional 5 mL of acetonitrile was added, and the sample was vortex-mixed (Heidolph, REAX 2000), ultrasonicated (Bandelin, SONOREX RK 510) for 15 min at 25 °C, and centrifuged (Eppendorf 5804) for 5 min at 2,000 rpm. The supernatant extract was transferred to a polypropylene tube and the extraction was repeated three times in total. The combined extracts were concentrated to 1 mL under nitrogen, then transferred to a 2 mL centrifuge tube with 20 mg of graphitized carbon (ENVI-Carb, Superclean, 120/400 mesh, Supelco, Sigma-Aldrich, Bellefonte, USA) and 50 μL of glacial acetic acid. Following vortex-mixing and centrifugation for 10 min at 10,000 rpm, the supernatant was combined with 500 μL of 4 mM aqueous ammonium acetate and stored at −18 °C. Before analysis, the extracts were filtered through a 0.2 μm RC4 filter (Minisart, PP-housing, Sartorius, Stonehouse, UK) into polypropylene autosampler vials.

### 2.4 UPLC-QTOF MS Method

The HRMS measurements were performed on a quadrupole Time of Flight mass spectrometer (QTOF) (Xevo G2S TOF, Waters, Milford, Germany) in continuum (MS^E^) mode. In this mode, precursor ion spectra were acquired using low collision energy to preserve intact ions, while fragment ion spectra were obtained with ramped collision energy to maximize structural information from fragment ions. Although MS^E^ mode allows for comprehensive fragmentation data, even for low-intensity precursors, it can introduce challenges such as overlapping fragments from coeluting compounds or background noise. However, examining the peak shapes of fragment ions can help establish their relationship with the corresponding precursor ions, mitigating potential misinterpretations. The instrument was operated at a resolution of 20,000 (fwhm), achieving a mass accuracy of 5 ppm. Chromatographic separation was performed using an UPLC system (Acquity, Waters, Eschborn, Germany) equipped with a BEH column (2.1 × 150 mm, 1.7 μm, Waters). An extract volume of 10 μL was injected, and the separation was carried out at a flow rate of 350 μL/min. The instrumental parameters and gradient table are provided in SI_A: Table S6a, S6b.

### 2.5 FT-ICR MS Analysis

Four bream liver samples were measured using direct infusion (DI) into an FT-ICR mass spectrometer (solariX XR, Bruker Daltonics, Billerica, U.S.A.) equipped with a 12 T refrigerated, actively shielded superconducting magnet (Bruker Biospin, Wissembourg, France). Sample extracts were diluted 1:100 in a methanol/water mixture (1:1, v/v) and directly injected into the ESI source at a flow rate of 240 μL/h. Broadband mass spectra acquisition (data size of 4 Megaword) was conducted in negative ionization mode under the following conditions: capillary voltage of 4.2 kV, nebulizer gas pressure of 1.0 bar, dry gas temperature of 250 °C, and dry gas flow rate of 8.0 L/min. We used Q-isolation to segment the mass range (*m*/*z* 150 – 2000) into 11 segments. Mass spectra were internally calibrated using a list of lipids (between *m*/*z* 87 and 1572, n = 473), resulting in mass accuracy <0.5 ppm across the mass range. Mass calibration was performed using DataAnalysis (5.0, Bruker Daltonics). Data from Koblenz (2008) sample was previously published in the study by Dudasova et al. (2024).^30^ The samples from Blankenese (2020), Dessau (2018), and Prossen (2018) were analyzed using a refined segmentation,^31,32^ with adjusted start *m*/*z*, ion accumulation time, transient length, and resolution (SI_A: 4. FT-ICR MS).

### 2.6 Data Processing

The precursor ion LC-QTOF data were initially processed in MZmine3 to create a mass list, which was extracted as. CSV file. A mass list from FT-ICR MS (UHRMS) was obtained from DataAnalysis and served as a reference for identifying masses in the LC-QTOF MS (HRMS). Data from FT-ICR MS and LC-QTOF MS were then compared to obtain a common feature list in a combined HRMS approach by implementing *PFlow*, specifically designed for UHRMS data.^30^ *PFlow* consists of two distinct data processing workflows: the first is designed to generate a suspect list of PFAS using publicly available resources; in this study, we used the CompTox list (SI_B).^33^ The second workflow uses this suspect list to analyze the measurement data for PFAS identification. Masses detected in both HRMS data sets were selected, allowing the detection of PFAS that are not present in existing databases. However, the Prossen sample was excluded from this comparison due to an insufficient number of calibrants in FT-ICR MS analysis, which prevented reliable data calibration. Instead, we used the combined results from the Blankenese, Dessau, and Koblenz samples to identify potential suspect compounds that might also be present in the Prossen sample.

After establishing the list of masses detected in both data sets, we proceeded with compound verification. An initial screening was performed using the suspect list from CompTox.^33^ For those masses that were not found there, we performed a second search in the PubChem database. All conditional filtration steps were conducted in the KNIME Analytics platform.^34^ For the structural confirmation, the mass spectrometric data were analyzed using MassLynx software (Waters).

Further details on the methods and tools used can be found in SI_A: Table S4, Table S5, and Table S7. The stepwise data processing and criteria in each step are described in SI_A: 6 and Figure S3.

### 2.7 Normalization Strategy for Estimating and Comparing the Relative Abundance of *Emerging* PFAS

To evaluate the temporal trends of the identified PFAS, we employed a normalization approach based on internal standards. First, the average peak area of the internal standards was calculated across all years spanning 1996 to 2020 (Koblenz samples). This average served as a baseline to compute a normalization factor for each year, accounting for variability in the internal standard signal (SI_A: Equation S1). Normalized peak areas for both suspect and target compounds were calculated by multiplying the raw peak area by the corresponding normalization factor (SI_A: Equation S2). The inclusion of target compounds in this process provided a reference baseline for comparison, as they represent well-characterized PFAS with similar chemical properties to the suspects. To determine relative abundances, we used the monoisotopic peak intensity from the mass spectrum obtained via LC-QTOF, focusing on the most abundant ion signals for each compound. Following this normalization process, we performed quartile analysis to identify the most dominant compounds across the years. Quartile analysis allowed us to focus on key compounds contributing significantly to the observed trends. For the most dominant compounds and their homologues, individual plots were created to visualize their temporal trends. To ensure comparability, all final data obtained from the normalization process were scaled between 0 and 100% and plotted accordingly (SI_A: Equation S3), allowing for a direct comparison of trends across different compounds and years. Further details on the calibrants and the internal standards can be found in the SI_A: Table S8.

Unlike response factor-based semiquantification methods, which rely on surrogate standards for concentration estimation, or an approach that incorporates response factors with molar mass corrections, normalization provides a practical solution for monitoring relative changes over space and time.^35,36^ By correcting for instrumental variability using internal standards, this method enables trend analysis across different locations and years without requiring extensive calibration curves or structurally similar reference compounds. While this approach does not involve the use of target compound concentrations, it provides relative trends of suspect compounds.

## 3 Results and Discussion

### 3.1 Overview of Identified PFAS

The current study categorizes the findings into two primary groups: *Legacy PFAS* and *Emerging PFAS*. The *Legacy* PFAS consist of well-known compounds, including perfluoroalkyl carboxylic acids, perfluoroalkyl sulfonic acids, fluorotelomer sulfonic acids, perfluorooctane sulfonamide acetic acids, and perfluorinated alkyl sulfonamides. All other compounds identified in this study are classified as *Emerging* PFAS, representing newer or less studied substances that are gaining attention due to their presence in the environment.

A total of 78 PFAS were detected in the analyzed bream liver samples, with 68 classified into 12 different homologue groups and 10 that did not fit into any homologue series. Of the 78 detected compounds, 19 were target compounds quantified by Rupp et al.^4^ Additionally, 16 compounds were homologues related to these target analytes, while 43 were neither part of the target analysis nor target-derived homologues. The identification confidence of our findings was classified according to Charbonnet et al., 2022.^37^ Our identified PFAS are listed in the SI_C and may support future suspect screening of PFAS.

Among the 78 identified PFAS in this study, 26 compounds were confirmed at confidence level (CL) 1, ensuring the highest confidence in their identification through exact mass, isotope pattern, retention time, and fragment ion mass spectrum to an analytical standard. Additionally, 14 compounds were classified at CL 2, specifically at CL 2b and 2c, where probable structures were identified based on diagnostic fragmentation evidence and homologue patterns. For 9 compounds, CL 3 was achieved, indicating candidate structures supported by fragment or circumstantial evidence. The remaining 29 compounds were identified at CL 4. In some cases, we did obtain fragmentation data, but the lack of sufficient or diagnostic fragments prevented their classification to higher confidence levels. For more information regarding the identified compounds, refer to Table 1. For detailed identification information and chemical identifiers, refer to SI_A: Tables S9–S23.

**Table 1:**
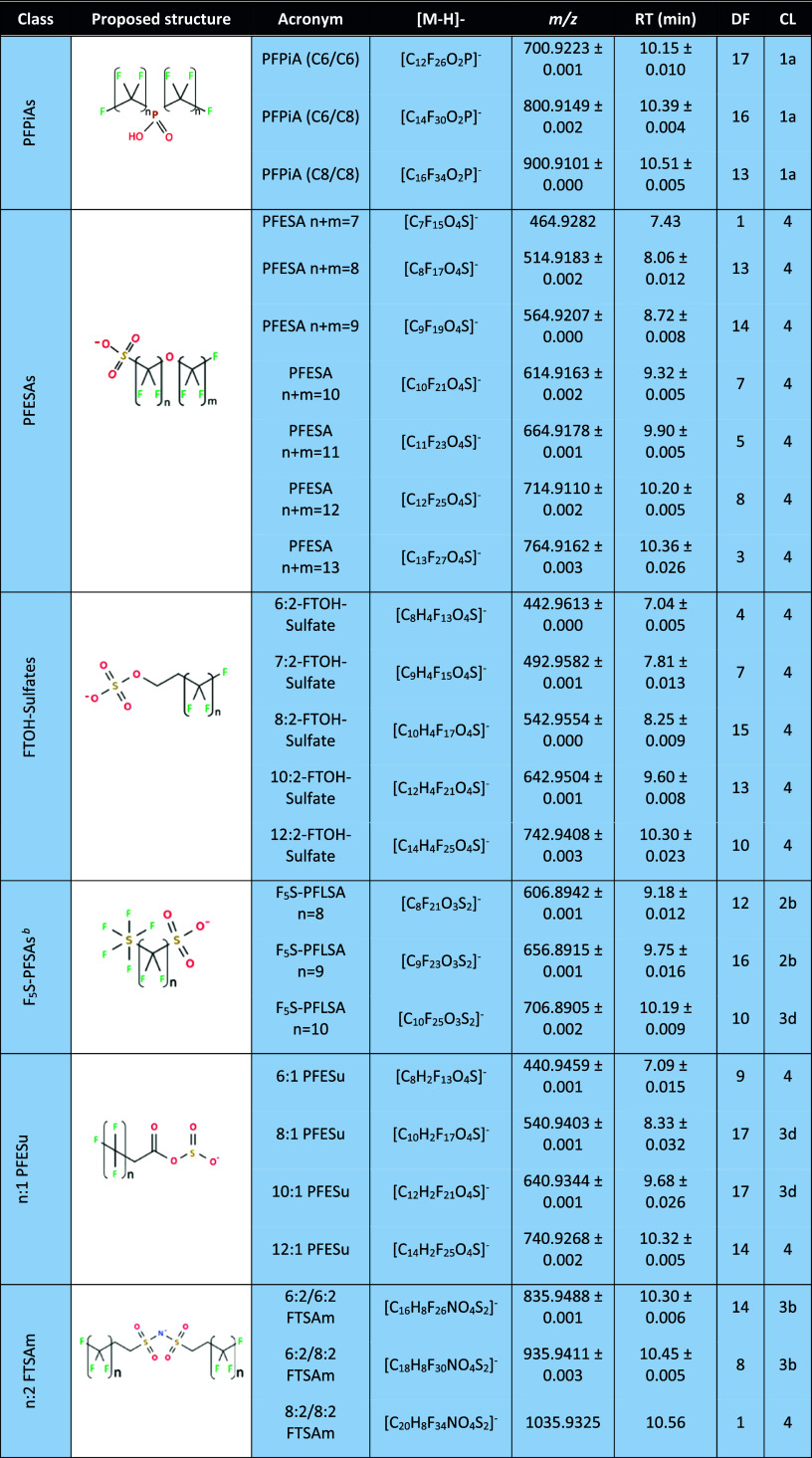

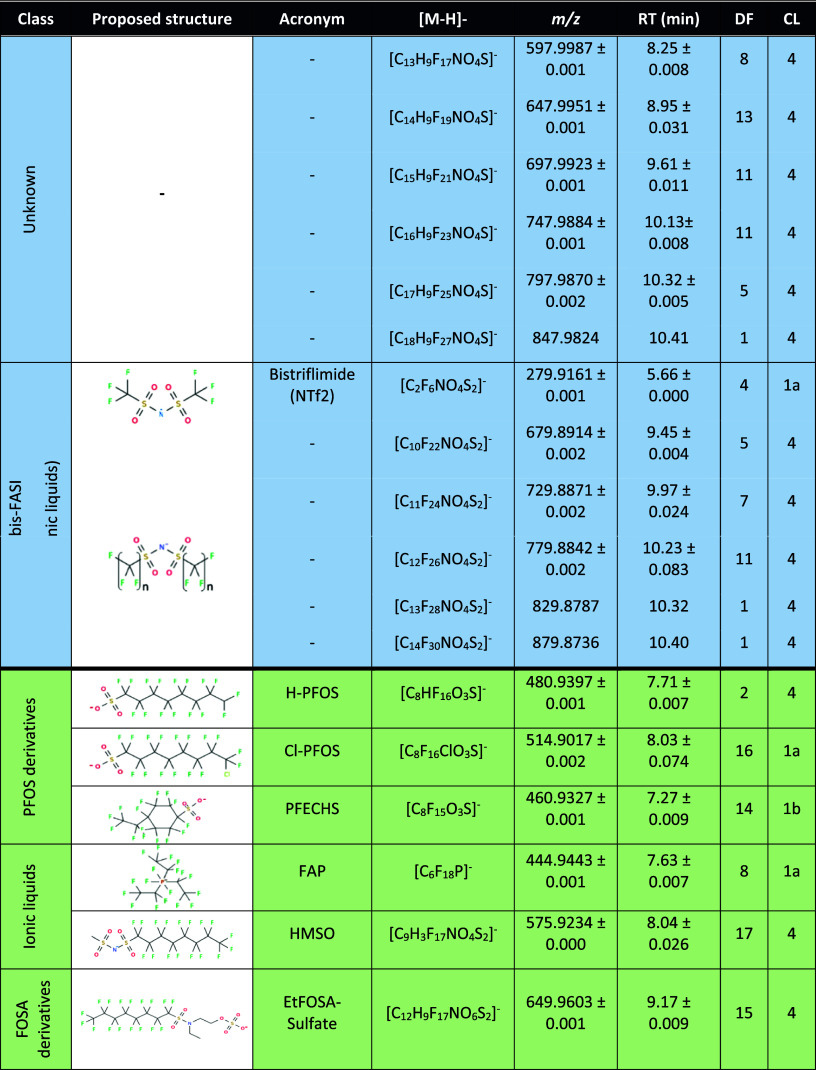
Summary of 43 *Emerging* PFAS^a^ Detected in Bream Liver Samples from Germany

a *Emerging* PFAS identified within homolog series (blue rows), and compounds not associated with any homolog class (green rows). RT stands for retention time, DF stands for detection frequency and CL stands for confidence level.

b Compounds listed as F_5_S-PFLSA refer to pentafluorosulfanyl-substituted linear perfluoroalkyl sulfonic acids. These are described in the text as F_5_S-PFSA, with “L” in PFLSA used in the table to emphasize the linearity of the perfluoroalkyl chain.

### 3.2 Identified *Legacy* PFAS

The perfluoroalkyl carboxylic acids (PFCAs; C_n_HF_2n‑1_O_2_, where *n* = 6–16), were consistently detected across the samples, with nine homologues (C6–C14) confirmed at CL 1a and C15–C16 identified at CL 2c (SI_A: Table S9, Figure S4). Similarly, the perfluoroalkyl sulfonic acids (PFSAs; C_n_HF_2n+1_O_3_S, where *n* = 4–14) displayed a uniform fragmentation pattern, leading to the confident identification of PFBS, PFHxS, PFOS, and PFDS at CL 1a. The remaining PFSAs were assigned to CL 2c due to their retention time alignment with target compound homologues (SI_A: Table S10, Figure S5). The detection of perfluoroalkyl acids (PFAAs) in fish liver may result from direct exposure or the metabolic transformation of precursor PFAS into terminal PFAAs. Both pathways have been reported in previous studies, suggesting that PFAA accumulation in biological systems can occur through either mechanism or a combination of both.^38–40^ PFAAs are regularly found in environmental and biological matrices.^38,39,41–43^

In addition to PFAAs, perfluorinated alkyl sulfonamides (FASAs, C_n_H_2_F_2n+1_NO_2_S, where *n* = 4–8) were distributed across all analyzed samples (SI_A: Tables S11–12, Figures S6–7). Perfluorooctane sulfonamide (FOSA, C_8_H_2_F_17_NO_2_S) was verified using an analytical standard, while the remaining homologues—FBSA, FPeSA, FHxSA, and FHpSA—were classified at CL 3c, based on their consistent incremental retention time shifts and a shared fragment ion at *m*/*z* 77.96, corresponding to [NO_2_S]^−^ (SI_A: Figure S6). FOSA, a known degradation product of sulfonamide-based precursors, is widespread across various environmental media.^19,44–46^ Additionally, the three compounds perfluorooctane sulfonamidoacetic acid (FOSAA), *N*-methyl perfluorooctane sulfonamidoacetic acid (MeFOSAA), and N-ethyl perfluorooctane sulfonamidoacetic acid (EtFOSAA) were identified and confirmed using analytical standards (CL 1a).

The telomer-based fluorotelomer sulfonic acids (FTSAs, C_n_H_5_F_2n‑3_O_3_S, where *n* = 8, 10, 12, 14, 16) further highlighted the diversity of PFAS in the bream liver samples. Among these, 6:2 FTSA and 8:2 FTSA were confirmed at CL 1a, while the remaining homologues were identified at CL 2c (SI_A: Table S13, Figure S8).

### 3.3 Identified *Emerging* PFAS

In recent years, the environmental presence of PFAS has received increasing attention due to their persistence, bioaccumulation potential, and widespread distribution across various ecosystems.^47,48^ While the regulation and study of well-known PFAS have advanced significantly, a growing concern lies in the emergence of lesser-known PFAS.^13,49–51^ The following section focuses on the identification and elucidation of these *Emerging* PFAS (Table 1).

#### 3.3.1 Detection of *Emerging* PFAS Homologues

A group of three homologues was identified as perfluoroalkyl phosphinic acids (PFPiAs). For PFPiA C6/C6, a fragment was observed at *m*/*z* 400.9411, corresponding to C_6_F_14_O_2_P^–^, while for the C8/C8 homologue, a fragment at *m*/*z* 500.9337 was detected, corresponding to C_8_F_18_O_2_P^–^. In the case of C6/C8, both of these fragments were observed, further supporting the identification of this homologue. This group exhibited a uniform fragmentation pattern that was consistent with the analytical standard used, leading to their confirmation at CL 1a (SI_A: Table S14, Figure S9).

A group of seven homologues was tentatively identified as perfluoroalkyl ether sulfonic acids (PFESAs, C_n_HF_2n+1_O_4_S, where *n* = 7–13). The molecular formula for the C9 homologue was validated based on its isotope pattern, which matches the proposed molecular formula as shown in SI_A: Figure S10a. Due to the lack of diagnostic fragments, the group was classified with CL 4. We provided PubChem database entries that demonstrate the structural plausibility of the group (SI_A: Table S15).

We identified a homologue series of fluorotelomer sulfates (FTOH-Sulfates, C_n_H_5_F_2n‑3_O_4_S, where *n* = 8, 9, 10, 12, 14). The identification for this group was assigned CL 4 due to the detection of only one fragment (the loss of -HF), specifically for 8:2 FTOH-Sulfate at *m*/*z* 522.9509 (SI_A: Table S16, Figure S11). Martin et al. investigated fluorotelomer alcohol (FTOH) transformation pathways using isolated rat hepatocytes and found that FTOH-sulfate and FTOH-glucuronide were the primary metabolites of 8:2 FTOH, indicating these conjugates could serve as qualitative markers of exposure to FTOHs.^52,53^ Detection of FTOH-Sulfates in our study suggests potential likelihood of exposure to FTOHs or FTOH-based PFAS such as polyfluoroalkyl phosphate esters (PAPs).

Derivatives of PFSAs, specifically pentafluorosulfide perfluoroalkyl sulfonic acids (F_5_S-PFSAs), were identified in bream liver samples through precursor ion mass matching, revealing the presence of three homologues (C8–10) (SI_A: Table S17). This identification was further supported by fragment ion data (SI_A: Figure S12), with C8 and C9 homologues showing clear fragments corresponding to their perfluorinated alkyl chains. Specifically, *m*/*z* 279.9435, 379.9371, and 479.9312 were detected, corresponding to SO_3_(CF_2_)_4_^–^, SO_3_(CF_2_)_6_^–^, and SO_3_(CF_2_)_8_^–^, respectively. For the C9 homologue, a diagnostic fragment at *m*/*z* 176.9606 was detected, corresponding to the F_5_SCF_2_ group. This fragment further confirms the presence of the pentafluorosulfide group, reinforcing the identification of the F_5_S-PFSA. Due to the low intensity, no fragments were detected for C10 homologue. The detection of F_5_S-PFSA in these samples is consistent with previous reports of its presence in groundwater affected by aqueous film-forming foam (AFFF), as well as in aquatic and terrestrial invertebrates.^54–56^ Furthermore, the fragmentation patterns observed in this study are consistent with the findings of Barzen-Hansen et al.,^52^ who initially reported similar fragmentation behavior for F_5_S-PFSAs in AFFF-impacted water. Their study also highlighted the diagnostic significance of fragments like *m*/*z* 176.9606, corresponding to the F_5_SCF_2_ group. Similarly, Zweigle et al.^54^ identified the F_5_S-PFSA group in PFAS-contaminated soil. These results demonstrate the pervasive presence of F_5_S-PFSAs across various environmental compartments, including soil, groundwater, and aquatic organisms. This highlights their persistence, bioavailability, and potential for trophic transfer. Further investigation into their environmental fate, bioaccumulation potential, and ecological impact is crucial to better understand their role in PFAS contamination.

A set of four homologues (n:1 PFESu, Table 1) was detected within the mass range spanning *m*/*z* 440.9472 to *m*/*z* 740.9280, exhibiting a consistent mass difference of 99.9936 Da between each homologue, indicative of the C_2_F_4_ moiety (SI_A: Table S18). Utilizing accurate mass measurement, a molecular ion formula was systematically deduced and subsequently validated through the isotope pattern analysis, resulting in the elemental composition of C_n_H_2_F_2n‑3_O_4_S (*n* = 8, 10, 12, 14). Molecular formulas were queried against the PubChem database, resulting in a match for the C10 homologue, proposed as methyl-1,2,3,3,4,4,5,5,6,6,7,7,7-tridecafluoro-1-oxoheptane-2-sulfonate. However, this structure is unlikely to be correct, as the compound lacks an ionizable site necessary for detection in ESI negative mode. For the C10 and C12 homologues, three fragment ions were observed, characterized by the general molecular formulas C_n_F_2n‑3_ and C_n_F_2n‑5_, and an additional unsaturated fluorinated alkyl chain. Since these fragments are not structurally diagnostic, the tentative identification for this group of compounds is based on a common fragmentation pattern, as noted by Hensema et al., who detected the C8 homologue in surface water samples applying a fragment-flagging approach.^20^ In our study, three additional homologues, C10 [*m*/*z* 540.9408], C12 [*m*/*z* 640.9344], and C14 [*m*/*z* 740.9280] were identified in bream livers and are reported for the first time (Table 1, SI_A: Figure S13).

A group consisting of three homologues emerged (n:2 FTSAm (n = 16, 18, 20) in Table 1), exhibiting a pronounced signal in the samples obtained from the Elbe and Rhine Rivers. The compound at *m*/*z* 835.9494 was identified as a fluorinated compound based on several key observations (SI_A: Table S19, Figure S14). We deduced the molecular formula C_16_H_8_F_26_NO_4_S_2_ based on the high mass accuracy of FT-ICR MS (1.2 ppm), confirmed by its isotope pattern. The fragment ions observed at *m*/*z* 405.9778 [C_8_H_4_F_12_NO_2_S]^−^, 385.9714 [C_8_H_3_F_11_NO_2_S]^−^, 365.9652 [C_8_H_2_F_10_NO_2_S]^−^, 345.9590 [C_8_HF_9_NO_2_S]^−^ in SI_A: Figure S13 indicate a growing degree of unsaturation on the carbon chain, corresponding to subsequent HF losses. This fragmentation pattern aligns with the recent study by Schüßler et al., which identified these compounds for the first time in AFFF-contaminated soil samples.^57^ The consistency between these independent studies highlights the need for further research on their environmental fate and potential risks. During the verification step, an unanticipated signal corresponding to C_16_H_8_F_26_NO_4_S_2_^–^ [*m*/*z* 835.9494] was detected in the analytical standard of 1-octanesulfonamide, N-[3-(dimethylamino)­propyl]-3,3,4,4,5,5,6,6,7,7,8,8,8-tridecafluoro- (Capstone A). Accordingly, the proposed final structure is likely formed through the cleavage of Capstone A at the sulfonamide site, followed by its recombination with the polyfluorinated sulfate part of Capstone A. As a result, this group is classified as 3b (SI_A: Table S19). One plausible explanation for the detection of the compound in the analytical standard is that it could be a byproduct originating from the manufacturing process.

A group consisting of six homologues (Unknown in Table 1) was detected in the liver samples (SI_A: Table S20, Figure S15), however, no fragments were observed to aid in structural identification. This series was previously detected in marine mammals^13^ and proposed to have a molecular ion formula of C_13_H_9_F_17_NO_4_S^–^ (*m*/*z* 597.9969). Out of the six homologues, only C13 and C15 were found in the PubChem database (SI_A: Table S20). To further support the identification process, additional details on the isotope pattern distribution for the C13 homologue are shown in SI_A: Figure S15.

A set of six homologues, bis­(perfluoroalkyl)­sulfonimides (bis-FASI in Table 1), was initially identified through a suspect screening process, agreeing with the general molecular ion formula C_n_F_2n+2_NO_4_S_2_ (n = 2, 10–14) (SI_A: Table S21). The C2 homologue, known as Bistriflimide (NTf2), was verified using an analytical standard (SI_A: Figure S16). Homologues C10 – C14 were further confirmed through isotope pattern analysis and retention time alignment with the C2 homologue, providing additional support for their identification.

NTf2 has been previously detected in environmental samples.^58–60^ Wang et al.^58^ identified NTf2 in seawater but noted its absence in biosamples, suggesting that it may have low bioaccumulation potential. Neuwald et al. reported the presence of NTf2 in German drinking water.^61^ In our study, NTf2 was detected in liver samples, indicating its presence in freshwater organisms in Germany. Furthermore, McDonough et al.^62^ identified homologues ranging from C6 to C12 using NTS. In our study, fragmentation analysis revealed a single detectable fragment for the C12 homologue at *m*/*z* 396.9429 (SI_A: Figure S16), corresponding to C_6_F_13_SO_2_N^–^, consistent with the findings of McDonough et al.^62^ Their study also demonstrated that these compounds were not symmetrical dimers but instead displayed varying chain lengths. Due to the limited fragmentation observed in our data set, we were unable to confirm this variability in chain lengths reported by McDonough et al. Notably, homologues C10, C12, and C14 are listed in the PubChem database, offering further reference points for their molecular identity. It is important to note that bis-FASI compounds are widely recognized as components of ionic liquids, as they can function as anions when paired with suitable cations. For this reason, we have emphasized their ionic liquid nature in this study, even though their specific role in an ionic liquid system is not explicitly addressed here.

#### 3.3.2 *Emerging* PFAS Not Associated with Any Homologue Class

Three PFOS derivatives, H-PFOS (CL 4), Cl-PFOS (CL 1a), and PFECHS (CL 1b), were identified. H-PFOS was observed eluting between 6.90 to 7.58 min (SI_A: Figure S17), indicating the presence of six isomers, as highlighted in the chromatogram, where the molecular ions with *m*/*z* 480.94 eluted at different times. The molecular formula of H-PFOS was confirmed through isotope pattern analysis, which is detailed in SI_A: Figure S16. For Cl-PFOS, signals were detected at three different retention times (SI_A: Figure S17), displaying a characteristic chlorine isotope pattern. However, none of the eluting peaks aligned with the analytical standard, sodium 8-chloroperfluoro-1-octanesulfonate, which eluted at 8.21 min. Differences in retention times suggest structural variations, potentially due to branching, which may cause shifts in compound elution. However, the absence of detectable fragments prevented further structural characterization. This limitation applies to the multiple peaks observed for both Cl-PFOS and H-PFOS, leaving their exact structural differences unresolved. The third and final PFOS derivative detected in this study is PFECHS, which was identified in the bream liver samples with highest confidence, CL 1b (SI_A: Figure S17).

FAP (tris­(pentafluoroethyl)­trifluorophosphate), the anion of EMIM FAP, an ionic liquid that combines the 1-ethyl-3-methylimidazolium (EMIM) cation with a fluorinated phosphate (FAP) anion, was identified in the bream liver samples (SI_A: Figure S18a). Notably, the compound displayed a fragmentation pattern consistent with the analytical standard, achieving confirmation at CL 1a. The compound was first reported in water samples by Neuwald et al. (2020), marking its initial detection in aquatic environments.^61^ It is now reported for the first time in a living organism, specifically in fish samples in the current study.

1,1,2,2,3,3,4,4,5,5,6,6,7,7,8,8,8-Heptadecafluoro-*N*-methylsulfonyloctane-1-sulfonamide (HMSO in Table 1) was tentatively identified based on its isotope pattern (SI_A: Figure S18b). Two fragment ions were observed for this compound, one at *m*/*z* 92.9878, corresponding to CH_3_NO_2_S^–^, and another at *m*/*z* 156.9498, corresponding to CH_3_NO_4_S_2_^–^. Although these two fragments could be considered elucidative, the mass accuracy for those fragments exceeded 5 ppm, likely due to the low intensity of the fragment ion signals. This reduced accuracy makes the identification less definitive, even with the observed fragments, leading to its classification at CL 4. Notably, many tentatively identified compounds exhibit a bissulfonyl imide bridge (SI_A:, S21, S23), which is a common structure in ionic liquids.^63^ One potential source of contamination with ionic liquids could be the use of fluorinated compounds in lithium-ion batteries.^64^ For these compounds to accumulate in fish liver, they must travel through the environment and water, eventually building up in biota without being metabolized. However, there is limited information on the fate of these substances or the formation of new ones during recycling, which depends on battery chemistries and recycling conditions, as highlighted in the recent review by Rensmo et al. (2023).^64^

Finally, the tentatively identified 2-[ethyl­(1,1,2,2,3,3,4,4,5,5,6,6,7,7,8,8,8-hepta­deca­fluoro­octyl­sulfonyl)­amino]­ethyl hydrogen sulfate (EtFOSA-Sulfate) was detected in the bream liver samples (SI_A: Figure S21). This identification was based on the presence of a specific fragment ion *m*/*z* 525.98 [C_10_H_5_F_17_NO_2_S]^−^, which aligns with the peak shape of the molecular ion [C_12_H_9_F_17_NO_6_S_2_]^−^ (Table 1, SI_A: Figure S18c), leading to its assignment as CL 4. This classification was primarily due to the reliance on single-fragment evidence and the absence of any corresponding homologues for this compound within our data set. For more information about FAP, HMSO and EtFOSA-Sulfate, refer to SI_A: Table S23.

However, it is important to keep in mind that the observed signal coelution (SI_C: Co-eluting compounds) in this study may be attributed to the measurement method employed. After 9.5 min, chromatographic resolution declined, likely causing coelution of compounds with similar hydrophobicity. While overlapping signals are common in homologue series, a consistent pattern across the series would suggest in-source fragments or adducts. Since this pattern was not observed, the signals likely represent distinct compounds rather than fragments (SI_A: Figure S19). Additionally, coeluting signals can be distinguished by their fragment ions eluting simultaneously with parent ions, maintaining a well-defined peak shape (SI_A: Figure S20). However, without analytical standards the possibility of in-source fragmentation cannot be entirely ruled out.

### 3.4 Spatial and Temporal Occurrence of PFAS

The sample from Koblenz (2008) exhibited the highest unexplained portion in the TOPA, prompting further analysis of identified PFAS in the Koblenz samples from 1996 to 2020. Since analytical standards were not available for many of the identified PFAS, a normalization-based approach was employed, allowing compound-specific comparison of relative concentrations of *Emerging* PFAS between samples (SI_C: PFAS suspect summary). Of the 10 compounds selected through quartile analysis, 9 belong to four homologue groups, while one compound (FAP) is a distinct compound that does not fall within any homologue series (Figure 1).

**Figure 1:**
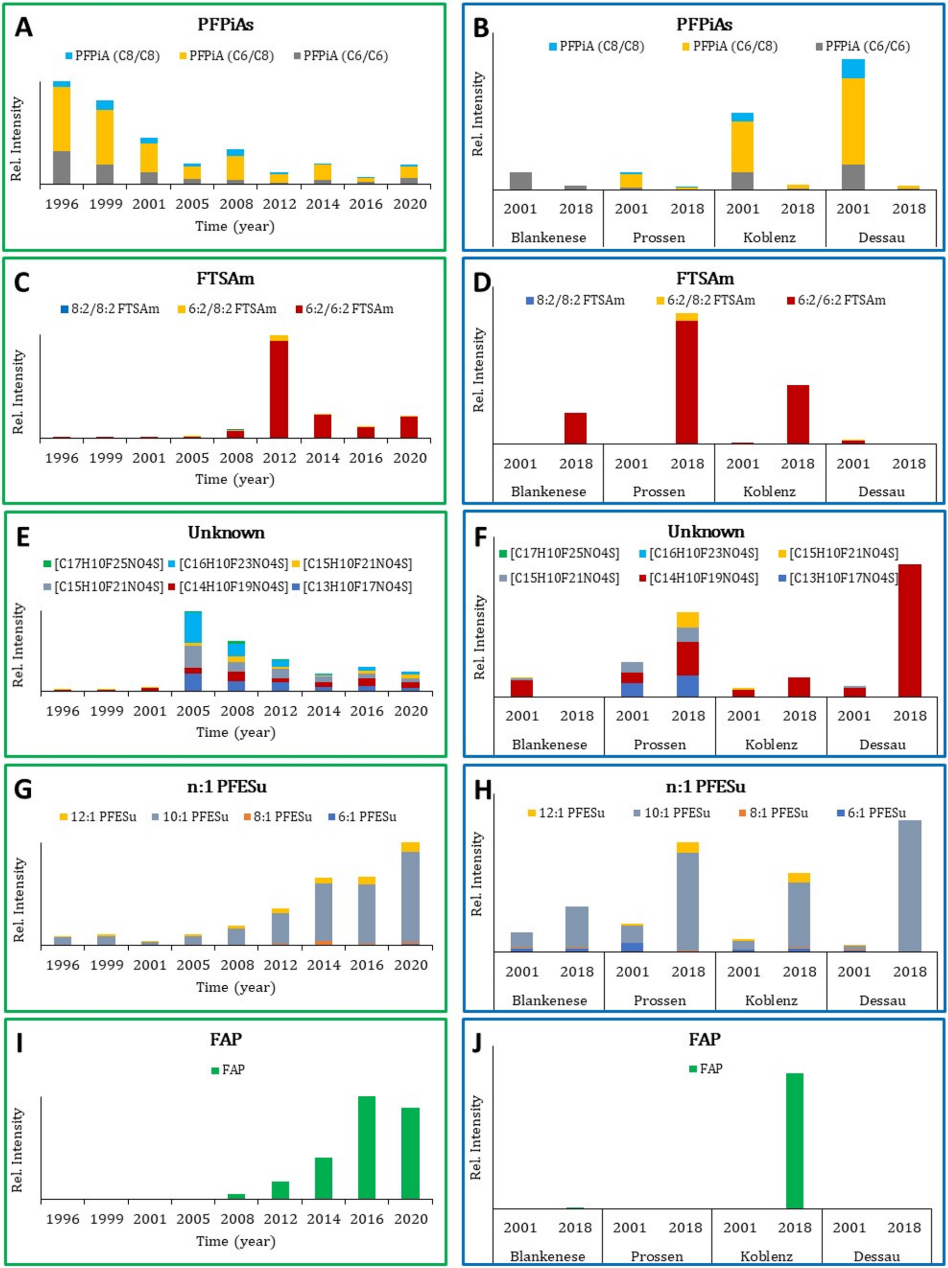
A, C, E, G, I: Temporal trends of PFAS in bream liver samples from Koblenz (River Rhine) between 1996 and 2020, illustrating the trends of four homologue series—PFPiAs, FTSAm, Unknown, and n:1 PFESu—alongside one individual compound (FAP). B, D, F, H, J: Spatial (and temporal) comparison of the four homologue series and FAP across four different locations at two time points (2001 and 2018). The *y*-axis represents the normalized relative intensity (dimensionless).

Among the PFAS shown in Figure 1A, PFPiAs are the only group that – from a production point of view (starting in the mid-20th century) – can be considered *Legacy* PFAS. Their levels declined over the whole monitored period, suggesting that their production and use were reduced, coinciding with the phase-out of the PFOS-based chemistry by 3 M around the year 2000.^65^ While this decline may be attributed to reduced emissions, biotransformation in organisms could also play a role. Previous studies have shown PFPiAs undergo metabolism in fish liver, with transformation products detected in high concentrations, suggesting the liver is a key organ for degradation.^66–68^ However, no known transformation products were identified in this study, leaving uncertainty regarding whether degradation alone accounts for this trend. Expanding the analysis to four locations (Koblenz, Blankenese, Prossen, and Dessau) over two time points (2001 and 2018) revealed a consistent decline in PFPiA presence across all sites (Figure 1B), reinforcing the idea that the trend is regional rather than site-specific. The most prevalent homologue, C6/C8 PFPiA, exhibited lower intensities at all sites in 2018 compared to 2001.

The FTSAm and Unknown groups, in the contrary, were hardly present at the beginning of our time trend (Figure 1C and E). A steep rise and peak of their levels in Koblenz around 2005 (Unknown) and 2012 (FTSAm) may indicate their use as replacement products during the aforementioned phase-out of the PFOS-chemistry. However, levels of FTSAm and Unknown homologues thereafter showed a gradual decline and stabilization toward 2020. Notably, FTSAm compounds, particularly 6:2/6:2 FTSAm, showed much lower levels in 2001 compared to 2018 in Blankenese, Prossen, and Koblenz, whereas in Dessau, they were detected in 2001 but not in 2018 (Figure 1D). Similarly, the Unknown group (Figure 1F) showed higher levels in Prossen, Koblenz, and Dessau in 2018 as compared to 2001, while Blankenese showed hardly any presence in 2018, suggesting regional differences in contamination sources or environmental conditions.

In contrast to declining and transitional groups, n:1 PFESu and FAP showed a continuous increase in Koblenz over the monitored period (Figure 1G and I), suggesting either ongoing and potentially increasing industrial use and/or environmental persistence. Among the n:1 PFESu group, 10:1 PFESu was the most abundant homologue (Figure 1G). A related homologue (6:1 PFESu) was previously identified in surface waters in The Netherlands, indicating a broader environmental presence.^20^ In our study, the 10:1 homologue was already detected in 1996 and increased steadily until 2020, reinforcing concerns regarding bioaccumulation and persistence in aquatic environments. When comparing locations, n:1 PFESu homologues were present at all sites in both years, showing higher levels in 2018 compared to 2001, with 10:1 PFESu being the dominant homologue (Figure 1H).

FAP was first detected in 2008, indicating a widespread industrial introduction only in the early 2000s (Figure 1I). As a fluorinated ionic liquid, FAP is valued for its stability and nonvolatility, particularly in industrial applications such as electrochemistry.^69,70^ FAP was detected almost exclusively in Koblenz in 2018 (Figure 1J), suggesting a local source, possibly industrial discharges, along the Rhine river. However, a small relative intensity was also observed in Blankenese (Elbe river, downstream of Hamburg), possibly due to local emissions or environmental redistribution. The Rhine river discharges into the North Sea, where tidal inflow reaches the Elbe estuary all the way up to Blankenese, allowing contaminants to mix and redistribute through marine currents and tides, potentially explaining the presence of FAP in Blankenese.

The river Elbe is the only river that was sampled twice, at Prossen, just after crossing the border from the Czech Republic to Germany, and roughly 600 km downstream at Blankenese, shortly before discharging into the North Sea. FTSAm, Unknown and n:1 PFESu showed markedly higher levels at Prossen than at Blankenese, independent of the investigated year (2001 or 2018). This indicates, that the main part of the contamination enters the river (and the fish) already quite far upstream and is probably then diluted along the way or by the above-mentioned infiltration through North Sea water at Blankenese.

There are certain limitations in this study. The normalization approach employed here facilitates comparison across different locations and may exaggerate small variations, making changes appear more significant than they are (e.g., Unknown group). Despite this limitation, the findings offer valuable insights into the presence and trends of specific compounds. Future studies would benefit from multiple replicate measurements to better account for variability and enable more accurate statistical analyses.

## 4 Implications

The successful use of LC-QTOF MS and FT-ICR MS to identify a large variety of PFAS highlights the importance of high-resolution mass spectrometry in discovering emerging and unknown PFAS in complex biota samples, e.g. fish liver. By analyzing archived samples from periods before PFAS were regulated and comparing them with recent samples, both *Legacy* and *Emerging* substances could be uncovered over extended timeframes. Our results provide insight into the presence and evolution of PFAS contamination over time. While *Legacy* PFAS appear to decline, it is crucial that monitoring efforts continuously incorporate newly identified compounds to remain effective in understanding environmental contamination and exposure. To better capture the extent of PFAS contamination, future screenings should integrate current findings and aim for quantitative analysis.

## Supplementary Material







## Notes

SI_A: Sample information; preparation of standard solution; devices and software; instrumental parameters, data processing; peak areas; chemical identifiers; and MS^E^ spectra of identified PFAS (PDF)
SI_B: Suspect list “PFAS Master List of PFAS (Retired)” (XLSX)
SI_C: Summary of identified PFAS and their chemical identifiers (XLSX)

## $

Laboratory of Clinical Biochemistry and Metabolism, Department of General Pediatrics, Adolescent Medicine and Neonatology, Faculty of Medicine, University of Freiburg, 79106 Freiburg, Germany

## Glossary

6:2 FTSA: 6:2 Fluorotelomer sulfonic acid
8:2 FTOH-Sulfate: 8:2 Fluorotelomer alcohol sulfate
8:2 FTSA: 8:2 Fluorotelomer sulfonic acid
ACN: acetonitrile
Bis-FASI: Bis­(perfluoroalkyl)­sulfonimide
Capstone A: 1-Octanesulfonamide, N-[3-(dimethylamino)­propyl]-3,3,4,4,5,5,6,6,7,7,8,8,8-tridecafluoro
CASI: Continuous accumulation of selected ions (Q-isolation)
CL: Confidence level
Cl-PFOS: Chloroperfluorooctanesulfonate
Da: Dalton
EMIM: 1-Ethyl-3-methylimidazolium
EMIM FAP: 1-Ethyl-3-methylimidazolium tris­(pentafluoroethyl)­trifluorophosphate
EOF: Extractable organic fluorine
EtFOSA: N-Ethyl perfluorooctane sulfonamidoacetic acid 2-[ethyl­(1,1,2,2,3,3,4,4,5,5,6,6,7,7,8,8,8-heptadecafluorooctylsulfonyl)­amino]­ethyl hydrogen sulfate
EtFOSA-Sulfate: 2-[Ethyl­(1,1,2,2,3,3,4,4,5,5,6,6,7,7,8,8,8-heptadecafluorooctylsulfonyl)­amino]­ethyl Hydrogen Sulfate
F_5_S-PFSAs: Pentafluorosulfide perfluoro sulfonic acids
FAP: Tris­(pentafluoroethyl)­trifluorophosphate
FASAs: Perfluorinated alkyl sulfonamides group
FBSA: Perfluorobutane sulfonamide
FHpSA: Perfluoroheptane sulfonamide
FHxSA: Perfluorohexane sulfonamide
FOSA: Perfluorooctane sulfonamide
FOSAA: Perfluorooctane sulfonamide acetic acid
FPeSA: Perfluoropentane sulfonamide
FT-ICR MS: Fourier transform ion cyclotron resonance mass spectrometry
FTSAm: Fluorotelomer sulfonamide
FTOHs: Fluorotelomer alcohols
FTOH-Sulfates: Fluorotelomer sulfates
FTSAs: Fluorotelomer sulfonic acids
h: Hours (SI unit)
H-PFOS: Hydrogen-substituted perfluorooctanesulfonate
HMSO: 1,1,2,2,3,3,4,4,5,5,6,6,7,7,8,8,8-heptadecafluoro-*N*-methylsulfonyloctane-1-sulfonamide
HRMS: High-resolution mass spectrometry
IAT: Ion accumulation time
ILs: Ionic liquids
LC-QTOF MS: Liquid chromatography–quadrupole time of flight mass spectrometry
*m*/*z*: Mass-to-charge ratio
MeFOSAA: *N*-Methyl perfluorooctane sulfonamidoacetic acid
min: Minutes (SI unit)
MS: Mass spectrometry
MS^E^: Mass spectrometry elevated energy
PAP: Polyfluoroalkyl phosphate esters
PFAAs: Perfluoroalkyl acids
PFAS: Per- and polyfluoroalkyl substances
PFBS: Perfluorobutanesulfonate
PFCAs: Perfluorocarboxylic acids
PFDS: Perfluorodecanesulfonate
PFECHS: Perfluoroethylcyclohexanesulfonate
PFESAs: Perfluoroalkyl ether sulfonic acids
PFESu: Polyfluoroalkyl ester sulfinic acids
PFHxS: Perfluorohexanesulfonate
PFSAs: Perfluoroalkanesulfonic acids
PFOS: Perfluorooctanesulfonate
PFPiA C6/C6: Perfluoroalkyl phosphinic acid C6/C6
PFPiA C6/C8: Perfluoroalkyl phosphinic acid C6/C8
PFPiA C8/C8: Perfluoroalkyl phosphinic acid C8/C8
PFPiAs: Perfluoroalkyl phosphinic acids
PP: Polypropylene
ppm: Parts per million
Q3: Third quartile
QTOF: Quadrupole time-of-flight
s: Seconds (SI unit)
SI: Supporting Information
TOPA: Total oxidizable precursor assay
UHRMS: Ultrahigh resolution mass spectrometry
