# Supplementary material for: Retrospective Identification of Novel and Legacy Per- and Polyfluoroalkyl Substances in German Archived Fish Livers Using a Combined High-Resolution Mass Spectrometry Approach
Source: Environ Sci Technol. 2025 Jun 20;59(25):12865–77. doi: 10.1021/acs.est.4c11600 (PMC12224335; doi:10.1021/acs.est.4c11600)
Supplement: Supplementary file 1 [file es4c11600_si_001.pdf]

Supporting Information: SI_A

Retrospective Identification of Novel and Legacy Per- and Polyfluoroalkyl Substances (PFAS) in German Archived Fish Livers Using a Combined HRMS Approach

Silvia Dudášová[1], Urs Berger[1,$], Bettina Seiwert[1], Thorsten Reemtsma[1,2], Oliver J. Lechtenfeld[1,3] Qiuguo Fu[1*]

[1]Department of Environmental Analytical Chemistry, Helmholtz Centre for Environmental Research - UFZ, Permoserstraße 15, 04318 Leipzig, Germany.

[2] Institute for Analytical Chemistry, University of Leipzig, Linnéstrasse 3, 04103 Leipzig, Germany.

[3] ProVIS - Centre for Chemical Microscopy, Helmholtz Centre for Environmental Research - UFZ, Permoserstraße 15, 04318 Leipzig, Germany.

$ present address: Laboratory of Clinical Biochemistry and Metabolism, Department of General Pediatrics, Adolescent Medicine and Neonatology, Faculty of Medicine, University of Freiburg, 79106 Freiburg, Germany

Corresponding Author

Qiuguo Fu - Department of Environmental Analytical Chemistry, Helmholtz-Centre for Environmental Research - UFZ, Permoserstrasse 15, 04318 Leipzig, Germany; https://orcid.org/0000-0002-4227-5948; Email: qiuguo.fu@ufz.de

Summary:

87pages, 54 figures, 24 tables.

Table of Contents

Figure S1 – The map of Germany highlights the collection sites. 5

Table S1 – Overview of analyzed samples. 6

Table S2 – List of target compounds. 7

Table S3 – Solution and solvents. 8

Table S4 – Summary of equipment and models. 9

Table S5 – Summary of softwares and platforms. 9

Table S6a – Instrumental parameters of LC-qTOF MS method. 10

Table S6b – Gradient table....11

Figure S2 – FT-ICRM MS method performance 13

Table S7 – Data processing steps....13

Figure S3 – Data processing pipeline. 16

Table S8 – Calibrant and IS....17

Table S9 – Tentatively identified perfluorocarboxylic acids and their chemical identifiers....19

Figure S4a – Chromatograms of PFHxA, PFHpA, PFOA, and PFNA. 20

Figure S4b – Chromatograms of PFDA, PFUnDA, PFDoDA, and PFTrDA. 21

Figure S4c – Chromatograms of PFTeDA, PFPeDA and PFHxDA.. 22

Figure S4d – Fragment ion mass spectra of PFDA. 23

Table S10 – Tentatively identified perfluoroalkane sulfonic acids and their chemical identifiers. 24

Figure S5a – Chromatograms of PFBS, PFHxS, PFOS, and PFDS....25

Figure S5b – Chromatograms of PFSA.. 26

Figure S5c – Ion fragment mass spectrum spectrum of PFOS. 27

Table S11 – Tentatively identified perfluoroalkane sulfonamides and their chemical identifiers. 28

Figure S6a – Chromatograms of FBSA, FPeSA, FHxSA, FHpSA, and FOSA....29

Figure S6b – Fragment ion mass spectrum of FOSA 30

Figure S6c – Chromatograms of FBSA, FPeSA, FHxSA, and FHpSA 31

Table S12 – Tentatively identified perfluorooctane sulfonamido acetic acids and their chemical identifiers. 32

Figure S7a – Chromatograms of FOSAA, MeFOSAA, and EtFOSAA 33

Table S13 – Tentatively identified fluorotelomer sulfonic acids and their chemical identifiers. 34

Figure S8a – Chromatograms of the n:2 FTSA group. 35

Figure S8b – Fragment ion mass spectrum (n:2 FTSA) 36

Figure S8c – Fragment ion mass spectrum (n:2 FTSA). 37

Table S14 – Tentatively identified perfluoroalkyl phosphinic acids and their chemical identifiers. 38

Figure S9a – Chromatograms of PFPiA (C6/C6), PFPiA (C6/C8), and PFPiA (C8/C8) 39

Figure S9b – The fragment ion mass spectrum of PFPiA (C6/C6) 40

Figure S9c – Fragment ion mass spectrum of PFPiA (C6/C8) 41

Figure S9d – Fragment ion mass spectrum of PFPiA (C8/C8). 42

Table S15 – Tentatively identified perfluoroether sulfonic acids 43

Figure S10a – Chromatograms of PFESA group 44

Figure S10b – Chromatogram of samples collected in 1996 and 2008 45

Table S16 – Tentatively identified fluorotelomer alcohol sulfates. 46

Figure S11 – Chromatograms of FTOH-Sulfates 47

Table S17 – Tentatively identified perfluoroalkyl pentafluorosulfanyl sulfonic acids and their chemical identifiers. 48

Figure S12a – Chromatogram F5S-PFLSA group. 49

Figure S12b – Fragment ion mass spectrum corresponding to F5S-PFLSA, *n* = 8. 50

Figure S12c – Fragment ion mass spectrum corresponding to F5S-PFLSA, *n* = 9 51

Figure S12d – Fragment ion mass spectrum corresponding to F5S-PFLSA, *n* = 9. 52

Table S18 – Tentatively identified polyfluoroalkyl sulfinates (n:1 PFESu) and their chemical identifiers. 53

Figure S13a – Chromatograms of four homologs categorized as n:1 PFESu. 54

Figure S13b – Fragment ion mass spectrum of 6:1 PFESu (*m/z* 440.9442) 55

Figure S13c – Fragment ion mass spectrum of 8:1 PFESu (*m/z* 540.9400) 56

Figure S12d – Fragment ion mass spectrum of 10:1 PFESu (*m/z* 640.9350) 57

Figure S12e – Zoomed fragment ion mass spectrum of the 12:1 PFESu 58

Table S19 – Tentatively identified polyfluoroalkyl sulfonyl sulfonamides and their chemical identifiers 59

Figure S14a – Chromatogram of n:2 FTSAm. 60

Figure S14b – Chromatogram of molecular ion (*m/z* 835.9493) 61

Figure S14c – Chromatogram of fragment ion mass spectrum for 6:2/6:2 FTSAm (*m/z* 835.9493) 62

Figure S14d – Cchromatogram of fragment ion mass spectrum for 6:2/6:2 FTSAm (*m/z* 835.9493). 63

Figure S14e – Chromatogram of fragment ion mass spectrum for 6:2/6:2 FTSAm (*m/z* 835.9493). 64

Figure S14f – Chromatogram of 6:2/8:2 FTSAm (*m/z* 935.9427) 65

Figure S14g – Fragment ion mass spectrum of 6:2/8:2 FTSAm (*m/z* 935.9427)....................66

Table S20 – Tentatively identified perfluoroalkyl sulfonyl amino acids. 68

Figure S15 – Chromatograms of "Unknown" group 69

Table S21 – Tentatively identified bis(perfluoroalkylsulfonyl)imides and their chemical identifiers. 70

Figure S16a – Chromatograms of bis-FASI....................71

Figure S16b – Fragment ion spectra of Bistriflimide 72

Figure S16c – Fragment ion mass spectrum of *m/z* 779.8857....................73

Figure S16d – Fragment ion mass spectrum and chromatogram of co-eluting compound 74

Table S22 – Tentatively identified PFOS derivatives and their chemical identifiers. 75

Figure S17a – Chromatogram of H-PFOS....................76

Figure S17b – Chromatogram of H-PFOS. 77

Figure S17c – Chromatogram and fragment ion mass spectrum of Cl-PFOS....................78

Figure S17d – Chromatogram and precursor ion mass spectrum of Cl-PFOS. 79

Figure S17e – Fragment ion mass spectrum of PFECHS 80

Table S23 – Tentatively identified perfluorinated compounds and their chemical identifiers. 81

Figure S18a – Fragment ion mass spectrum of FAP 82

Figure S18b – Fragment ion mass spectrum of HMSO 83

Figure S18c – Fragment ion mass spectrum for *m/z* 649.9607. 84

Figure S19 – Chromatogram of co-eluting signals....................85

Figure S20– Chromatograms of co-eluting signals....................86

1. Sample information

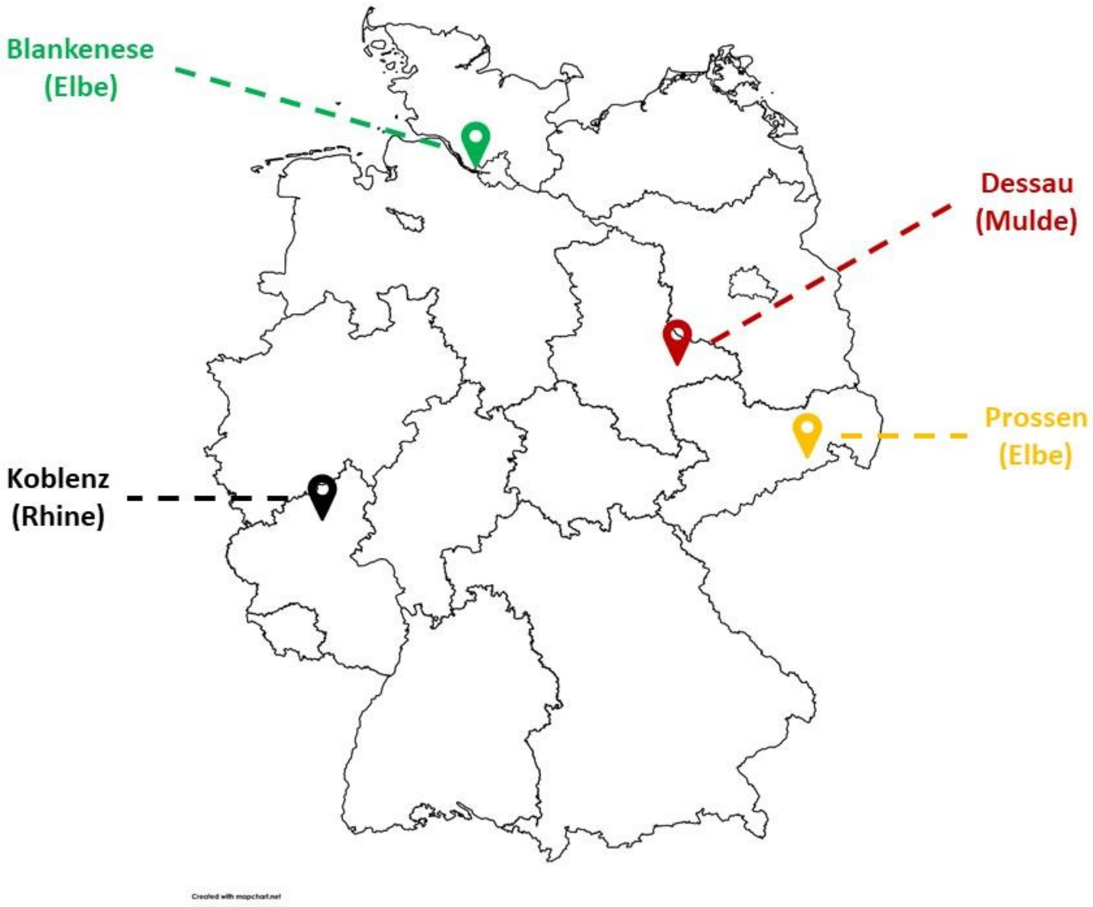

Figure S1 – The map of Germany highlights the collection sites of four bream liver samples in red.

Table S1 – Overview of analyzed samples.

**Sample (*Spec.*)**	**Sampling site**	**Sampling year**	**Sample pooled?**
Common bream liver *(Abramis brama)*	Bimmen (Rhine)	2001	Yes
Common bream liver *(Abramis brama)*	Bimmen (Rhine)	2018	Yes
Common bream liver *(Abramis brama)*	Dessau (Mulde)	2001	Yes
Common bream liver *(Abramis brama)*	Dessau (Mulde)	2018	Yes
Common bream liver *(Abramis brama)*	Koblenz (Rhine)	2001	Yes
Common bream liver *(Abramis brama)*	Koblenz (Rhine)	2018	Yes
Common bream liver *(Abramis brama)*	Koblenz (Rhine)	1996	Yes
Common bream liver *(Abramis brama)*	Koblenz (Rhine)	1999	Yes
Common bream liver *(Abramis brama)*	Koblenz (Rhine)	2005	Yes
Common bream liver *(Abramis brama)*	Koblenz (Rhine)	2008	Yes
Common bream liver *(Abramis brama)*	Koblenz (Rhine)	2012	Yes
Common bream liver *(Abramis brama)*	Koblenz (Rhine)	2014	Yes
Common bream liver *(Abramis brama)*	Koblenz (Rhine)	2016	Yes
Common bream liver *(Abramis brama)*	Koblenz (Rhine)	2020	Yes
Common bream liver *(Abramis brama)*	Prossen (Elbe)	2001	Yes
Common bream liver *(Abramis brama)*	Prossen (Elbe)	2018	Yes

Table S2 – List of target compounds with acronym, and corresponding PFAS family.

IUPAC	Acronym	Family	CAS RN	Manufacturer
2,2,3,3,4,4,5,5,6,6,6-undecafluorohexanoic acid	PFHxA	PFCA	307-24-4	Wellington
2,2,3,3,4,4,5,5,6,6,7,7,7-tridecafluoroheptanoic acid	PFHpA	PFCA	375-85-9	Wellington
2,2,3,3,4,4,5,5,6,6,7,7,8,8,8-pentadecafluorooctanoic acid	PFOA	PFCA	335-67-1	Wellington
2,2,3,3,4,4,5,5,6,6,7,7,8,8,9,9,9-heptadecafluorononanoic acid	PFNA	PFCA	375-95-1	Wellington
2,2,3,3,4,4,5,5,6,6,7,7,8,8,9,9,10,10,10-nonadecafluorodecanoic acid	PFDA	PFCA	335-76-2	Wellington
2,2,3,3,4,4,5,5,6,6,7,7,8,8,9,9,10,10,11,11,11-henicosafluoroundecanoic acid	PFUnDA	PFCA	2058-94-8	Wellington
2,2,3,3,4,4,5,5,6,6,7,7,8,8,9,9,10,10,11,11,12,12,12-tricosafluorododecanoic acid	PFDoDA	PFCA	307-55-1	Wellington
2,2,3,3,4,4,5,5,6,6,7,7,8,8,9,9,10,10,11,11,12,12,13,13,13-pentacosafluorotridecanoic acid	PFTrDA	PFCA	72629-94-8	Wellington
2,2,3,3,4,4,5,5,6,6,7,7,8,8,9,9,10,10,11,11,12,12,13,13,14,14,14-heptacosafluorotetradecanoic acid	PFTeDA	PFCA	376-06-7	Wellington
1,1,2,2,3,3,4,4,4-nonafluorobutane-1-sulfonic acid	PFBS	PFSA	375-73-5	Wellington
1,1,2,2,3,3,4,4,5,5,6,6,6-tridecafluorohexane-1-sulfonic acid	PFHxS	PFSA	355-46-4	Wellington
1,1,2,2,3,3,4,4,5,5,6,6,7,7,8,8,8-heptadecafluorooctane-1-sulfonic acid	PFOS	PFSA	1763-23-1	Wellington
1,1,2,2,3,3,4,4,5,5,6,6,7,7,8,8,9,9,10,10,10-henicosafluorodecane-1-sulfonic acid	PFDS	PFSA	335-77-3	Wellington
1,1,2,2,3,3,4,4,5,5,6,6,7,7,8,8,8-heptadecafluorooctane-1-sulfonamide	FOSA	FASA	754-91-6	Wellington
2-(1,1,2,2,3,3,4,4,5,5,6,6,7,7,8,8,8-heptadecafluorooctylsulfonylamino)acetic acid	FOSAA	FASAA	2806-24-8	Wellington
2-[1,1,2,2,3,3,4,4,5,5,6,6,7,7,8,8,8-heptadecafluorooctylsulfonyl(methyl)amino]acetic acid	MeFOSAA	FASAA	2355-31-9	Wellington
2-[ethyl(1,1,2,2,3,3,4,4,5,5,6,6,7,7,8,8,8-heptadecafluorooctylsulfonyl)amino]acetic acid	EtFOSAA	FASAA	2991-50-6	Wellington
3,3,4,4,5,5,6,6,7,7,8,8,8-tridecafluorooctane-1-sulfonic acid	6:2 FTSA	FTSA	27619-97-2	Wellington
3,3,4,4,5,5,6,6,7,7,8,8,9,9,10,10,10-heptadecafluorodecane-1-sulfonic acid	8:2 FTSA	FTSA	39108-34-4	Wellington
bis(1,1,2,2,3,3,4,4,5,5,6,6,6-tridecafluorohexyl)phosphinic acid	6:2/6:2 PFPiA	PFPiA	40143-77-9	Wellington
bis(1,1,2,2,3,3,4,4,5,5,6,6,7,7,7-pentadecafluoroheptyl)phosphinic acid	6:2/8:2 PFPiA	PFPiA	158986-67-5	Wellington
bis(1,1,2,2,3,3,4,4,5,5,6,6,7,7,8,8,8-heptadecafluorooctyl)phosphinic acid	8:2/8:2 PFPiA	PFPiA	40143-79-1	Wellington
1,1,1-trifluoro-*N*-(trifluoromethylsulfonyl)methanesulfonamide	NTf2	Bis-FASI	82113-65-3	abcr
Sodium trifluoromethanesulfonimide	FAP	-	377739-43-0	Merck
Sodium 8-chloroperfluoro-1-octanesulfonate	Cl-PFOS	PFSA	777011-38-8	Wellington
1,2,2,3,3,4,5,5,6,6-decafluoro-4-(1,1,2,2,2-pentafluoroethyl)cyclohexane-1-sulfonic acid	PFECHS	PFSA	646-83-3	Wellington

Table S3 – Solution and solvents.

Name	Abbreviation	CAS:	Manufacturer	Quality
Ammonium acetate	NH_4oAc	631-61-8	Biosolve (Valkenswaard, Netherlands)	≥99%
Methanol	MeOH	67-56-1	Biosolve (Valkenswaard, Netherlands)	≥99%
Acetonitrile	ACN	75-05-8	CHROMASOLV™	≥99.9%

1.2. Preparation of standard solution

Ammonium Acetate Dilution: Ammonium acetate (NH_4Ac) was prepared by diluting 20 µL of a 1M NH_4Ac solution with 4980 µL of MilliQ water in an ultraperformance liquid chromatography (UPLC) grade glass vial. This solution was used for subsequent analytical applications. **Trifluoromethanesulfonimide (TFMS) Solution:** TFMS was prepared by weighing 3.25 mg of the solid substance and dissolving it in 3.25 mL of methanol, ensuring complete dissolution via vortex mixing. The resulting solution had a concentration of 1 mg/mL. This was further diluted to 50 µg/mL by mixing 50 µL of the 1 mg/mL solution with 950 µL of methanol. **Diphenylphosphoryl azide (DPOSA) Solution:** A 1 mg/mL solution of DPOSA was diluted to 50 µg/mL for this study. The dilution was performed by combining 50 µL of the 1 mg/mL DPOSA solution with 950 µL of methanol. **Preparation of Mix A:** Mix A comprised all individual Wellington substances standardized to 50 µg/mL, along with equal concentrations of DPOSA and TFMS. To prepare Mix A, 10 µL of each of the 25 substances was combined, totaling 250 µL, which was then mixed with 750 µL of methanol. This produced a total volume of 1000 µL, where each substance was present at a final concentration of 500 ng/mL.

2. Devices and Software

Table S4 – Summary of equipment and models utilized in this study.

**Device**	**Supplier, model**
Analytical balance	Mettler, PM4800
Vortexer	Heidolph REAX 2000
MillQ water system	Merck, Direct 8

Table S5 – Summary of softwares and platforms utilized in this study.

**Platform**	**Supplier, version**
MassLynx	Waters, 4.1
TargetLynx	Waters, 4.1
Compass DataAnalysis	Bruker, 5.0
KNIME Analytics Platform	KNIME AG, 4.7.0
MZmine[1]	R. Schmid, S. Heuckeroth, et. al, 3.0
Pflow[2]	S. Dudášová, J. Wurz, et. al, 1.0

3. Chemical analysis

Table S6a – Instrumental parameters of LC-qTOF MS method.

Parameter	Value
Polarity	ESI-
Analyser	Sensitivity Mode
Capillary (kV)	0.8
Precursor mass range	50 Da to 1200 Da
Sampling Cone	40.0
Source Offset	80
Cone gas	Nitrogen
Collision gas	Argon
Source Temperature (°C)	120
Desolvation Temperature (°C)	600
Cone Gas Flow (L/Hr)	50.0
Desolvation Gas Flow (L/Hr)	1000.0
Source temperature	120 °C
Collision energy	15 to 45 eV
Ion energy	0.8
Column Temperature	45.0 °C
Flow rate (µL h-1)	240
Capillary (kV)	4.2
Nebulizer gass pressure (Bar)	1.0
Dry gas temperature (^{0}C)	250
Dry gas flow rate (L/min.)	8.0

Table S6b – Gradient table.

Time(min)	Flow Rate(mL/min)	%A	%B	Curve
Initial	0.35	90	10	Initial
1.5	0.35	90	10	6
4.5	0.35	35	65	6
8.25	0.35	20	80	6
8.26	0.35	0.1	99.9	6
9.5	0.35	0.1	99.9	6
11	0.35	90	10	6
11.01	0.35	90	10	6
15	0.35	90	10	6

%A: 2 mM ammonium acetate in water/methanol, 95/5, v/v

%B: 2 mM ammonium acetate in water/methanol/acetonitrile, 5/75/20, v/v/v

4. FT-ICR MS

4.1. Koblenz sample (Method A)

The analysis was conducted using an FT-ICR mass spectrometer (solariX XR, Bruker Daltonik GmbH, Germany) equipped with a 12 Tesla refrigerated, actively shielded superconducting magnet (Bruker Biospin, Wissembourg, France). Extracts were diluted 1:100 in a methanol/water mixture (1:1, v/v) and directly injected into the ESI source at a flow rate of 240 µL/h. Data acquisition was conducted in negative ionization mode under the following conditions: capillary voltage of 4.2 kV, nebulizer gas pressure of 1.0 bar, dry gas temperature of 250 °C, and dry gas flow rate of 8.0 L/min. A data size of 4 Megaword (147 start *m/z*, ~1.677 s transients) was used for analysis. Mass spectra were collected in the range *m/z* 150 – 2000, but segmented into predefined mass windows via Q-isolation (*m/z* 150-230, 230-270, 270-300, 300-400, 400-500, 500-600, 600-700, 700-800, 800-900, 900-1000, 980-2000), with constant 1000 ms ion accumulation time across all mass segments. For the bream liver sample, spectra from 11 mass windows were acquired, each containing 2,500 to 6,500 detected signals (S/N ≥ 4). Internal mass calibration was performed using a lipid list (*m/z* 87-1572, *n* = 473), achieving a mass accuracy of < 0.5 ppm (*n* = 71). This sample could not be reanalyzed with the newer method (Method B) because the sample was no longer available.

4.2. Blankenese, Dessau and Prossen samples (Method B)

The bream liver samples were measured using the same FT-ICR MS as described in Method A. The samples were acquired via segmented Q-isolation (overall *m/z* range: 150 – 2000) using mass windows of 30 Da (*m/z* 150 – 300), 50 Da (*m/z* 300 – 600), 150 Da (*m/z* 600 – 900), and only one window from *m/z* 900 – 2000. For each mass spectrum, between 50 (*m/z* 900 – 2000) and 256 (*m/z* 150 – 210) scans were co-added with 100 – 300 ms ion accumulation time. The broadband mass spectra were recorded for all segments via adjusting the start *m/z* value according to the lower *m/z* value of the Q-isolation window, resulting in near-constant mass resolution across the mass range (SI_A: Figure S2).[3] Mass spectra were internally calibrated using a list of lipids (between *m/z* 87 and 1572, *n* = 473), resulting in mass accuracy < 0.1 ppm. Mass calibration was performed using Data-Analysis 5.0 (Bruker Daltonics, Billerica, U.S.A).

4.3. FT-ICR MS methods performace

In this study, we used two FT-ICR MS acquisition methods, highlighting their performance in terms of resolution and signal detection (Figure S2). Method A demonstrates an exponential decrease in resolution with increasing mass, while method B maintains a consistent resolution of approximately 106 across the entire mass range. Above *m/z* 850 , both methods show a drop in resolution, corresponding to a low-pollution region. In this region, the detected signals are more likely to originate from noise rather than analytes due to the low density of ion populations.

Method A uses broader *m/z* segments (100 Da) and a fixed IAT of 1 s, increasing to 1.5 s at higher *m/z*. While this approach captures a larger mass range per acquisition, it increases ion competition within the ICR cell. Space-charge effects, which occur when the ion population exceeds the cell's optimal capacity, lead to distorted peak shapes, reduced resolution, and a higher likelihood of signal suppression.[3] These effects are particularly evident in high-ion-density region, where ion density is higher, leading to a reduction in the detection of low-abundance species.

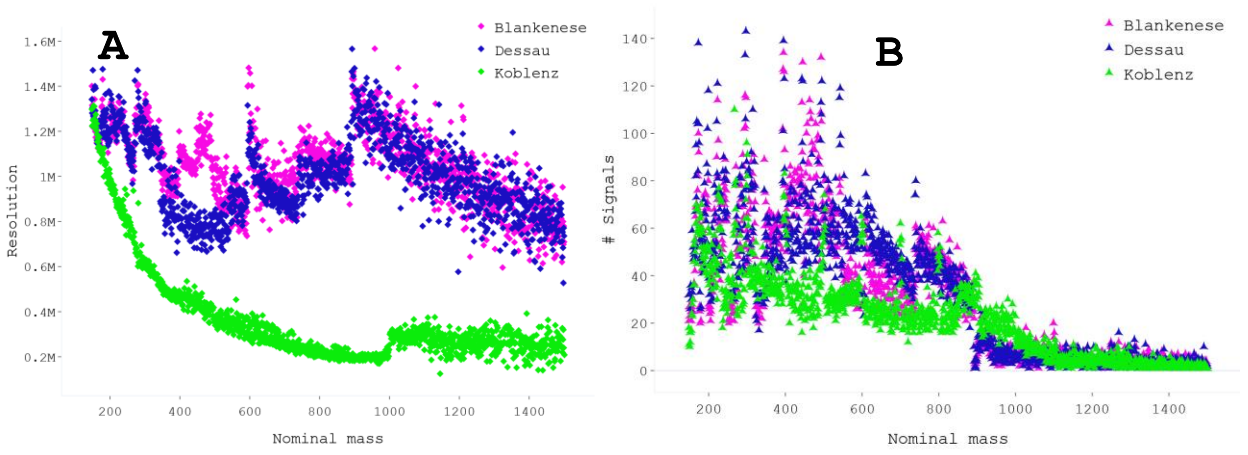

Figure S2 – A) Mean mass resolution and B) number of detected signals as a function of nominal mass for three samples measured using different methods. The bream liver sample from Koblenz (green) was analyzed with method A, while the Blankenese (pink) and Dessau (blue) samples were analyzed with method B.

Method B, by contrast, uses narrower *m/z* segments (50 Da) with dynamically decreasing IAT (300 ms in lower mass ranges, decreasing to 64 ms at higher masses). This strategy optimizes the total number of ions per segment, effectively reducing space-charge effects and maintaining resolution (Figure 1A). Additionally, co-added scans (256 in low-mass regions, decreasing to 64 at high masses) compensate for shorter IAT, improving the signal-to-noise ratio (S/N) and ensuring robust signal detection even at reduced accumulation times. This resulted in higher number of detected signals in samples measured by method B compared to method A, particularly in high-ion-density regions (Figure 1B). These findings demonstrate the importance of optimizing acquisition parameters for improved performance in complex sample analyses.

5. Software parameters for PFAS identification

Table S7 – Fundamental parameters utilized during data processing steps.

Software/Platform	Parameter	Value
MZmine3	Retention time	2.00-15.00 min.
	Mass detector	Centroid
	Noise level	$1x10^3$
	Min. #scans	5
	Intensity threshold	$5x10^3$

	Min. Highest intensity	$8x10^3$
	m/z tolerance	3 ppm
Pflow	Mass accuracy	10 ppm
	Isotope score	≥70
MassLynx: General parameters	PPM	15
	Minimum % RA	80
	DBE	0-10
	Electron state	even
	Criterion	i-FIT
	i-FIT peak count	4
MassLynx: Elemental parameters	C:0-50, H:0-100, F:0-100, N:0-4, O:0-10, S:0-4, P:0-1, Cl:0-3, Br:0-1	

6. Data processing

The stepwise data processing is described as follows:

1. *External Calibration:* The raw data acquired from FT-ICR MS underwent external calibration (lipids; *m/z* range: 87-1572 , n=473) and were used to create a reference library for PFAS identification.
2. *From* .raw to. CSV: HRMS data were processed in MZmine3[1,4] to generate a mass list, which was then extracted as a . CSV file.
3. *Feature screening:* HRMS data were matched against the reference library using a non-targeted approach with a mass accuracy threshold of 10 ppm by using *Pflow*[2].

3-4. *Selection of seed masses:* Candidates (HRMS) meeting specific criteria (lower mass cut off: *m/z* 230, mass defect: -0.15-0.15, peak intensity threshold: $1x10^3$, peak shape: visual screening) were selected as "seed masses" for generating homologs from the measured HRMS. This step was necessary to identify any homologs not initially detected by FT-ICR, which could have been linked to the instrument sensitivity or ion suppression for particular mass windows. Additionally, the region below 230 Da was heavily polluted with signals in both LC-qTOF and FT-ICR MS spectra, resulting in a high number of matched signals. However, upon closer examination, these signals were identified as noise without any defined chromatographic peaks. Since their intensities were slightly above our noise threshold, they were filtered out.

5. *Further Searching:* For the homolog search, we used a workflow created in the KNIME Analytics platform[5]. We calculated mass series ranging from 230 to 1000 Da from each "*seed mass"* and searched these against the "*Feature list"* with an accuracy of 10 ppm, using the parameters defined in step 4. Any additional homologs detected were added to the list for subsequent verification steps.
6. *Identification via Suspect List:* Preselected candidates, excluding those forming calculated homologs, were cross-referenced with the *PFAS:SL* (PFAS Suspect List) derived from the CompTox suspect list, which contains 12,034 substances.[6] To reduce the number of features, UHRMS data were used to calculate the isotopic patterns of these candidates. Candidates with a score of less than 70 were excluded from further analysis.
7. *Formula Generation for Unidentified Masses:* Masses that did not match any suspect compounds in the *PFAS:SL* were queried in the PubChem database, considering possible hydration or salt forms within 5 ppm. If the returned molecular formula did not contain fluorine (or the corresponding salt), those molecular formulas were excluded. For masses resulting in non-fluorinated molecular formulas, a new molecular formula was generated based on parameters shown in Table S7.

A schematic workflow for the stepwise data processing is shown in Figure S3.

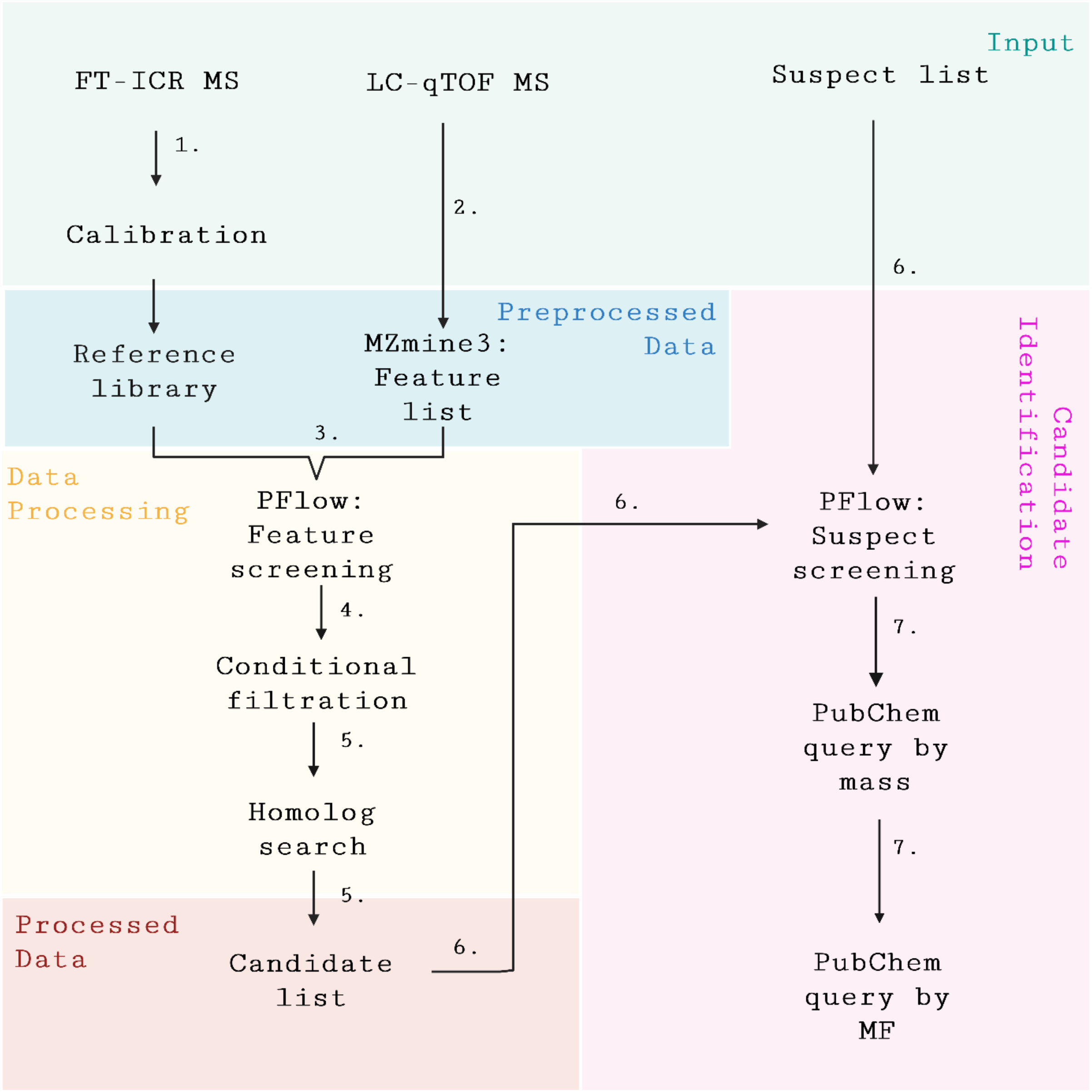

Figure S3 – Schematic representation of the data processing pipeline used in this study, divided into five color-coded sections: Input (green), Preprocessed Data (blue), Data Processing (yellow), Processed Data (red), and Candidate Identification (pink). Each step indicates what was either performed or produced at that specific stage, illustrating the progression from raw input data to the identification of potential candidates.

Table S8 – Calibrant and IS matching used in the data normalization process.

Compound	*m/z*	#C	Calibrant	Internal standard
PFPiA (C6/C6)	700.92262	12	PFDoDA	PFDA
PFPiA (C6/C8)	800.91624	14	PFTeDA	PFDA
PFPiA (C8/C8)	900.90985	16	PFTeDA	PFDA
6:1 PFESu	440.94717	6	PFHxS	PFOS
8:1 PFESu	540.94078	8	PFOS	PFOS
10:1 PFESu	640.9344	10	PFDS	PFOS
12:1 PFESu	740.92801	12	PFDS	PFOS
6:2/6:2 FTSAm	835.94937	12	FOSA	MeFOSAA
6:2/8:2 FTSAm	935.94299	14	FOSA	MeFOSAA
8:2/8:2 FTSAm	1035.9366	16	FOSA	MeFOSAA
[C13H10F17NO4S]	597.99863	8	FOSAA	MeFOSAA
[C14H10F19NO4S]	647.99544	9	FOSAA	MeFOSAA
[C15H10F21NO4S]	697.99224	10	FOSAA	MeFOSAA
[C15H10F21NO4S]	747.98905	11	FOSAA	MeFOSAA
[C16H10F23NO4S]	797.98586	12	FOSAA	MeFOSAA
[C17H10F25NO4S]	847.98266	13	FOSAA	MeFOSAA
FAP	444.94557	6	PFHxA	PFOA

Equation S1:

$$NF_y = \frac{I_{IS}}{\bar{I}_{IS,y}}$$

where:

NF_y = Normalization factor for year y

I_{IS} = Average peak intensity of the IS across all years

$I_{IS,y}$ = Measured peak intensity of the IS in year y

Equation S2:

$$I_{norm,i,y} = I_{i,y} \times NF_y$$

where:

$I_{norm,i,y}$ = Normalized peak intensity of compound i in year y

$I_{i,y}$ = Raw peak intensity of compound I in year y

NF_y = Normalization factor for year y

Equation S3:

$$I_{scaled,i,y} = \frac{I_{ISnorm,i,y} - \min(I_{norm,i})}{\max(\bar{I}_{Inorm,i}) - \min(I_{norm,i})} \times 100$$

where:

$I_{scaled,i,y}$ = Final scaled intensity (0-100%) for compound I in year y

$I_{norm,i,y}$ = Normalized peak intensity from Equation 2

max($I_{norm,i}$) = Maximum normalized intensity of compound I across all years

min($I_{norm,i}$) = Minimum normalized peak intensity of compound I across all years

Table S9 – Tentatively identified perfluorocarboxylic acids and their chemical identifiers.

Class	Proposed structure	Acronym	[M-H]⁻	*m/z*	Observed *m/z*	Mass accuracy (ppm)	Rt (min.)	CL
Perfluorocarboxylic acids	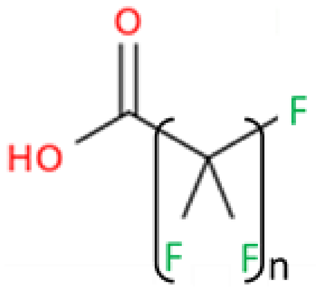	PFHxA	$[C_6F_{11}O_2]^-$	312.97281	246.9800	4.42	6.24	1a
		PFHpA	$[C_7F_{13}O_2]^-$	362.96962	296.9766	4.37	6.69	1a
		PFOA	$[C_8F_{15}O_2]^-$	412.96643	346.9728	5.49	7.19	1a
		PFNA	$[C_9F_{17}O_2]^-$	462.96323	396.9698	5.54	7.75	1a
		PFDA	$[C_{10}F_{19}O_2]^-$	512.96004	446.9674	2.05	8.44	1a
		PFUnDA	$[C_{11}F_{21}O_2]^-$	562.95684	496.9645	1.25	9.13	1a
		PFDoDA	$[C_{12}F_{23}O_2]^-$	612.955365	546.9618	0.24	9.78	1a
		PFTrDA	$[C_{13}F_{25}O_2]^-$	662.95046	596.9589	0.27	10.20	1a
		PFTeDA	$[C_{14}F_{27}O_2]^-$	712.94726	646.9560	0.71	10.35	1a
		PFPeDA	$[C_{15}F_{29}O_2]^-$	762.94407	696.9525	0.72	10.44	2c
		PFHxDA	$[C_{16}F_{31}O_2]^-$	812.94088	746.9448	5.62	10.51	2c

Molecular formula	IUPAC	SMILES	CAS RN	PubChem CID
$C_6HF_{11}O_2$	2,2,3,3,4,4,5,5,6,6,6-undecafluorohexanoic acid	C(=O)(C(C(C(C(C(F)(F)F)(F)F)(F)F)(F)F)(F)F)O	307-24-4	67542
$C_7HF_{13}O_2$	2,2,3,3,4,4,5,5,6,6,7,7,7-tridecafluoroheptanoic acid	C(=O)(C(C(C(C(C(C(F)(F)F)(F)F)(F)F)(F)F)(F)F)(F)F)O	375-85-9	67818
$C_8HF_{15}O_2$	2,2,3,3,4,4,5,5,6,6,7,7,8,8,8-pentadecafluorooctanoic acid	C(=O)(C(C(C(C(C(C(C(F)(F)F)(F)F)(F)F)(F)F)(F)F)(F)F)(F)F)O	335-67-1	9554
$C_9HF_{17}O_2$	2,2,3,3,4,4,5,5,6,6,7,7,8,8,9,9,9-heptadecafluorononanoic acid	C(=O)(C(C(C(C(C(C(C(C(F)(F)F)(F)F)(F)F)(F)F)(F)F)(F)F)(F)F)(F)F)O	375-95-1	67821
$C_{10}HF_{19}O_2$	2,2,3,3,4,4,5,5,6,6,7,7,8,8,9,9,10,10,10-nonadecafluorodecanoic acid	C(=O)(C(C(C(C(C(C(C(C(C(F)(F)F)(F)F)(F)F)(F)F)(F)F)(F)F)(F)F)(F)F)(F)F)O	335-76-2	9555
$C_{11}HF_{21}O_2$	2,2,3,3,4,4,5,5,6,6,7,7,8,8,9,9,10,10,11,11,11-<br>henicosafluoroundecanoic acid	C(=O)(C(C(C(C(C(C(C(C(C(C(F)(F)F)(F)F)(F)F)(F)F)(F)F)(F)F)(F)F)(F)F)(F)F)(F)<br>F)O	2058-94-8	77222
$C_{12}HF_{23}O_2$	2,2,3,3,4,4,5,5,6,6,7,7,8,8,9,9,10,10,11,11,12,12,12-<br>tricosafluorododecanoic acid	C(=O)(C(C(C(C(C(C(C(C(C(C(C(F)(F)F)(F)F)(F)F)(F)F)(F)F)(F)F)(F)F)(F)F)(F)F)(<br>F)F)(F)F)O	307-55-1	67545
$C_{13}HF_{25}O_2$	2,2,3,3,4,4,5,5,6,6,7,7,8,8,9,9,10,10,11,11,12,12,13,13,13-<br>pentacosafluorotridecanoic acid	C(=O)(C(C(C(C(C(C(C(C(C(C(C(C(F)(F)F)(F)F)(F)F)(F)F)(F)F)(F)F)(F)F)(F)F)(F)F)(F)<br>F)(F)F)(F)F)(F)F)O	72629-94-8	3018355
$C_{14}HF_{27}O_2$	2,2,3,3,4,4,5,5,6,6,7,7,8,8,9,9,10,10,11,11,12,12,13,13,14,14,14-<br>heptacosafluorotetradecanoic acid	C(=O)(C(C(C(C(C(C(C(C(C(C(C(C(C(F)(F)F)(F)F)(F)F)(F)F)(F)F)(F)F)(F)F)(F)F)(F)F)(<br>F)F)(F)F)(F)F)(F)F)(F)F)O	376-06-7	67822
$C_{15}HF_{29}O_2$	2,2,3,3,4,4,5,5,6,6,7,7,8,8,9,9,10,10,11,11,12,12,13,13,14,14,15,15,15<br>-nonacosafluoropentadecanoic acid	C(=O)(C(C(C(C(C(C(C(C(C(C(C(C(C(C(F)(F)F)(F)F)(F)F)(F)F)(F)F)(F)F)(F)F)(F)F)(F)<br>F)(F)F)(F)F)(F)F)(F)F)(F)F)(F)F)O	141074-63-7	12731198
$C_{16}HF_{31}O_2$	2,2,3,3,4,4,5,5,6,6,7,7,8,8,9,9,10,10,11,11,12,12,13,13,14,14,15,15,16<br>,16,16-hentriacontafluorohexadecanoic acid	C(=O)(C(C(C(C(C(C(C(C(C(C(C(C(C(C(C(F)(F)F)(F)F)(F)F)(F)F)(F)F)(F)F)(F)F)(F)F)(<br>F)F)(F)F)(F)F)(F)F)(F)F)(F)F)(F)F)(F)F)O	67905-19-5	106027

Figure S4a – Chromatograms of PFHxA, PFHpA, PFOA, and PFNA detected in bream liver sample (red color) alongside their respective standard solution (green color).

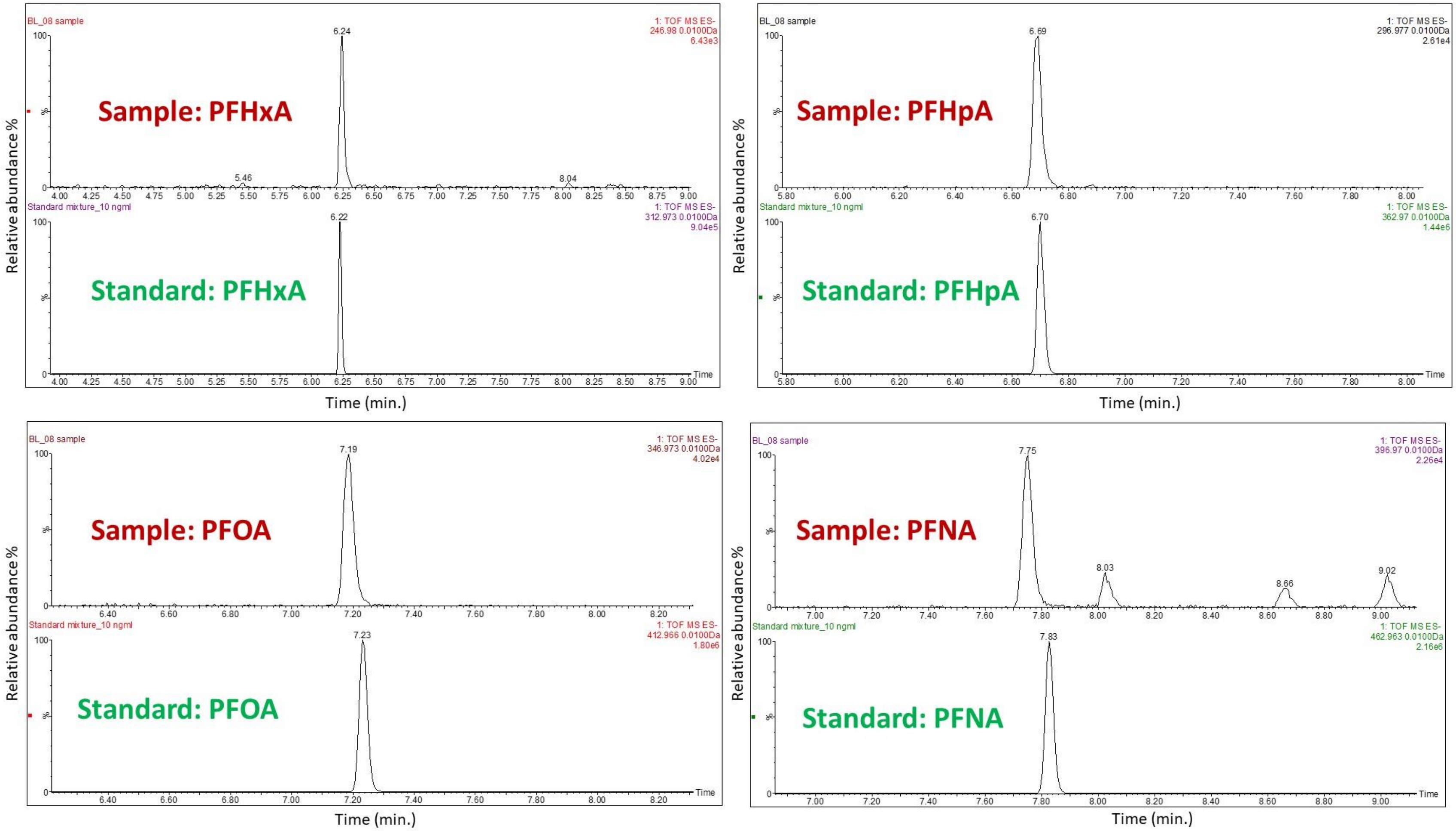

Figure S4b – Chromatograms of PFDA, PFUnDA, PFDoDA, and PFTrDA in bream liver sample (red color) and their corresponding standard solution (green color).

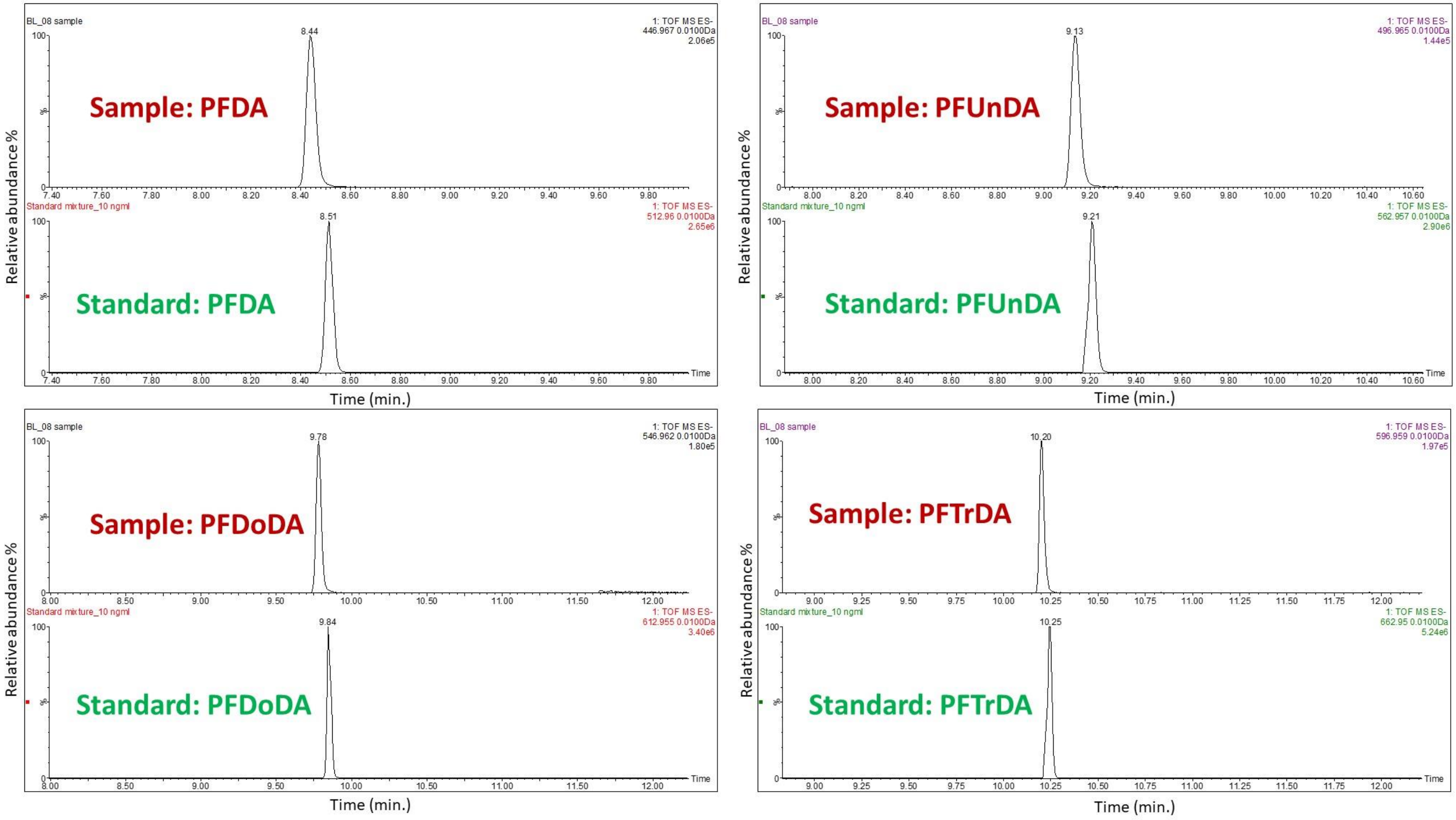

Figure S4c – Chromatograms of PFTeDA (red color), confirmed through comparison with the standard (green color), along with PFPeDA and PFHxDA, which were detected in the bream liver sample.

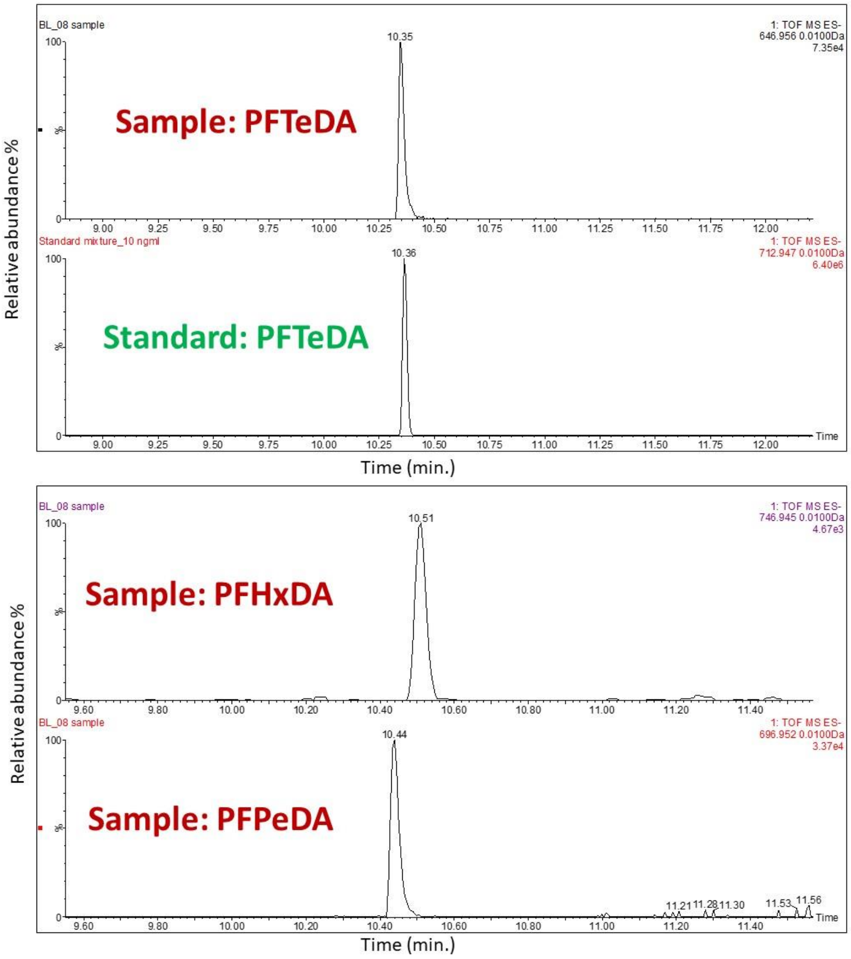

Figure S4d – Fragment ion mass spectra of PFDA detected in bream liver and confirmed with the standard. The spectrum highlights the molecular ion (in blue) alongside the common fragment ions (in pink) observed in both the sample and standard fragment ion mass spectra.

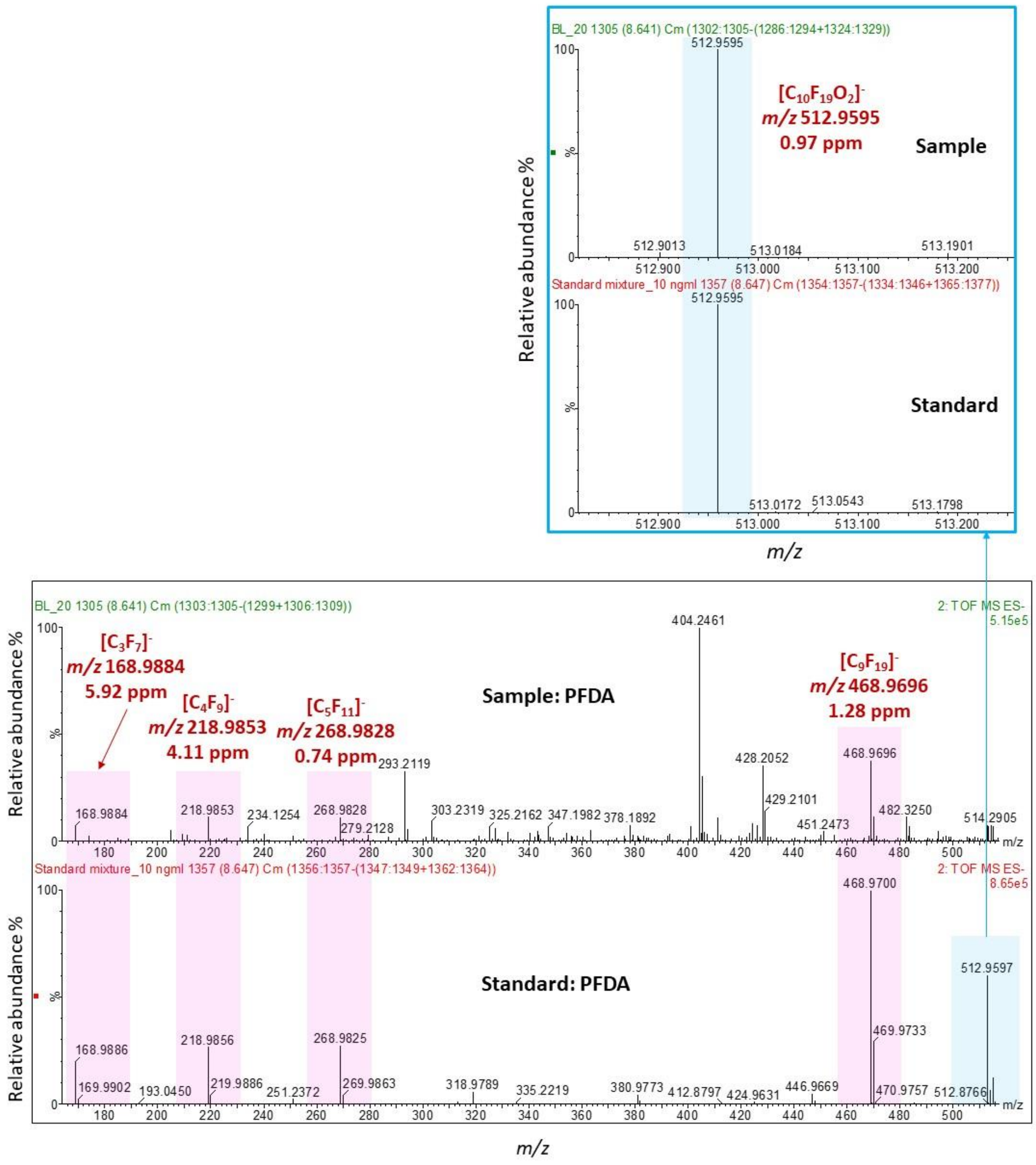

Table S10 – Tentatively identified perfluorosulfonic acids and their chemical identifiers.

Class	Proposed structure	Acronym	[M-H]$^-$	*m/z*	Observed *m/z*	Mass accuracy (ppm)	Rt (min.)	CL
Perfluorosulfonic acids	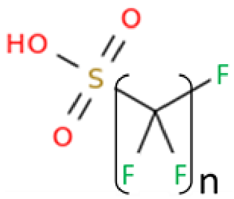	PFBS	$[C_4F_9O_3S]^-$	298.94299	298.9418	3.98	5.92	1a
		PFPeS	$[C_5F_{11}O_3S]^-$	348.93980	348.9381	4.87	6.39	2c
		PFHxS	$[C_6F_{13}O_3S]^-$	398.93661	398.9355	2.78	6.82	1a
		PFHpS	$[C_7F_{15}O_3S]^-$	448.93341	448.9320	3.14	7.33	2c
		PFOS	$[C_8F_{17}O_3S]^-$	498.93022	498.9326	4.77	7.97	1a
		PFNS	$[C_9F_{19}O_3S]^-$	548.92702	548.9265	0.94	8.59	2c
		PFDS	$[C_{10}F_{21}O_3S]^-$	598.92383	598.9236	0.38	9.26	1a
		PFUnDS	$[C_{11}F_{23}O_3S]^-$	648.92064	648.9210	0.55	9.89	2c
		PFDoDS	$[C_{12}F_{25}O_3S]^-$	698.91744	698.9175	0.09	10.23	2c
		PFTrDS	$[C_{13}F_{27}O_3S]^-$	748.91425	748.9147	0.60	10.3	2c
		PFTeDS	$[C_{14}F_{29}O_3S]^-$	798.91106	798.9095	1.95	10.4	2c

Molecular formula	IUPAC	SMILES	CAS RN	PubChem CID
$C_4HF_9O_3S$	1,1,2,2,3,3,4,4,4-nonafluorobutane-1-sulfonic acid	C(C(C(F)(F)S(=O)(=O)O)(F)F)(C(F)(F)F)(F)F	375-73-5	67815
$C_5HF_{11}O_3S$	1,1,2,2,3,3,4,4,5,5,5-undecafluoropentane-1-sulfonic acid	C(C(C(F)(F)F)(F)F)(C(C(F)(F)S(=O)(=O)O)(F)F)(F)F	2706-91-4	75922
$C_6HF_{13}O_3S$	1,1,2,2,3,3,4,4,5,5,6,6,6-tridecafluorohexane-1-sulfonic acid	C(C(C(C(F)(F)S(=O)(=O)O)(F)F)(F)F)(C(C(F)(F)F)(F)F)(F)F	355-46-4	67734
$C_7HF_{15}O_3S$	1,1,2,2,3,3,4,4,5,5,6,6,7,7,7-pentadecafluoroheptane-1-sulfonic acid	C(C(C(C(F)(F)F)(F)F)(F)F)(C(C(C(F)(F)S(=O)(=O)O)(F)F)(F)F)(F)F	375-92-8	67820
$C_8HF_{17}O_3S$	1,1,2,2,3,3,4,4,5,5,6,6,7,7,8,8,8-heptadecafluorooctane-1-sulfonic acid	C(C(C(C(C(F)(F)S(=O)(=O)O)(F)F)(F)F)(F)F)(C(C(C(F)(F)F)(F)F)(F)F)(F)F	1763-23-1	74483
$C_9HF_{19}O_3S$	1,1,2,2,3,3,4,4,5,5,6,6,7,7,8,8,9,9,9-nonadecafluorononane-1-sulfonic acid	C(C(C(C(C(F)(F)F)(F)F)(F)F)(F)F)(C(C(C(C(F)(F)S(=O)(=O)O)(F)F)(F)F)(F)F)(F)F	68259-12-1	86998
$C_{10}HF_{21}O_3S$	1,1,2,2,3,3,4,4,5,5,6,6,7,7,8,8,9,9,10,10,10-henicosafluorodecane-1-sulfonic acid	C(C(C(C(C(C(F)(F)S(=O)(=O)O)(F)F)(F)F)(F)F)(F)F)(C(C(C(C(F)(F)F)(F)F)(F)F)(F)F)(F)F	335-77-3	67636
$C_{11}HF_{23}O_3S$	1,1,2,2,3,3,4,4,5,5,6,6,7,7,8,8,9,9,10,10,11,11,11-tricosafluoroundecane-1-sulfonic acid	C(C(C(C(C(C(F)(F)F)(F)F)(F)F)(F)F)(F)F)(C(C(C(C(C(F)(F)S(=O)(=O)O)(F)F)(F)F)(F)F)(F)F)(F)F	749786-16-1	22141518
$C_{12}HF_{25}O_3S$	1,1,2,2,3,3,4,4,5,5,6,6,7,7,8,8,9,9,10,10,11,11,12,12,12-pentacosafluorododecane-1-sulfonic acid	C(C(C(C(C(C(C(F)(F)S(=O)(=O)O)(F)F)(F)F)(F)F)(F)F)(F)F)(C(C(C(C(C(F)(F)F)(F)F)(F)F)(F)F)(F)F)(F)F	79780-39-5	3018866
$C_{13}HF_{27}O_3S$	1,1,2,2,3,3,4,4,5,5,6,6,7,7,8,8,9,9,10,10,11,11,12,12,13,13,13-heptacosafluorotridecane-1-sulfonic acid	C(C(C(C(C(C(C(F)(F)F)(F)F)(F)F)(F)F)(F)F)(F)F)(C(C(C(C(C(C(F)(F)S(=O)(=O)O)(F)F)(F)F)(F)F)(F)F)(F)F)(F)F	791563-89-8	21964789
$C_{14}HF_{29}O_3S$	1,1,2,2,3,3,4,4,5,5,6,6,7,7,8,8,9,9,10,10,11,11,12,12,13,13,14,14,14-nonacosafluorotetradecane-1-sulfonic acid	C(C(C(C(C(C(C(C(F)(F)S(=O)(=O)O)(F)F)(F)F)(F)F)(F)F)(F)F)(F)F)(C(C(C(C(C(C(F)(F)F)(F)F)(F)F)(F)F)(F)F)(F)F)(F)F	1379460-39-5	21964855

Figure S5a – Chromatograms of PFBS, PFHxS, PFOS, and PFDS detected in bream liver samples (red) and compared to their respective standards (green).

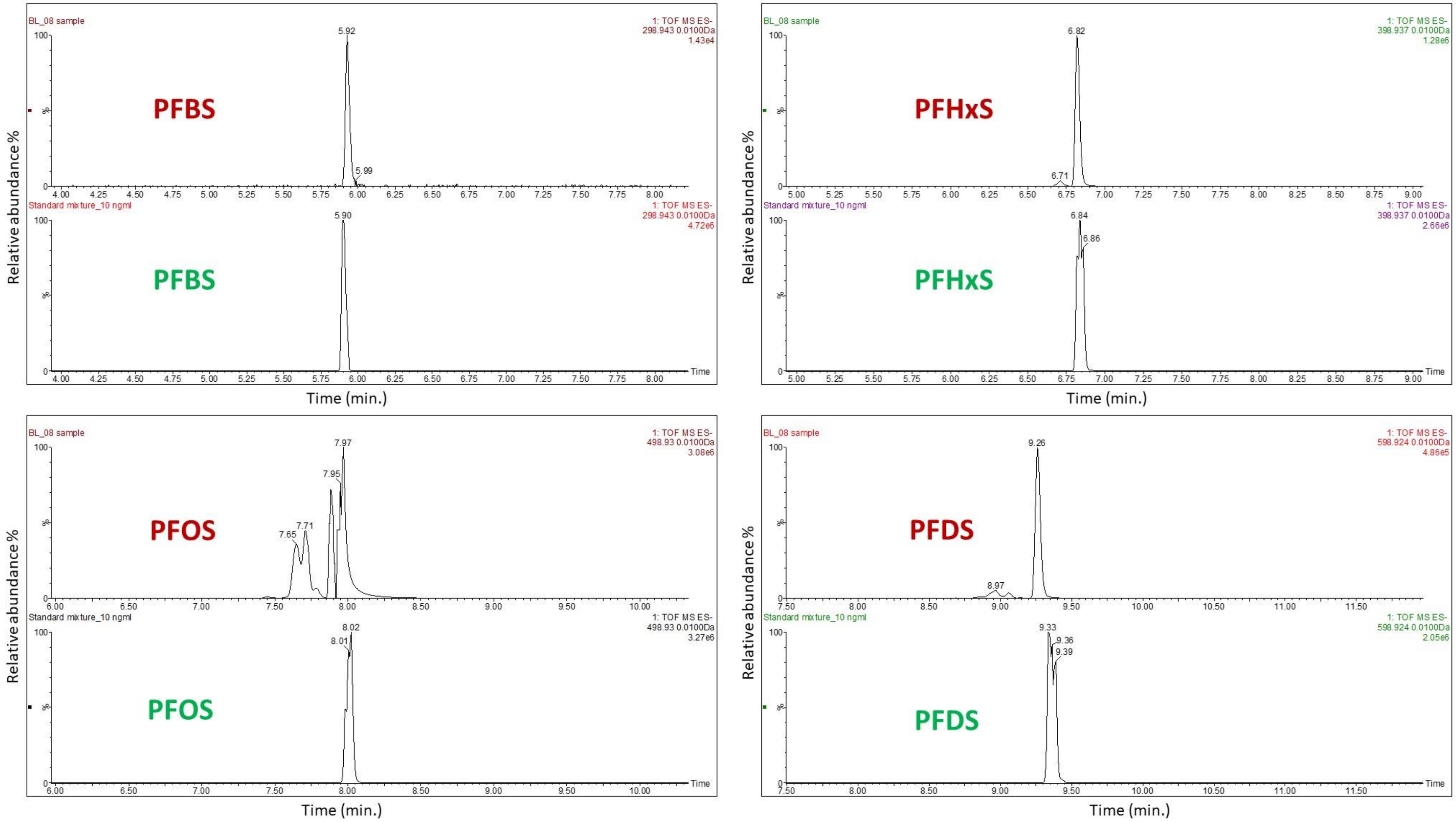

Figure S5b – Chromatograms of seven PFSA homologs, namely PFPeS, PFHpS, PFNS, PFUnDS, PFDoDS, PFTrDS, and PFTeDS, detected in bream liver sample.

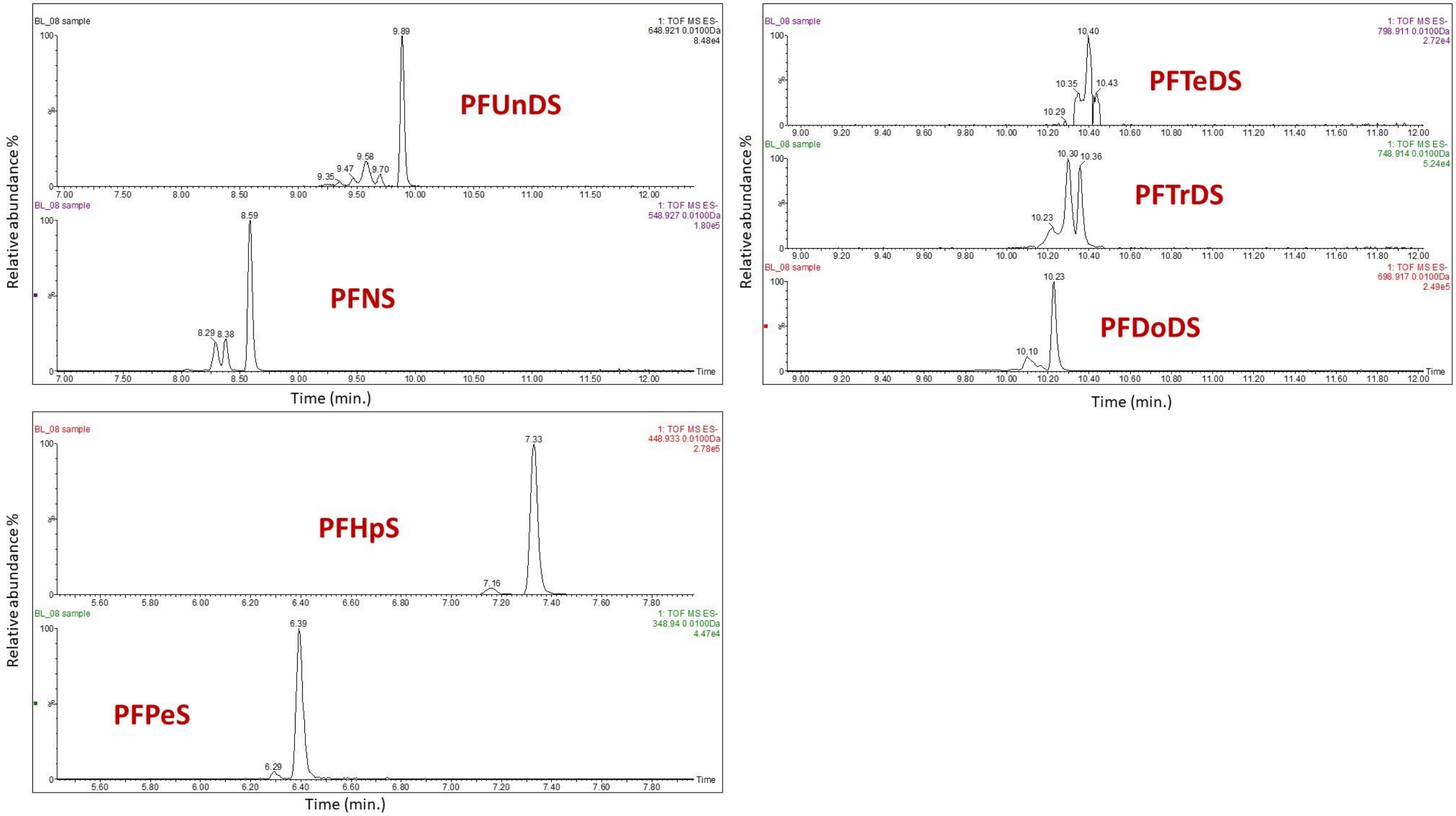

Figure S5c – Ion fragment mass spectrum spectrum of PFOS detected in the bream liver sample compared to the standard. The black-outlined mass spectrum represents the full scan, highlighting the common fragments observed in the spectra. The blue-outlined spectrum is a cropped section between *m/z* 225 and 330, focusing on the specific fragments of PFOS found in both the bream liver sample and the standard, confirming the match between them.

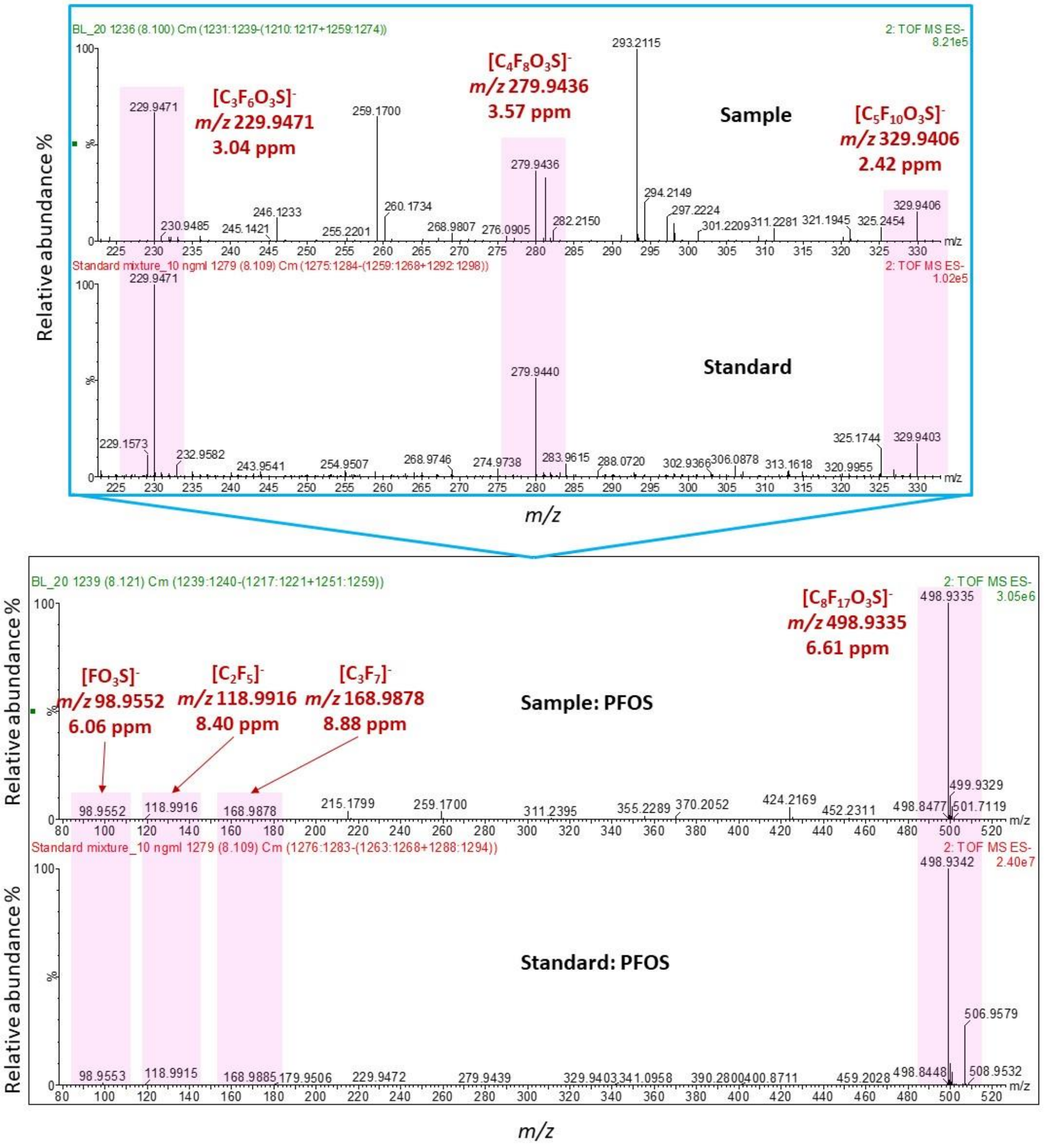

Table S11 – Tentatively identified perfluorinated sulfonamides and their chemical identifiers.

Class	Proposed structure	Acronym	$[M-H]^-$	*m/z*	Observed *m/z*	Mass accuracy (ppm)	Rt (min.)	CL
Perfluorinated sulfonamides	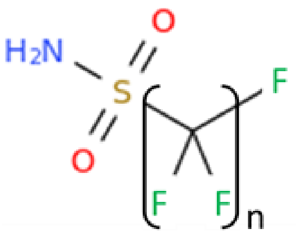	FBSA	$[C_4HF_9NO_2S]^-$	297.95898	297.9578	3.96	6.41	3c
		FPeSA	$[C_5HF_{11}NO_2S]^-$	347.95578	347.9546	3.39	6.99	3c
		FHxSA	$[C_6HF_{13}NO_2S]^-$	397.95259	397.9514	3.00	7.63	3c
		FHpSA	$[C_7HF_{15}NO_2S]^-$	447.94940	447.9485	2.01	8.37	3c
		FOSA	$[C_8HF_{17}NO_2S]^-$	497.94620	497.9460	0.40	9.16	1a

Class	Molecular formula	IUPAC	SMILES	CAS RN	PubChem CID
Perfluorinated sulfonamides	C4H2F9NO2S	1,1,2,2,3,3,4,4,4-nonafluorobutane-1-sulfonamide	C(C(C(F)(F)S(=O)(=O)N)(F)F)(C(F)(F)F)(F)F	30334-69-1	10958205
	C5H2F11NO2S	1,1,2,2,3,3,4,4,5,5,5-undecafluoropentane-1-sulfonamide	C(C(C(F)(F)F)(F)F)(C(C(F)(F)S(=O)(=O)N)(F)F)(F)F	82765-76-2	87793813
	C6H2F13NO2S	1,1,2,2,3,3,4,4,5,5,6,6,6-tridecafluorohexane-1-sulfonamide	C(C(C(C(F)(F)S(=O)(=O)N)(F)F)(F)F)(C(C(F)(F)F)(F)F)(F)F	41997-13-1	11603678
	C7H2F15NO2S	1,1,2,2,3,3,4,4,5,5,6,6,7,7,7-pentadecafluoroheptane-1-sulfonamide	C(C(C(C(F)(F)F)(F)F)(F)F)(C(C(C(F)(F)S(=O)(=O)N)(F)F)(F)F)(F)F	82765-77-3	11525204
	C8H2F17NO2S	1,1,2,2,3,3,4,4,5,5,6,6,7,7,8,8,8-heptadecafluorooctane-1-sulfonamide	C(C(C(C(C(F)(F)S(=O)(=O)N)(F)F)(F)F)(F)F)(C(C(C(F)(F)F)(F)F)(F)F)(F)F	754-91-6	69785

Figure S6a – Chromatograms of five homologs, namely FBSA, FPeSA, FHxSA, FHpSA, and FOSA, detected in the bream liver sample. FOSA was confirmed through comparison with the standard, as shown in the chromatogram (green font).

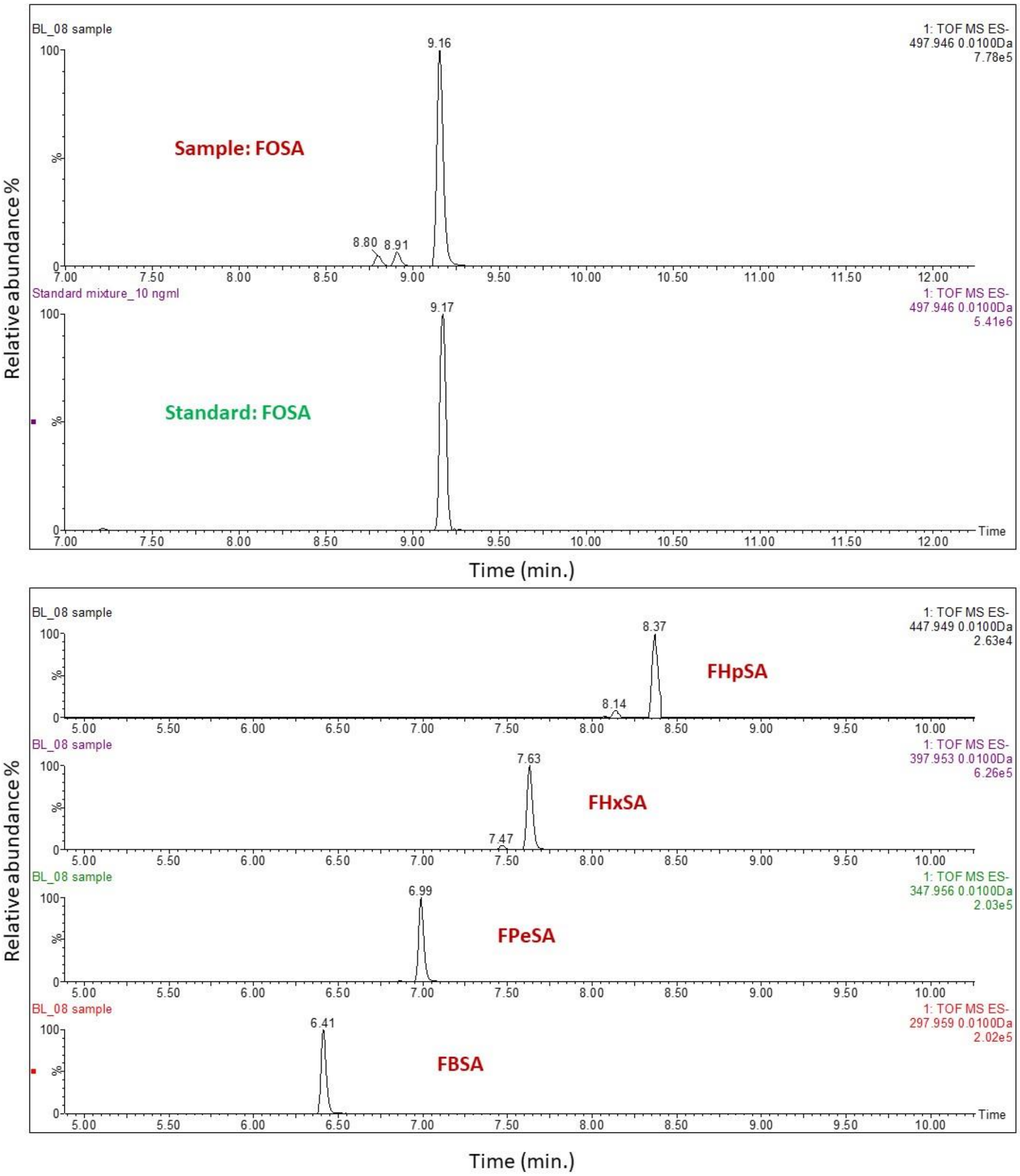

Figure S6b – Fragment ion mass spectrum of FOSA, displaying the molecular ion and the common fragments observed in both the measured bream liver sample and the corresponding standard.

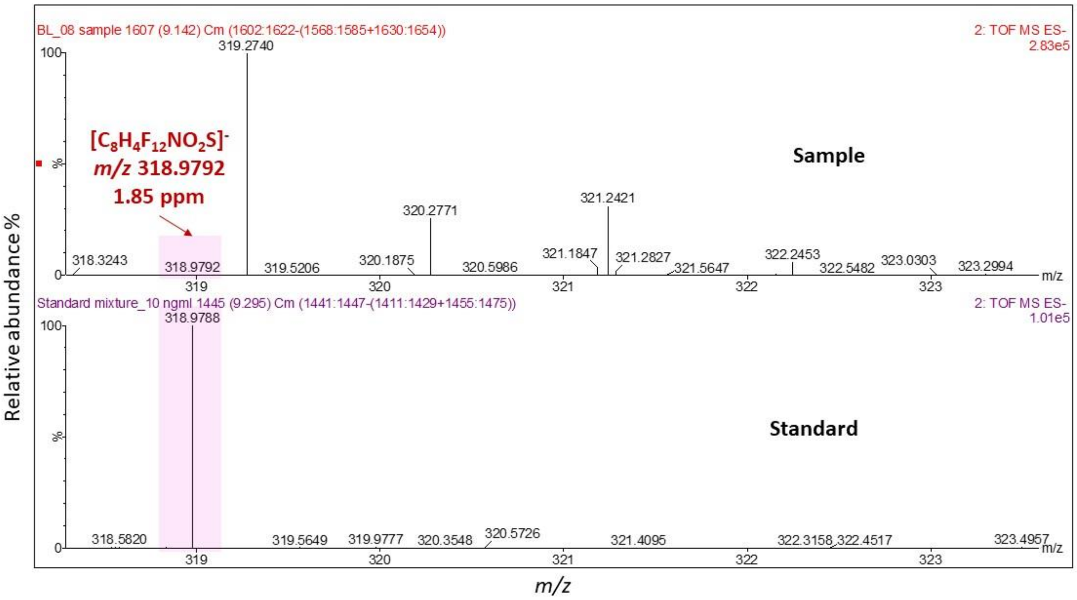

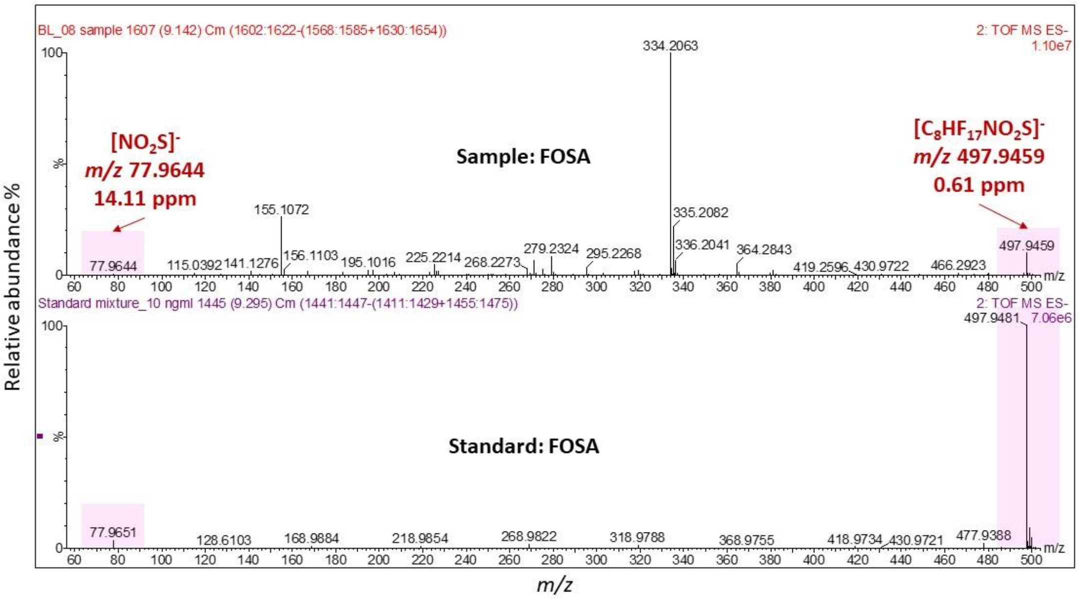

Figure S6c – Chromatograms of FBSA, FPeSA, FHxSA, and FHpSA detected in the bream liver sample, along with a zoomed-in view of the fragment ion mass spectrum. The spectrum highlights the common fragment observed in fragment ion mass spectra.

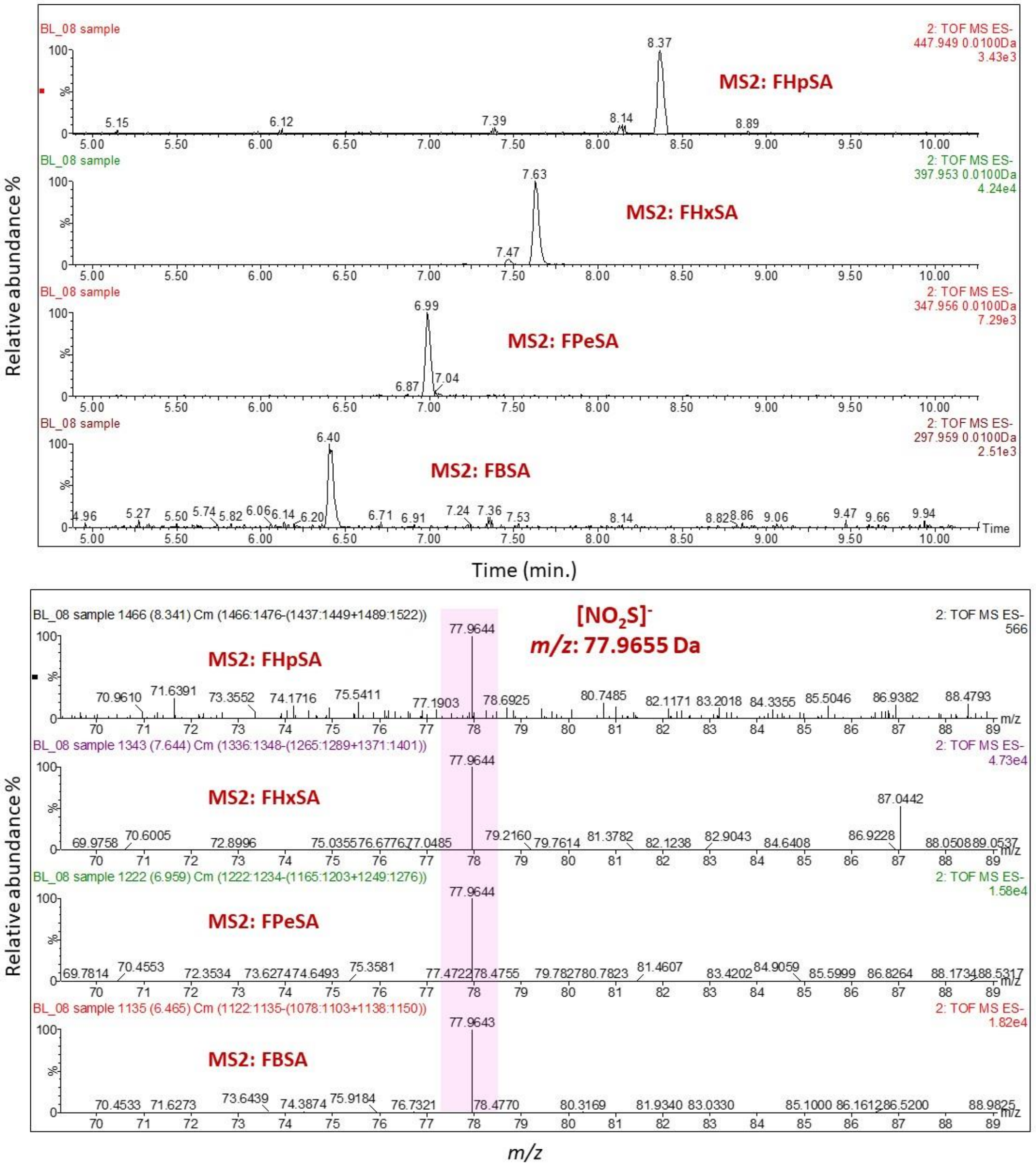

Table S12 – Tentatively identified perfluorooctane sulfonamido acetic acids and their chemical identifiers.

Class	Proposed structure	Acronym	$[M-H]^-$	*m/z*	Observed *m/z*	Mass accuracy (ppm)	Rt (min.)	CL
Perfluorooctane sulfonamido acetic acids	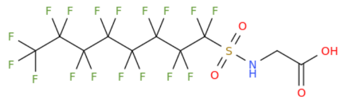	FOSAA	$[C_{10}H_3F_{17}NO_4S]^-$	555.95168	555.9508	1.58	8.30	1a
	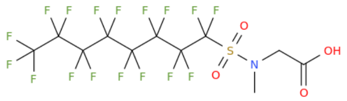	MeFOSAA	$[C_{11}H_5F_{17}NO_4S]^-$	569.96733	569.9664	1.63	8.66	1a
	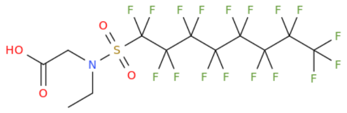	EtFOSAA	$[C_{12}H_7F_{17}NO_4S]^-$	583.98298	583.9825	0.82	9.03	1a

Molecular formula	IUPAC	SMILES	CAS RN	PubChem CID
$C_{10}H_4F_{17}NO_4S$	2-(1,1,2,2,3,3,4,4,5,5,6,6,7,7,8,8,8-heptadecafluorooctylsulfonylamino)acetic acid	C(C(=O)O)NS(=O)(=O)C(C(C(C(C(C(C(C(F)(F)F)(F)F)(F)F)(F)F)(F)F)(F)F)(F)F)(F)F	2806-24-8	10507011
$C_{11}H_6F_{17}NO_4S$	2-[1,1,2,2,3,3,4,4,5,5,6,6,7,7,8,8,8-heptadecafluorooctylsulfonyl(methyl)amino]acetic acid	CN(CC(=O)O)S(=O)(=O)C(C(C(C(C(C(C(C(F)(F)F)(F)F)(F)F)(F)F)(F)F)(F)F)(F)F)(F)F	2355-31-9	22286931
$C_{12}H_8F_{17}NO_4S$	2-[ethyl(1,1,2,2,3,3,4,4,5,5,6,6,7,7,8,8,8-heptadecafluorooctylsulfonyl)amino]acetic acid	CCN(CC(=O)O)S(=O)(=O)C(C(C(C(C(C(C(C(F)(F)F)(F)F)(F)F)(F)F)(F)F)(F)F)(F)F)(F)F	2991-50-6	18134

Figure S7a – Chromatograms of FOSAA, MeFOSAA, and EtFOSAA measured in the bream liver sample and compared with the corresponding analytical standard.

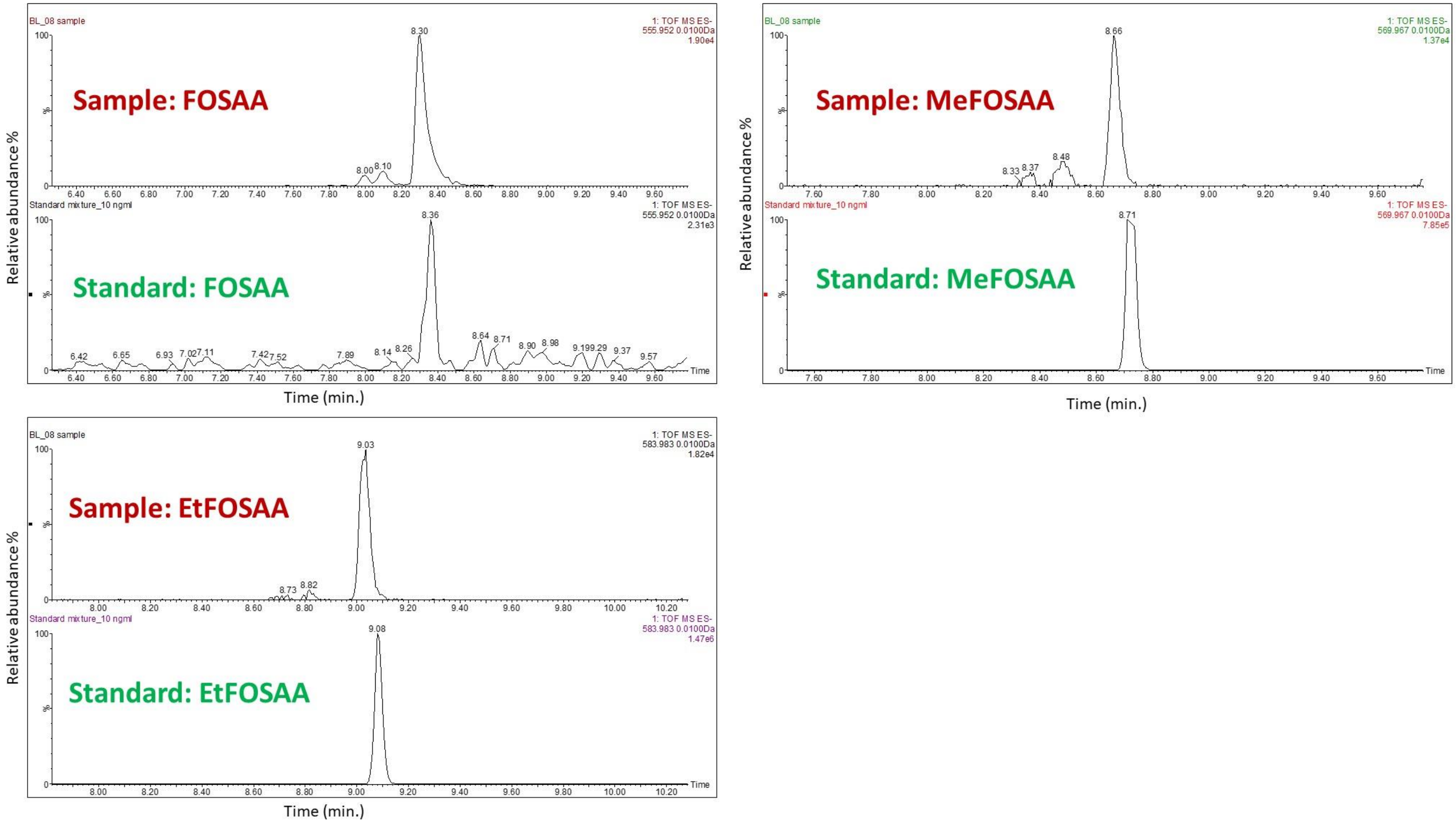

Table S13 – Tentatively identified fluorotelomer sulfonic acids and their chemical identifiers.

Class	Proposed structure	Acronym	$[M-H]^-$	*m/z*	Observed *m/z*	Mass accuracy (ppm)	Rt (min.)	CL
Fluorotelomer sulfonic acids	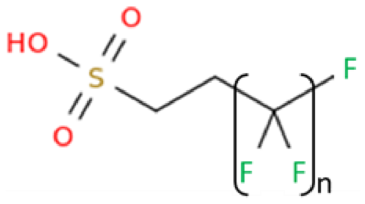	6:2 FTSA	$[C_8H_4F_{13}O_3S]^-$	426.96791	426.9665	3.30	7.08	1a
		8:2 FTSA	$[C_{10}H_4F_{17}O_3S]^-$	526.96152	526.9601	2.69	8.32	1a
		10:2 FTSA	$[C_{12}H_4F_{21}O_3S]^-$	626.95513	626.9559	1.23	9.68	2c
		12:2 FTSA	$[C_{14}H_4F_{25}O_3S]^-$	726.94874	726.9496	1.18	10.33	2c
		14:2 FTSA	$[C_{16}H_4F_{29}O_3S]^-$	826.94266	826.9430	0.41	10.50	2c

Class	Molecular formula	IUPAC	SMILES	CAS RN	PubChem CID
Fluorotelomer sulfonic acids	$C_8H_5F_{13}O_3S$	3,3,4,4,5,5,6,6,7,7,8,8,8-tridecafluorooctane-1-sulfonic acid	C(CS(=O)(=O)O)C(C(C(C(C(C(F)(F)F)(F)F)(F)F)(F)F)(F)F)(F)F	27619-97-2	119688
	$C_{10}H_5F_{17}O_3S$	3,3,4,4,5,5,6,6,7,7,8,8,9,9,10,10,10-heptadecafluorodecane-1-sulfonic acid	C(CS(=O)(=O)O)C(C(C(C(C(C(C(C(F)(F)F)(F)F)(F)F)(F)F)(F)F)(F)F)(F)F)(F)F	39108-34-4	3016044
	$C_{12}H_5F_{21}O_3S$	3,3,4,4,5,5,6,6,7,7,8,8,9,9,10,10,11,11,12,12,12-henicosafluorododecane-1-sulfonic acid	C(CS(=O)(=O)O)C(C(C(C(C(C(C(C(C(C(F)(F)F)(F)F)(F)F)(F)F)(F)F)(F)F)(F)F)(F)F)(F)F)(F)F	120226-60-0	23136180
	$C_{14}H_5F_{25}O_3S$	3,3,4,4,5,5,6,6,7,7,8,8,9,9,10,10,11,11,12,12,13,13,14,14,14-pentacosafluorotetradecane-1-sulfonic acid	C(CS(=O)(=O)O)C(C(C(C(C(C(C(C(C(C(C(C(F)(F)F)(F)F)(F)F)(F)F)(F)F)(F)F)(F)F)(F)F)(F)F)(F)F)(F)F)(F)F	149246-64-0	23136179
	$C_{16}H_5F_{29}O_3S$	3,3,4,4,5,5,6,6,7,7,8,8,9,9,10,10,11,11,12,12,13,13,14,14,15,15,16,16,16-nonacosafluorohexadecane-1-sulfonic acid	C(CS(=O)(=O)O)C(C(C(C(C(C(C(C(C(C(C(C(C(C(F)(F)F)(F)F)(F)F)(F)F)(F)F)(F)F)(F)F)(F)F)(F)F)(F)F)(F)F)(F)F)(F)F)(F)F	1377603-17-2	101318432

Figure S8a – Chromatograms of five homologs belonging to the n:2 FTSA group detected in the bream liver sample. The chromatograms of 6:2 FTSA and 8:2 FTSA show a comparison between the sample and the corresponding analytical standard.

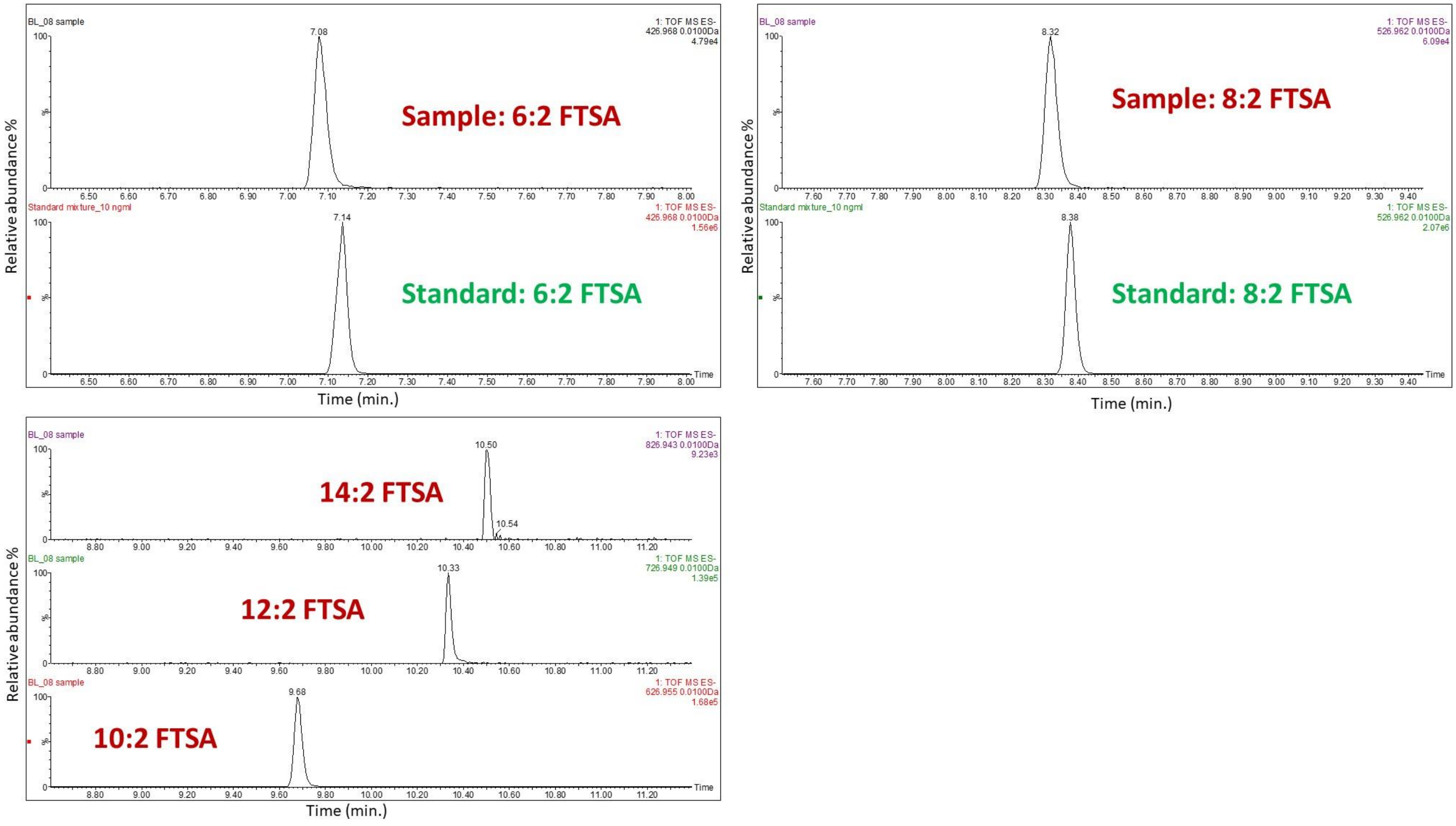

Figure S8b – Fragment ion mass spectrum showing the common fragments between the bream liver sample and the analytical standard. The molecular ion is highlighted in pink, while the blue and green colors represent the common fragments detected in both the sample and the standard. The upper portion of the spectrum is a zoomed-in view, showcasing these fragments in greater detail.

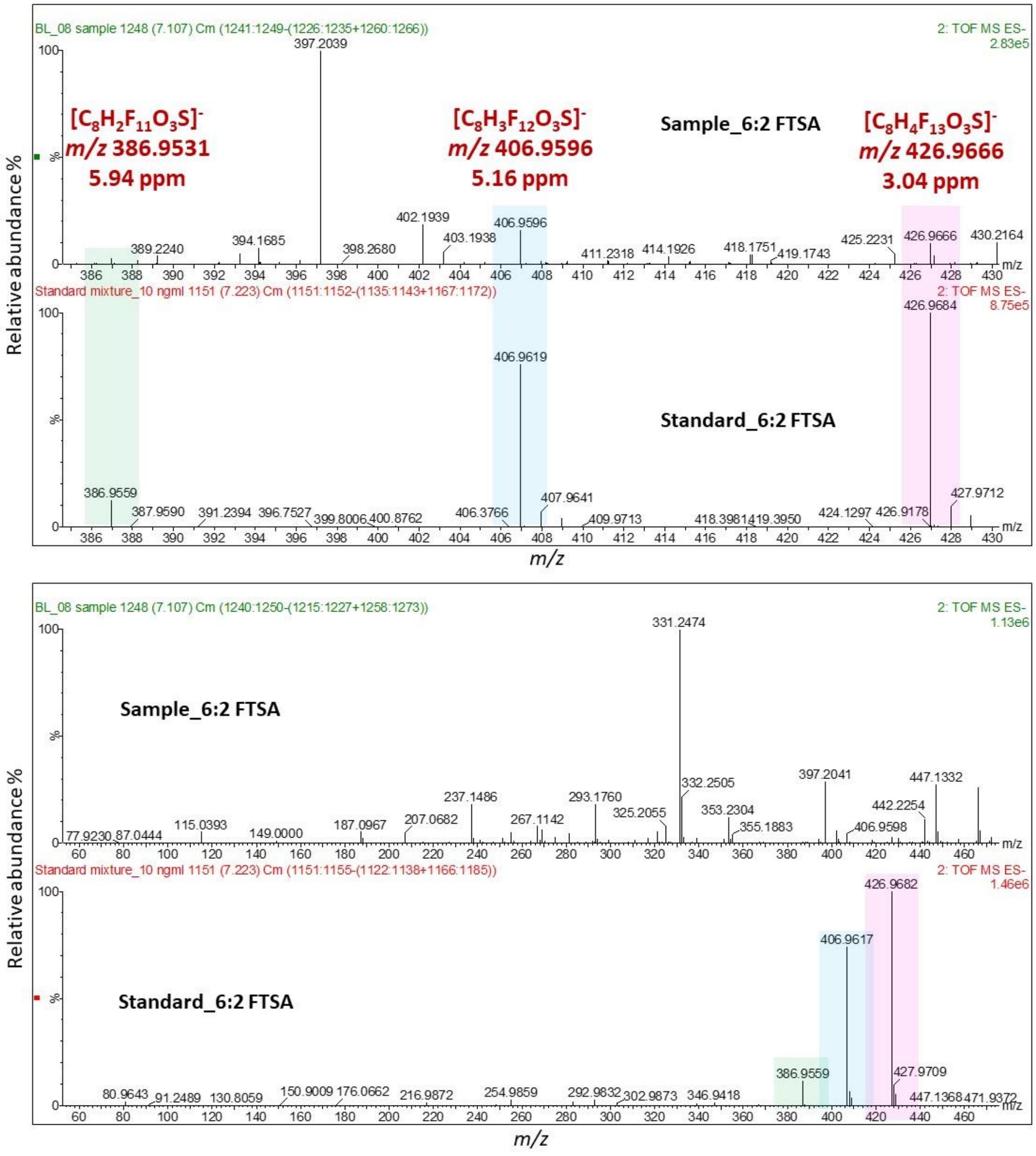

Figure S8c – Fragment ion mass spectrum showing the common fragments between the bream liver sample and the 8:2 FTSA analytical standard. The molecular ion is highlighted in pink, with blue and green colors indicating the common fragments detected in both the sample and the standard. The upper section of the spectrum provides a zoomed-in view, emphasizing these fragments for clearer comparison.

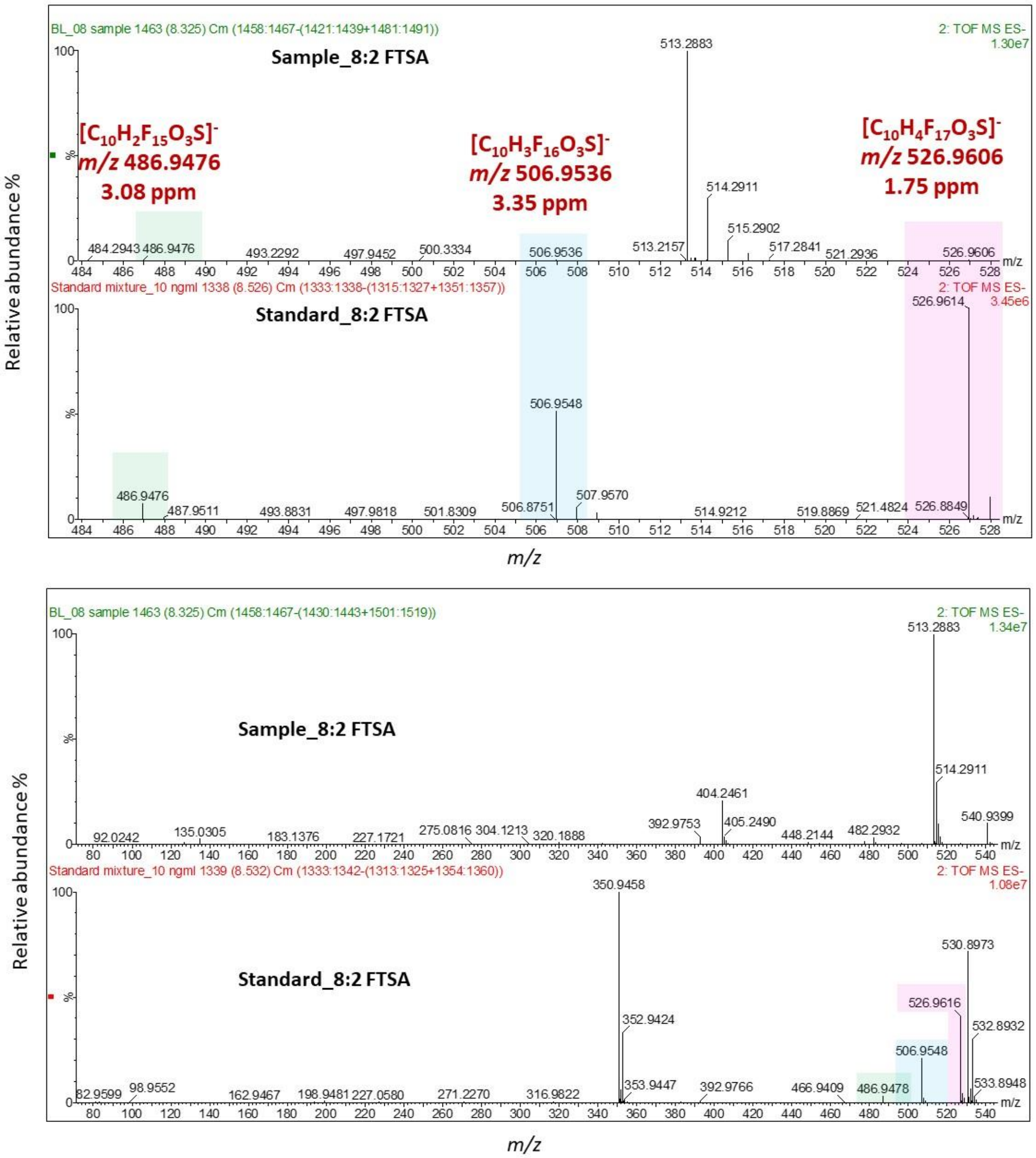

Table S14 – Tentatively identified perfluoroalkyl phosphinic acids and their chemical identifiers.

Class	Proposed structure	Acronym	$[M-H]^-$	*m/z*	Observed *m/z*	Mass accuracy (ppm)	Rt (min.)	CL
Perfluoroalkyl phosphinic acids	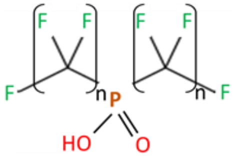	PFPiA (C6/C6)	$[C_{12}F_{26}O_2P]^-$	700.92262	700.9228	0.26	10.15	1a
		PFPiA (C6/C8)	$[C_{14}F_{30}O_2P]^-$	800.91624	800.9165	0.32	10.40	1a
		PFPiA (C8/C8)	$[C_{16}F_{34}O_2P]^-$	900.90985	900.9097	0.17	10.52	1a

Class	Molecular formula	IUPAC	SMILES	CAS RN	PubChem CID
Perfluoroalkyl phosphinic acids	$C_{12}HF_{26}O_2P$	bis(1,1,2,2,3,3,4,4,5,5,6,6,6-tridecafluorohexyl)phosphinic acid	C(C(C(C(F)(F)P(=O)(C(C(C(C(C(C(F)(F)F)(F)F)(F)F)(F)F)(F)F)(F)F)O)(F)F)(F)F)(C(C(F)(F)F)(F)F)(F)F	40143-77-9	71363384
	$C_{14}HF_{30}O_2P$	bis(1,1,2,2,3,3,4,4,5,5,6,6,7,7,7-pentadecafluoroheptyl)phosphinic acid	C(C(C(C(F)(F)F)(F)F)(F)F)(C(C(C(F)(F)P(=O)(C(C(C(C(C(C(C(F)(F)F)(F)F)(F)F)(F)F)(F)F)(F)F)(F)F)O)(F)F)(F)F)(F)F	158986-67-5	15930819
	$C_{16}HF_{34}O_2P$	bis(1,1,2,2,3,3,4,4,5,5,6,6,7,7,8,8,8-heptadecafluorooctyl)phosphinic acid	C(C(C(C(C(F)(F)P(=O)(C(C(C(C(C(C(C(C(F)(F)F)(F)F)(F)F)(F)F)(F)F)(F)F)(F)F)(F)F)O)(F)F)(F)F)(F)F)(C(C(C(F)(F)F)(F)F)(F)F)(F)F	40143-79-1	13528039

Figure S9a – Chromatograms of PFPiA (C6/C6), PFPiA (C6/C8), and PFPiA (C8/C8) detected in bream liver samples (in red) and confirmed with the corresponding standards (in green).

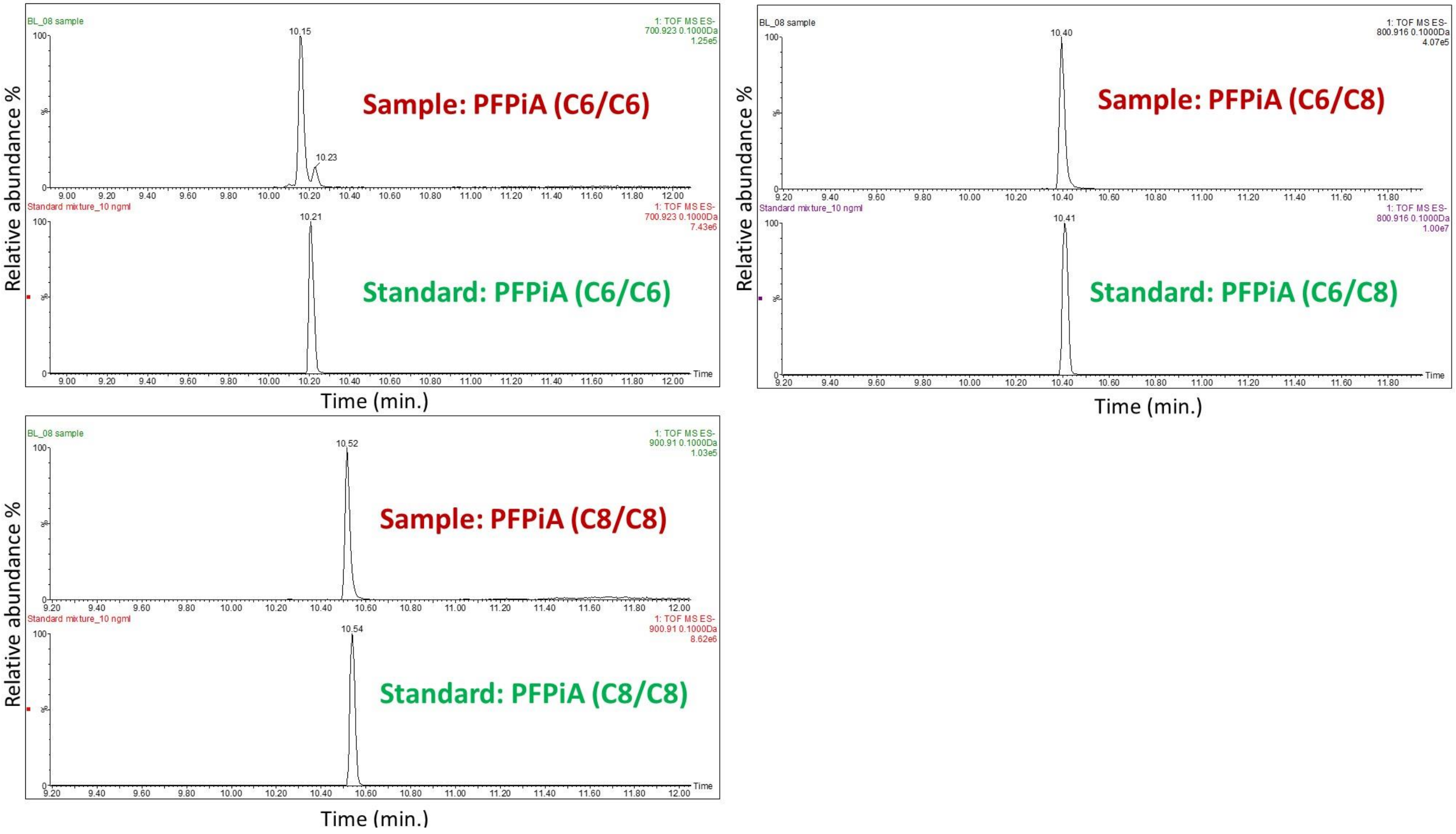

Figure S9b – Zoomed-in view of the fragment ion mass spectrum of PFPiA (C6/C6) for both the standard and the sample. The spectrum highlights the molecular ion (pink color), with the common fragment further emphasized in the cropped mass spectrum (blue outline), showcasing the consistency between the bream liver sample and the standard.

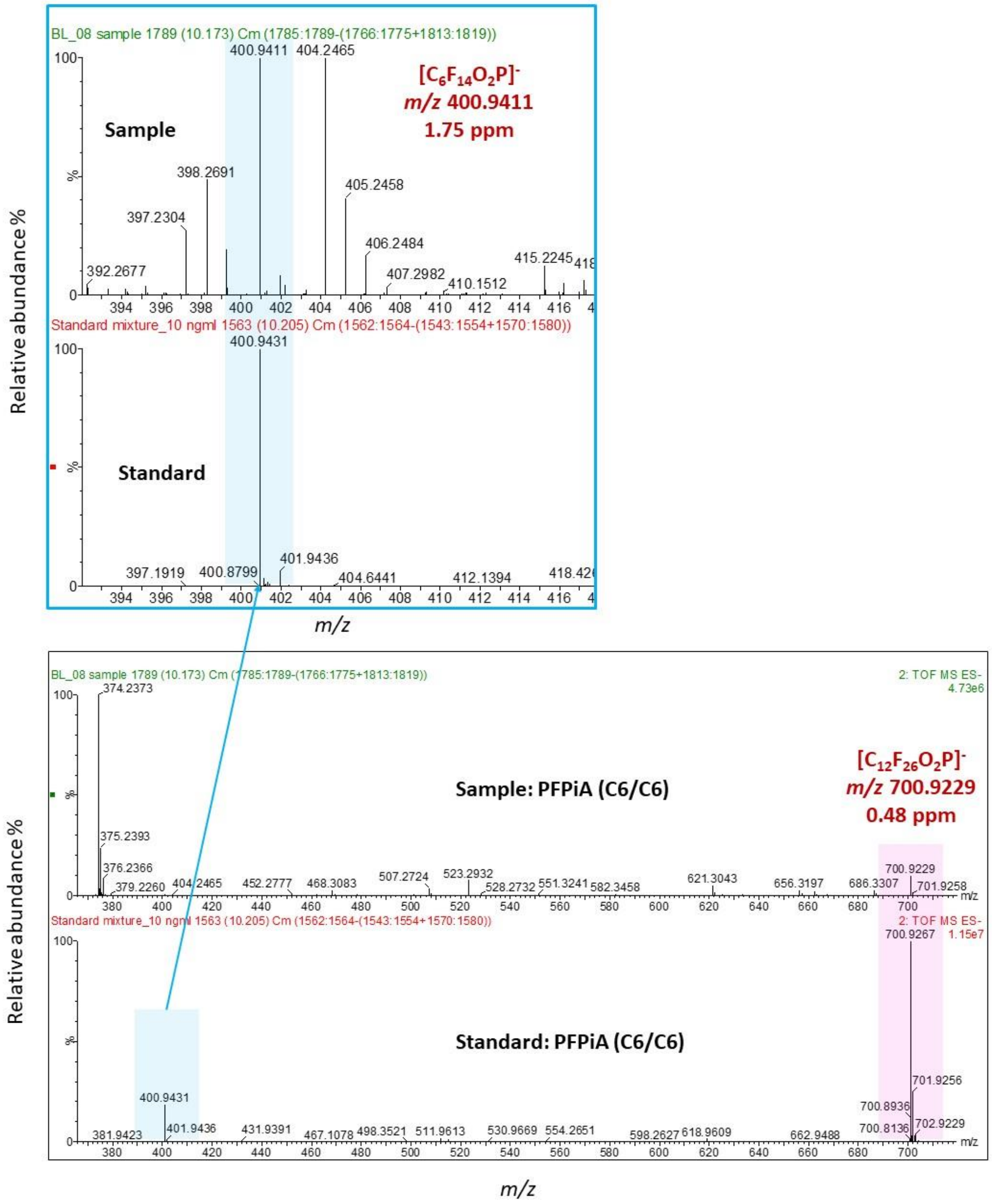

Figure S9c – Fragment ion mass spectrum of PFPiA (C6/C8) for both the bream liver sample and the standard. The molecular ion is highlighted in pink, while the common fragments are marked in green and blue, indicating their detection in both the sample and the standard. The green- and blue-outlined spectra show the cropped mass spectrum, emphasizing the detected fragments in detail.

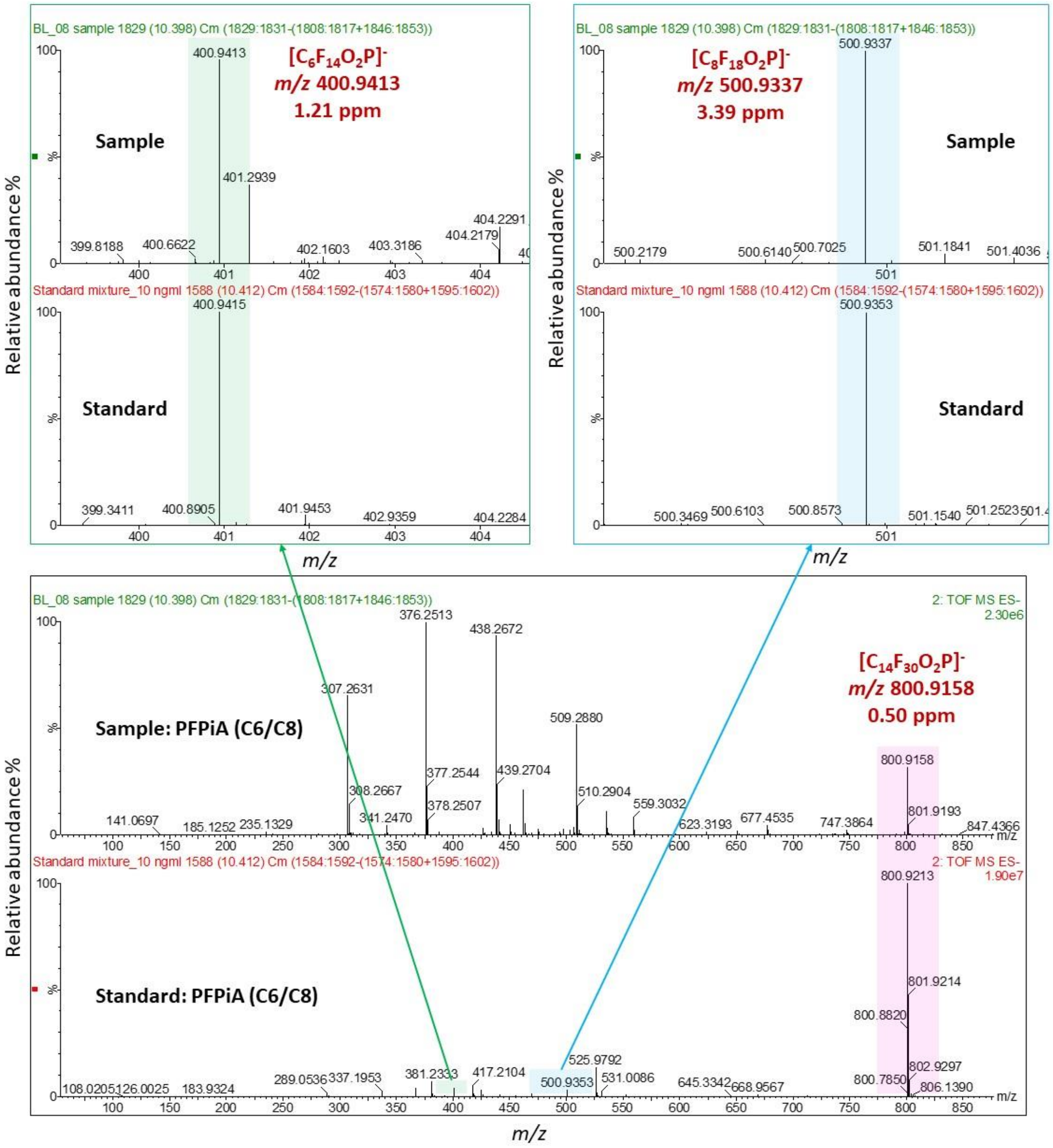

Figure S9d – Fragment ion mass spectrum of PFPiA (C8/C8) detected in both the bream liver sample and the standard. The molecular ion is shown in pink, while the common fragment observed in both the sample and the standard is highlighted in blue. The blue-outlined spectrum shows a zoomed-in view of the mass range between *m/z* 500 and 502, focusing on the detected fragment.

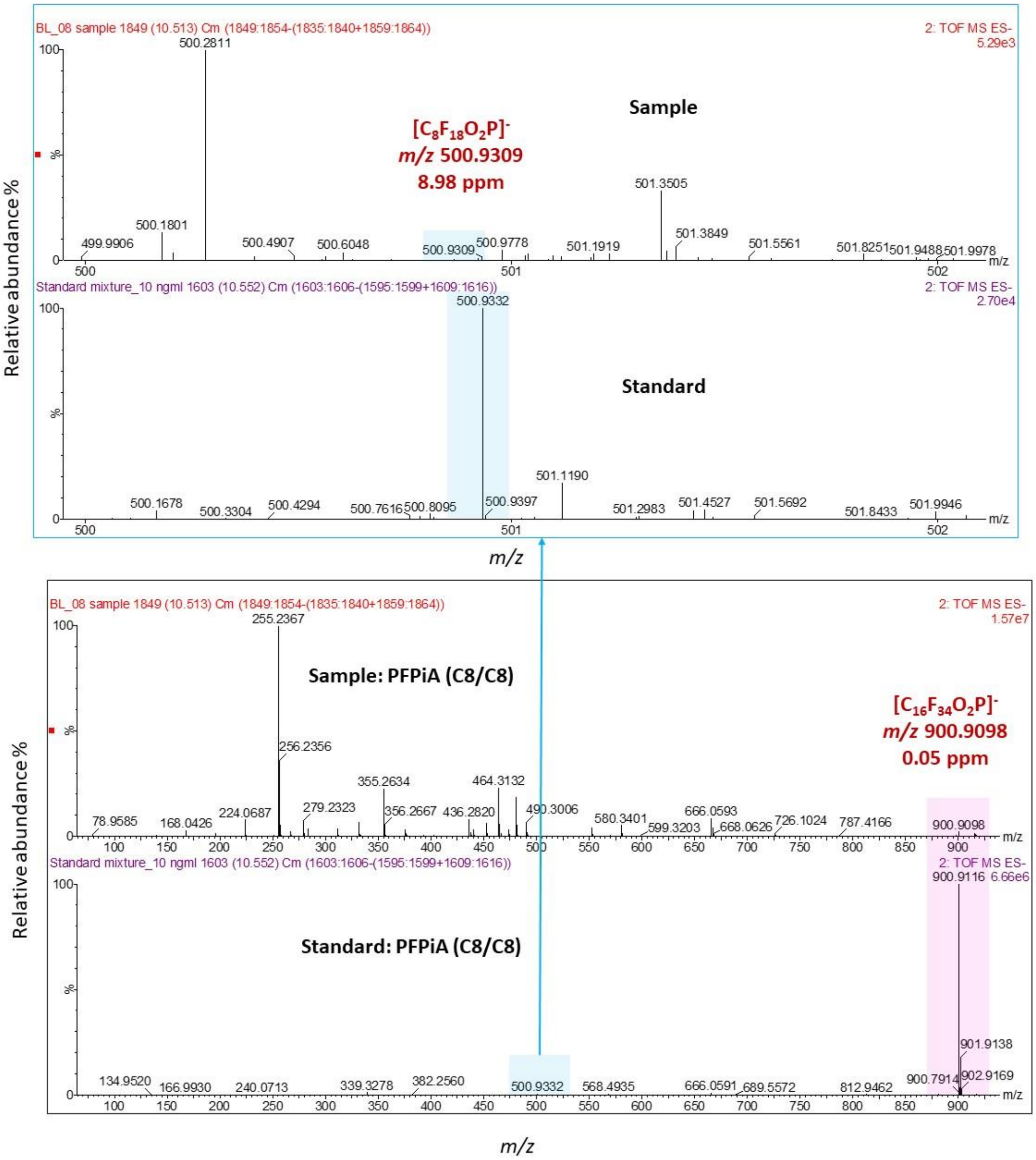

Table S15 – Tentatively identified perfluoroalkyl ether sulfonic acids. No fragment ions were detected in the identified homolog group, thus chemical identifiers are not provided. The table lists the number of corresponding entries for each molecular formula in the PubChem database.

Class	Proposed structure	Acronym	[M-H]$^-$	Molecular formula	*m/z*	Observed *m/z*	Mass accuracy (ppm)	Rt (min.)	CL	#PubChem entries
Perfluoroalkyl ether sulfonic acids	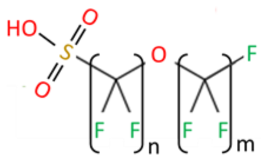	PFESA n+m=7	$[C_7F_{15}O_4S]^-$	$C_7HF_{15}O_4S$	464.92833	464.9267	3.51	7.44	4	7
		PFESA n+m=8	$[C_8F_{17}O_4S]^-$	$C_8HF_{17}O_4S$	514.92513	514.9236	2.97	8.08	4	6
		PFESA n+m=9	$[C_9F_{19}O_4S]^-$	$C_9HF_{19}O_4S$	564.92194	564.9208	2.02	8.72	4	9
		PFESA n+m=10	$[C_{10}F_{21}O_4S]^-$	$C_{10}HF_{21}O_4S$	614.91875	614.9179	1.38	9.32	4	4
		PFESA n+m=11	$[C_{11}F_{23}O_4S]^-$	$C_{11}HF_{23}O_4S$	664.91555	664.9156	0.08	9.89	4	3
		PFESA n+m=12	$[C_{12}F_{25}O_4S]^-$	$C_{12}HF_{25}O_4S$	714.91236	714.9112	1.62	10.21	4	4
		PFESA n+m=13	$[C_{13}F_{27}O_4S]^-$	$C_{13}HF_{27}O_4S$	764.90916	764.9096	0.58	10.34	4	1

Figure S10a – Chromatograms of six homologs belonging to the PFESA group detected in the bream liver sample. Each chromatogram is labeled with the molecular formula of the corresponding homolog. For the homolog highlighted in pink, the fragment ion mass spectrum is displayed, showcasing the isotope pattern for the measured mass (*m/z* 564.9213). The mass spectrum includes the molecular formula of the isotopologues (in red) and the predicted *m/z* values and intensities (in black) of the most intense isotopologues associated with the molecular formula.

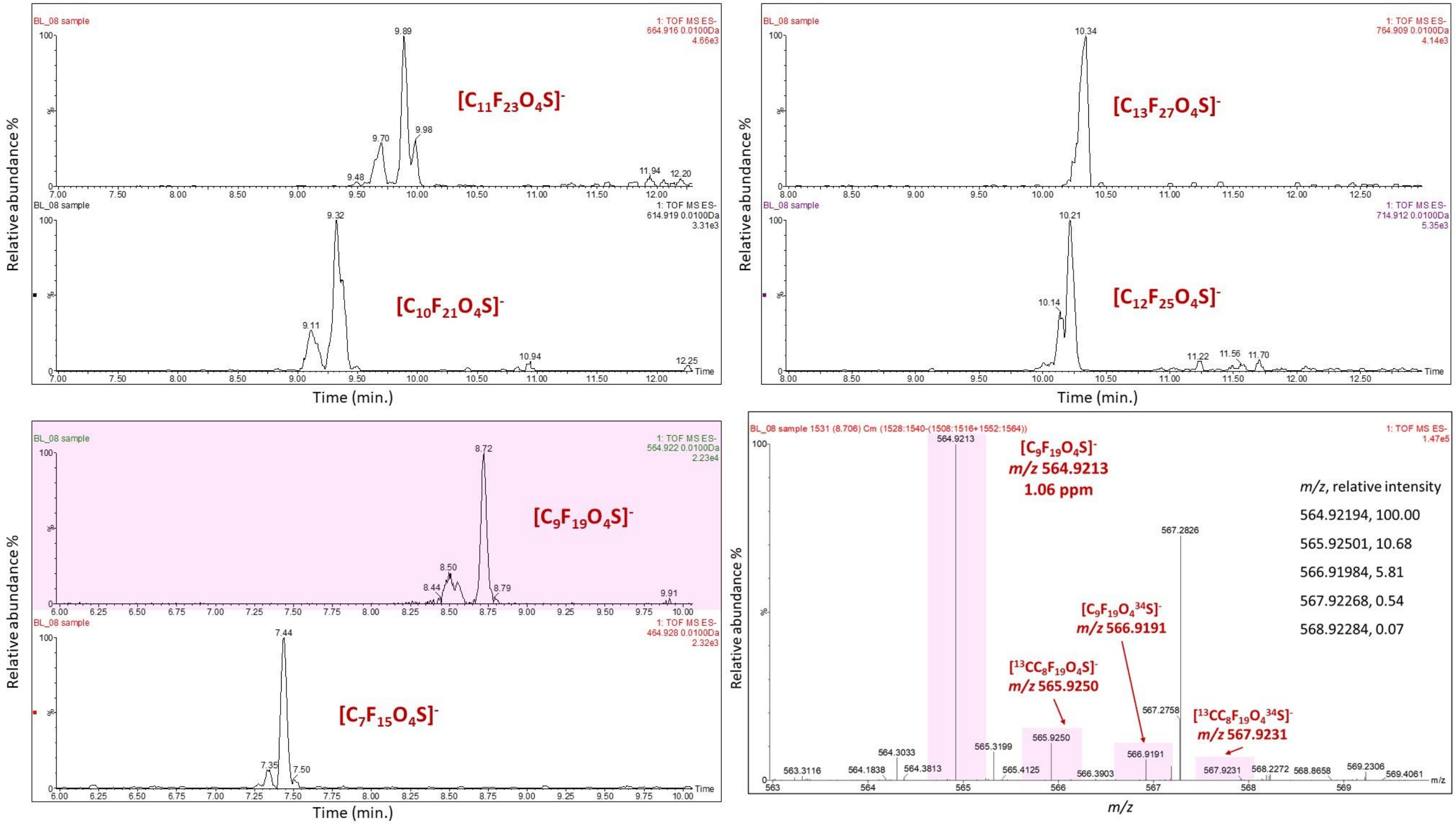

Figure S10b – The figure presents a chromatogram showing the first three homologs from samples collected in 1996 and 2008. Notably, the 2008 sample—which was consistently used to demonstrate our findings—features a peak resembling an electronic signal for the homolog at *m/z* 514.92513. To support our finding, the signal is shown in the 1996 sample (pink color), confirming the presence of the homolog.

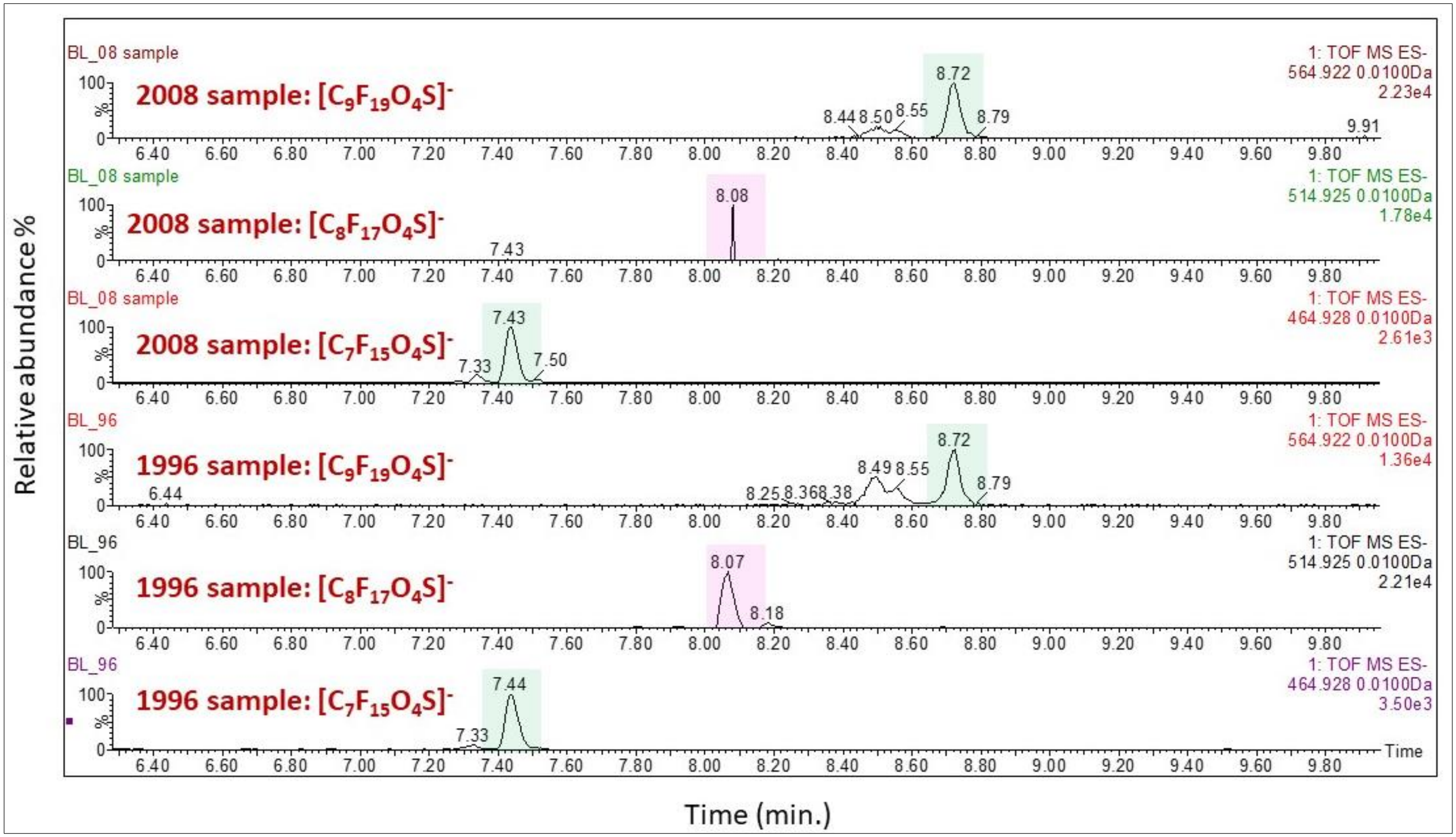

Table S16 – Tentatively identified fluorotelomer alcohol sulfates and their chemical identifiers. The molecular formula $C_9H_5F_{15}O_4S$ lacks a PubChem CID entry, indicating that no matching homolog for FTOH-Sulfates is currently identified in the PubChem database. Additionally, no diagnostic fragments were observed for this group.

Class	Proposed structure	Acronym	$[M-H]^-$	*m/z*	Observed *m/z*	Mass accuracy (ppm)	Rt (min.)	CL
Fluorotelomer sulfates	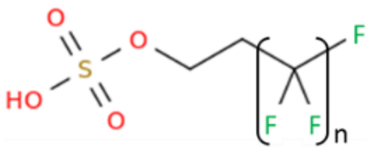	6:2-FTOH-Sulfate	$[C_8H_4F_{13}O_4S]^-$	442.96282	442.9613	3.43	7.04	4
		7:2-FTOH-Sulfate	$[C_9H_4F_{15}O_4S]^-$	492.95963	492.9585	2.29	7.81	4
		8:2-FTOH-Sulfate	$[C_{10}H_4F_{17}O_4S]^-$	542.95643	542.9553	2.08	8.26	4
		10:2-FTOH-Sulfate	$[C_{12}H_4F_{21}O_4S]^-$	642.95005	642.9496	0.70	9.60	4
		12:2-FTOH-Sulfate	$[C_{14}H_4F_{25}O_4S]^-$	742.94366	742.9426	1.43	10.31	4

Class	Molecular formula	IUPAC	SMILES	CAS RN	PubChem CID
Fluorotelomer sulfates	$C_8H_5F_{13}O_4S$	3,3,4,4,5,5,6,6,7,7,8,8,8-tridecafluorooctyl hydrogen sulfate	C(COS(=O)(=O)O)C(C(C(C(C(C(F)(F)F)(F)F)(F)F)(F)F)(F)F)(F)F	82711-15-7	91350838
	$C_9H_5F_{15}O_4S$	3,3,4,4,5,5,6,6,7,7,8,8,9,9,9-pentadecafluorononyloxysulfonic acid	O=S(=O)(O)OCCC(F)(F)C(F)(F)C(F)(F)C(F)(F)C(F)(F)C(F)(F)C(F)(F)F	-	-
	$C_{10}H_5F_{17}O_4S$	3,3,4,4,5,5,6,6,7,7,8,8,9,9,10,10,10-heptadecafluorodecyl hydrogen sulfate	C(COS(=O)(=O)O)C(C(C(C(C(C(C(C(F)(F)F)(F)F)(F)F)(F)F)(F)F)(F)F)(F)F)(F)F	-	15936172
	$C_{12}H_5F_{21}O_4S$	3,3,4,4,5,5,6,6,7,7,8,8,9,9,10,10,11,11,12,12,12-henicosafluorododecyl hydrogen sulfate	C(COS(=O)(=O)O)C(C(C(C(C(C(C(C(C(C(F)(F)F)(F)F)(F)F)(F)F)(F)F)(F)F)(F)F)(F)F)(F)F)(F)F	-	22639323
	$C_{14}H_5F_{25}O_4S$	3,3,4,4,5,5,6,6,7,7,8,8,9,9,10,10,11,11,12,12,13,13,14,14,14-pentacosafluorotetradecyl hydrogen sulfate	C(COS(=O)(=O)O)C(C(C(C(C(C(C(C(C(C(C(C(F)(F)F)(F)F)(F)F)(F)F)(F)F)(F)F)(F)F)(F)F)(F)F)(F)F)(F)F)(F)F	-	138395092

Figure S11 – Chromatograms of five homologs, referred to as FTOH-Sulfates, detected in the bream liver sample, with the acronym of each homolog indicated. The chromatogram highlighted in pink displays the fragment ion mass spectrum for the corresponding homolog, showcasing one detected fragment. No fragments were observed in the fragment ion mass spectrum for the other homologs.

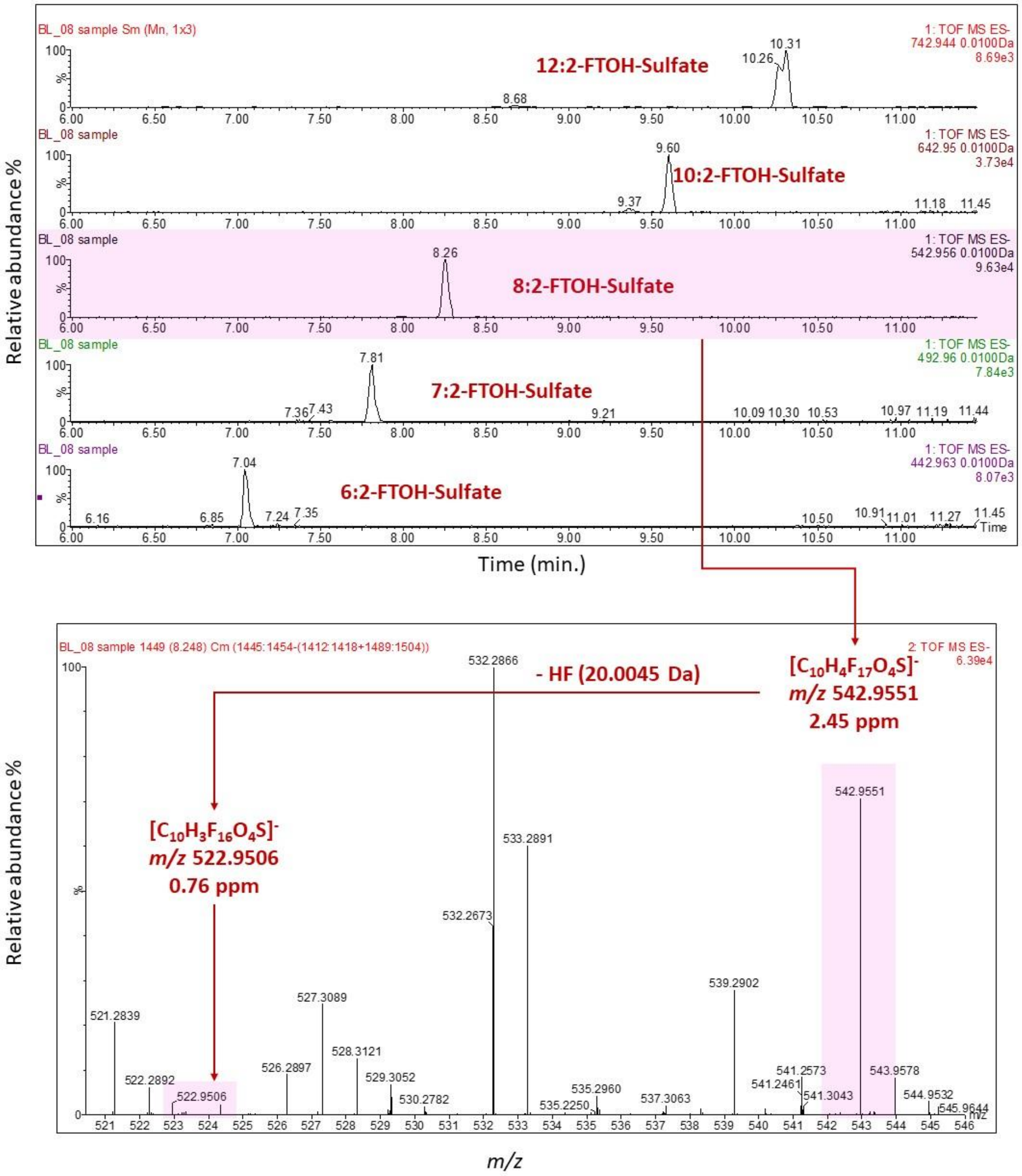

Table S17 – Tentatively identified perfluoroalkyl pentafluorosulfanyl sulfonic acids and their chemical identifiers.

Class	Proposed structure	Acronym	[M-H]$^-$	*m/z*	Observed *m/z*	Mass accuracy (ppm)	Rt (min.)	CL
Perfluoroalkyl pentafluorosulfanyl sulfonic acids	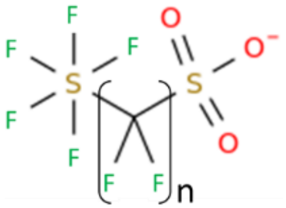	F5S-PFLSA n=8	$[C_8F_{21}O_3S_2]^-$	606.8959	606.8961	0.33	9.19	2b
		F5S-PFLSA n=9	$[C_9F_{23}O_3S_2]^-$	656.89271	656.8929	0.29	9.76	2b
		F5S-PFLSA n=10	$[C_{10}F_{25}O_3S_2]^-$	706.88951	706.8902	0.98	10.19	3d

Molecular formula	IUPAC	SMILES	CAS RN	PubChem CID
$C_8HF_{21}O_3S_2$	1,1,2,2,3,3,4,4,5,5,6,6,7,7,8,8-hexadecafluoro-8-(pentafluoro-λ6-sulfanyl)octane-1-sulfonic acid	C(C(C(C(C(F)(F)S(F)(F)(F)(F)F)(F)F)(F)F)(F)F)(C(C(C(F)(F)S(=O)(=O)O)(F)F)(F)F)(F)F	2089109-34-0	139596679
$C_9HF_{23}O_3S_2$	1,1,2,2,3,3,4,4,5,5,6,6,7,7,8,8,9,9-octadecafluoro-9-(pentafluoro-λ6-sulfanyl)nonane-1-sulfonic acid	C(C(C(C(C(F)(F)S(=O)(=O)O)(F)F)(F)F)(F)F)(C(C(C(C(F)(F)S(F)(F)(F)(F)F)(F)F)(F)F)(F)F)(F)F	2089109-35-1	139595118
$C_{10}HF_{25}O_3S_2$	1,1,2,2,3,3,4,4,5,5,6,6,7,7,8,8,9,9,10,10-icosafluoro-10-(pentafluoro-λ6-sulfanyl)decane-1-sulfonic acid	C(C(C(C(C(C(F)(F)S(F)(F)(F)(F)F)(F)F)(F)F)(F)F)(F)F)(C(C(C(C(F)(F)S(=O)(=O)O)(F)F)(F)F)(F)F)(F)F	-	163323945

Figure S12a – Chromatogram of three homologs corresponding to the F5S-PFLSA group. The upper section (black outline) shows the chromatograms for each homolog, where *n* indicates the number of fully fluorinated carbons. The bottom chromatogram (blue outline) displays the fragments detected in the F5S-PFLSA homolog with *n = 9*, highlighting the fragmentation pattern specific to this compound.

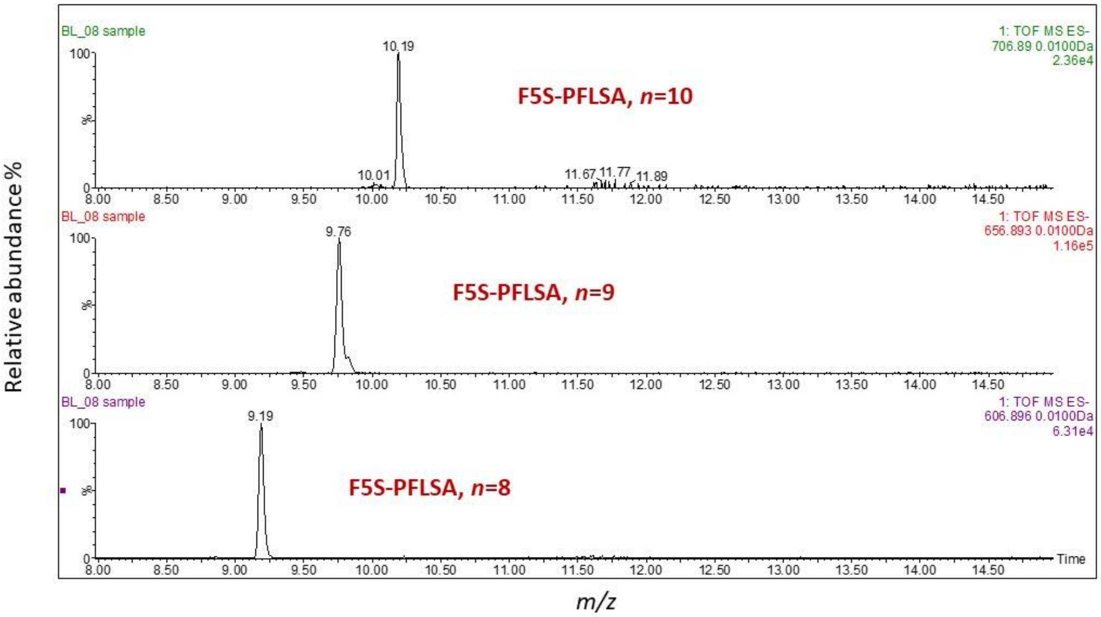

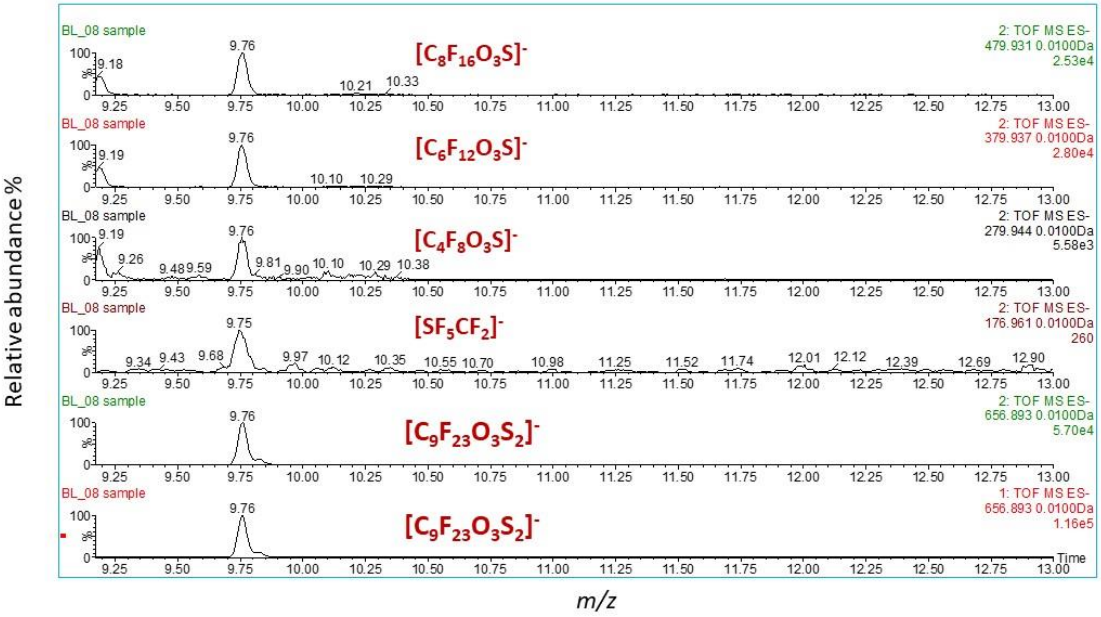

Figure S12b – Fragment ion mass spectrum corresponding to F5S-PFLSA, *n* = 8. The spectrum displays the molecular ion, while the upper three cropped mass spectra focus on the fragments that become visible upon zooming into the mass range where these fragments are located.

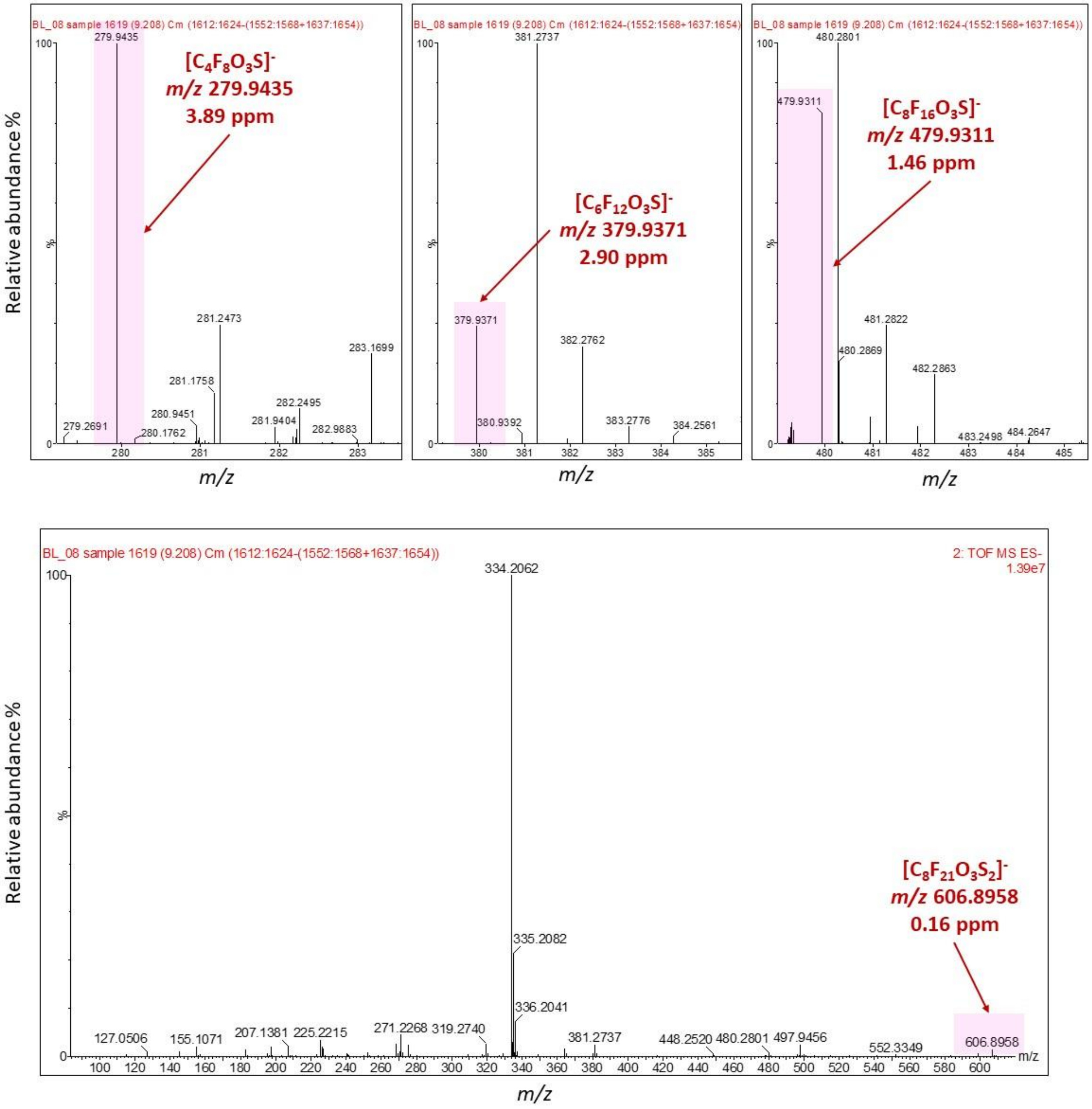

Figure S12c – Fragment ion mass spectrum corresponding to F5S-PFLSA, *n* = 9. The spectrum displays the molecular ion along with three observed fragments for F5S-PFLSA, *n* = 9. Notably, the fragment at *m/z* 176.9606 serves as a diagnostic fragment for this group and was detected exclusively in this homolog.

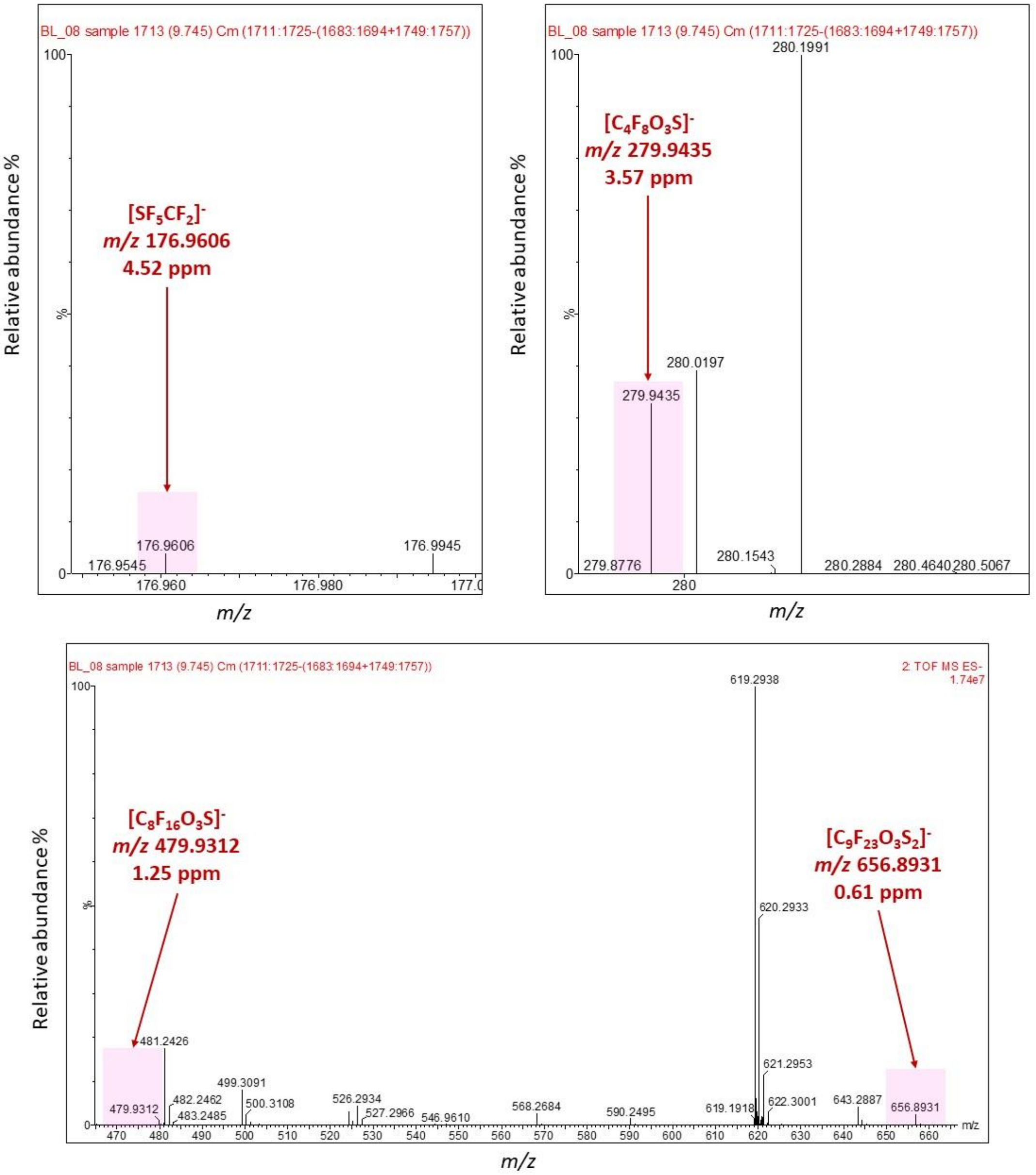

Figure S12d – Fragment ion mass spectrum corresponding to F5S-PFLSA, n = 9. In the full scan without magnification, neither the molecular ion nor fragments are visible. The upper spectrum shows the molecular ion after zooming in, along with the corresponding isotopologues (in red). The mass spectrum also includes simulated data for the five most intense isotopologues and their relative intensities based on the given molecular formula.

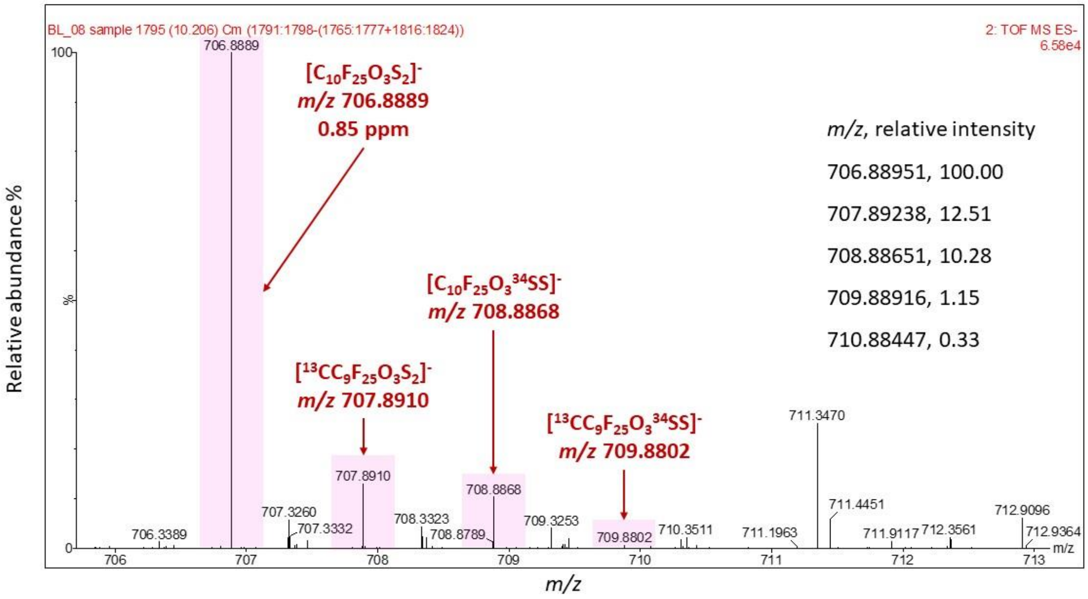

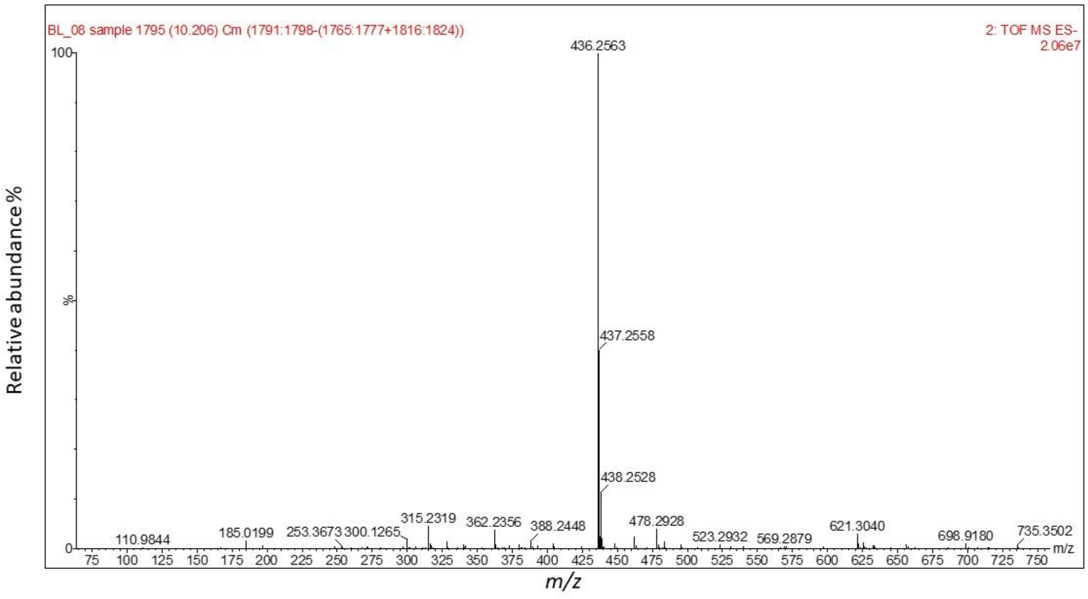

Table S18 – Tentatively identified polyfluoroalkyl sulfinates (n:1 PFESu) and their chemical identifiers. The asterisk in the column labeled "PubChem CID" indicates the molecular formula as listed in the PubChem database. However, based on our findings, the structure presented in PubChem is unlikely to be correct, as it lacks an ionizable site necessary for detection in negative electrospray ionization (ESI) mode.

Class	Proposed structure	[M-H]⁻	*m/z*	Observed *m/z*	Mass accuracy (ppm)	Rt (min.)	CL
Polyfluoroalkyl sulfinates	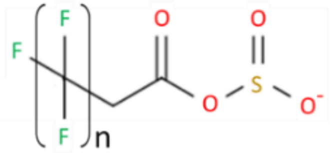	$[C_8H_2F_{13}O_4S]^-$	440.94717	440.94556	3.65	7.09	4
		$[C_{10}H_2F_{17}O_4S]^-$	540.94078	540.9399	1.63	8.33	3d
		$[C_{12}H_2F_{21}O_4S]^-$	640.93440	640.9353	1.41	9.68	3d
		$[C_{14}H_2F_{25}O_4S]^-$	740.92801	740.9288	1.07	10.32	4

Molecular formula	IUPAC	SMILES	CAS RN	PubChem CID
$C_8H_3F_{13}O_4S$	sulfino 3,3,4,4,5,5,6,6,7,7,8,8,8-tridecafluorooctanoate	O=C(CC(F)(F)C(F)(F)C(F)(F)C(F)(F)C(F)(F)C(F)(F)F)OS(=O)O	-	22759348*
$C_{10}H_3F_{17}O_4S$	sulfino 3,3,4,4,5,5,6,6,7,7,8,8,9,9,10,10,10-heptadecafluorodecanoate	O=C(CC(F)(F)C(F)(F)C(F)(F)C(F)(F)C(F)(F)C(F)(F)C(F)(F)C(F)(F)F)OS(=O)O	-	-
$C_{12}H_3F_{21}O_4S$	sulfino 3,3,4,4,5,5,6,6,7,7,8,8,9,9,10,10,11,11,12,12,12-henicosafluorododecanoate	O=C(CC(F)(F)C(F)(F)C(F)(F)C(F)(F)C(F)(F)C(F)(F)C(F)(F)C(F)(F)C(F)(F)C(F)(F)F)OS(=O)O	-	-
$C_{14}H_3F_{23}O_4S$	sulfino 3,3,4,4,5,5,6,6,7,7,8,8,9,9,10,10,11,11,12,12,13,13,14,14,14-pentacosafluorotetradecanoate	O=C(CC(F)(F)C(F)(F)C(F)(F)C(F)(F)C(F)(F)C(F)(F)C(F)(F)C(F)(F)C(F)(F)C(F)(F)C(F)(F)C(F)(F)F)OS(=O)O	-	-

Figure S13a – Chromatograms of four homologs categorized as n:1 PFESu. The chromatogram displays the molecular formula (in red) alongside the corresponding eluting peaks, indicating the detection and retention times of these unknown compounds.

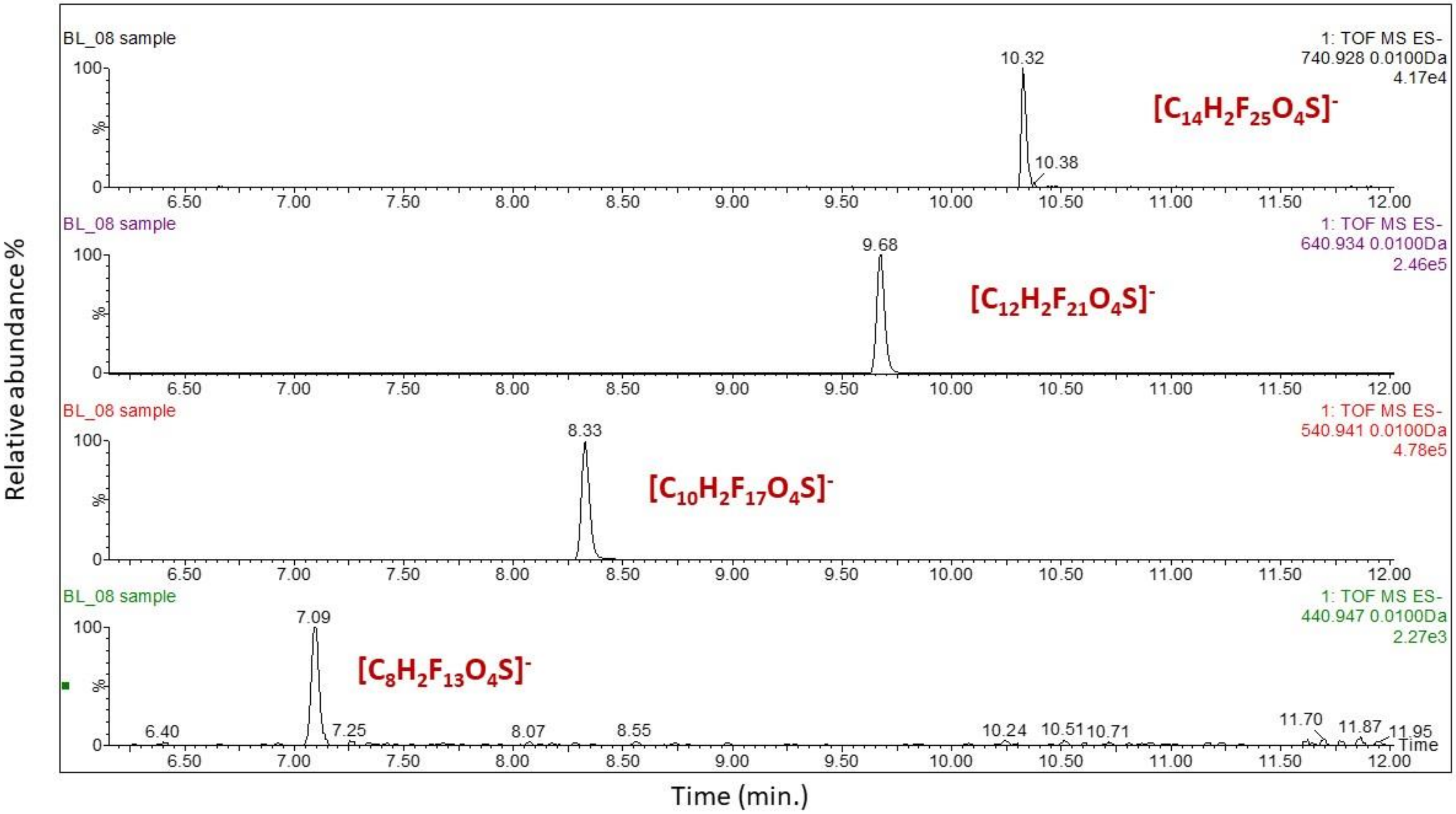

Figure S13b – Fragment ion mass spectrum showing the molecular ion of the homolog (*m/z* 440.9442) detected in the bream liver sample. No fragments were observed for this homolog, and therefore only the molecular ion is highlighted in yellow.

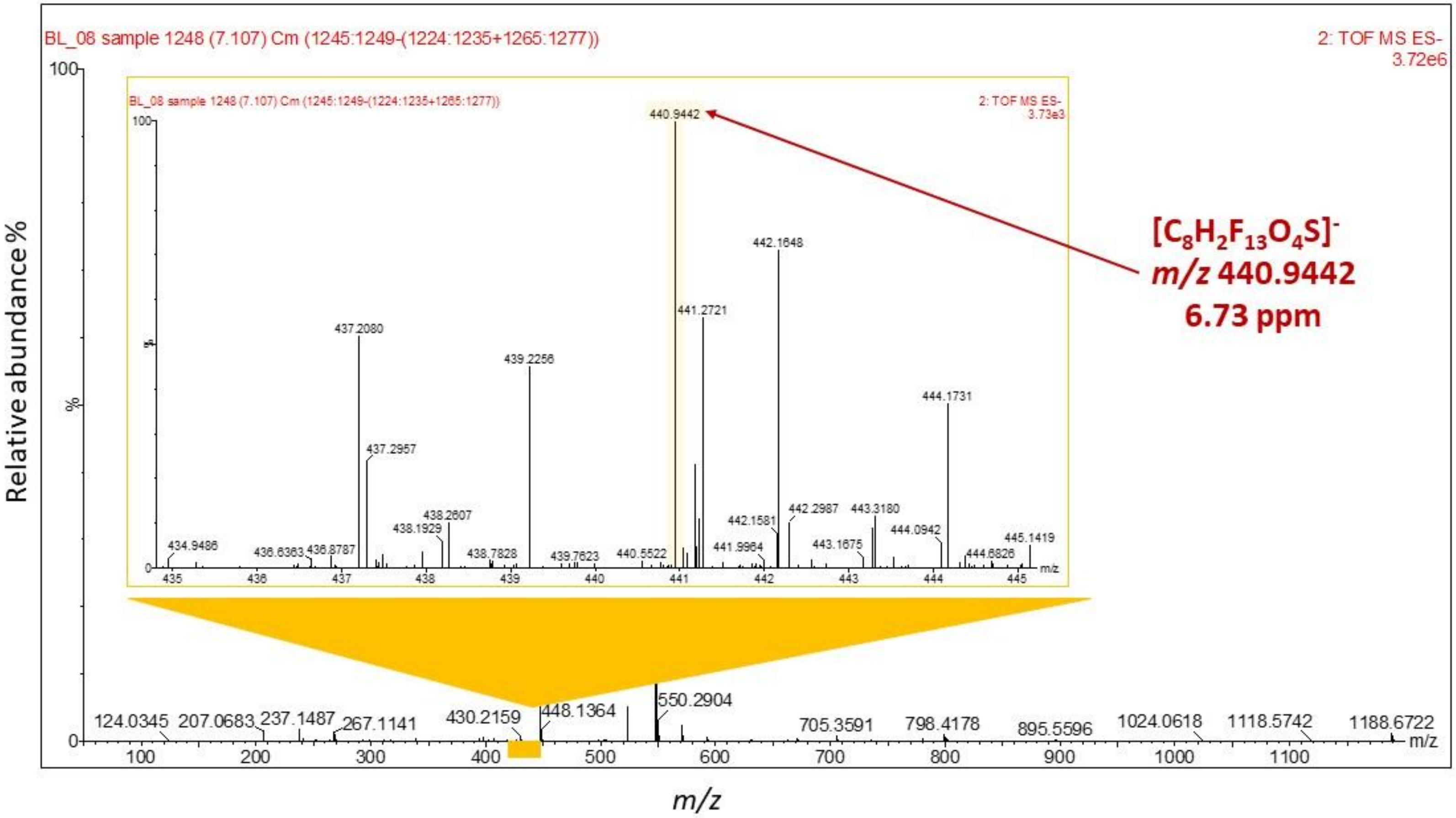

Figure S13c – Fragment ion mass spectrum of 8:1 PFESu with a mass of *m/z* 540.9400. The spectrum shows the molecular ion (yellow color) along with three observed fragments (pink color), highlighting the fragmentation pattern for this homolog detected in the bream liver sample.

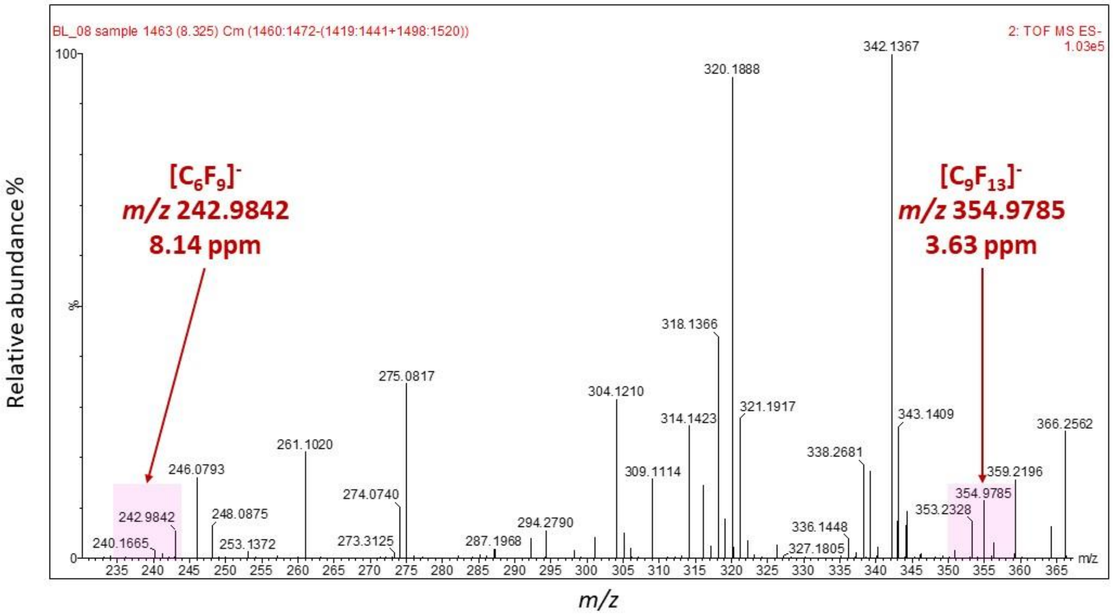

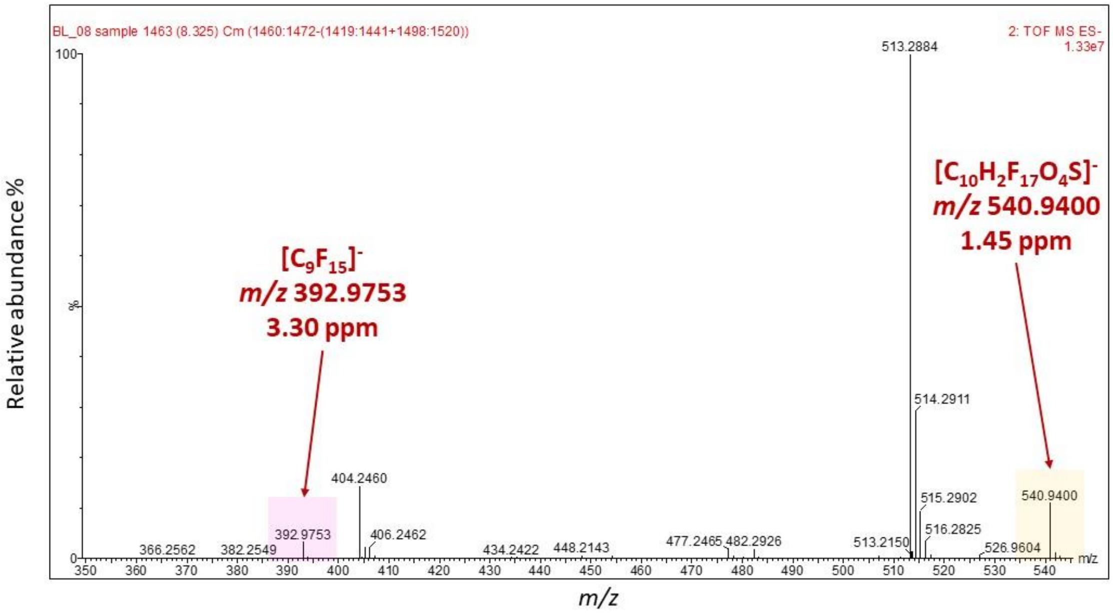

Figure S13d – Fragment ion mass spectrum of 10:1 PFESu with a measured mass of *m/z* 640.9350, highlighted in yellow as the molecular ion. Three additional fragments were detected and are shown in pink, illustrating the fragmentation pattern for this homolog.

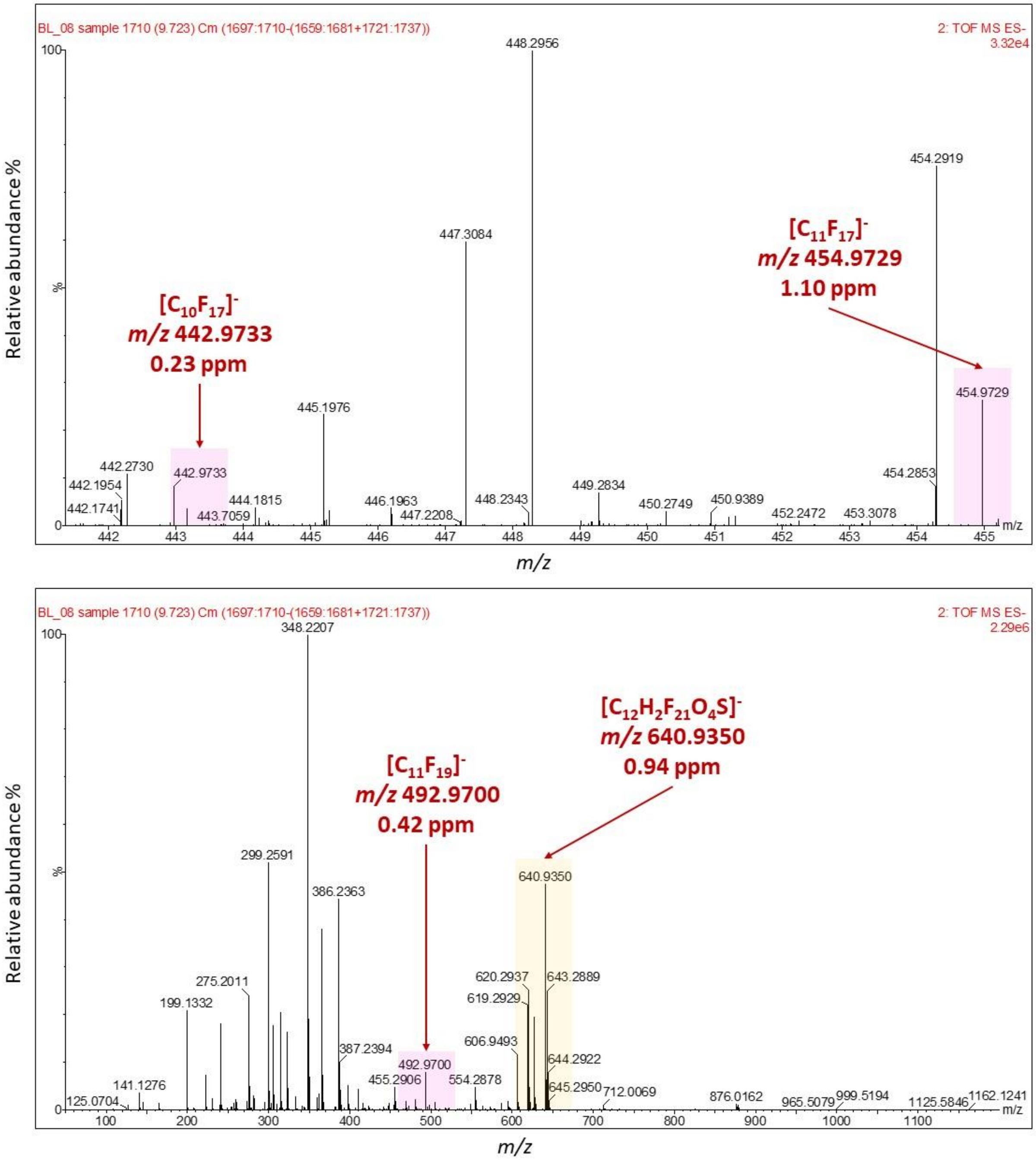

Figure S13e – Zoomed fragment ion mass spectrum of the 12:1 PFESu, detected at *m/z* 740.9281. Only one fragment was observed, which is highlighted in pink.

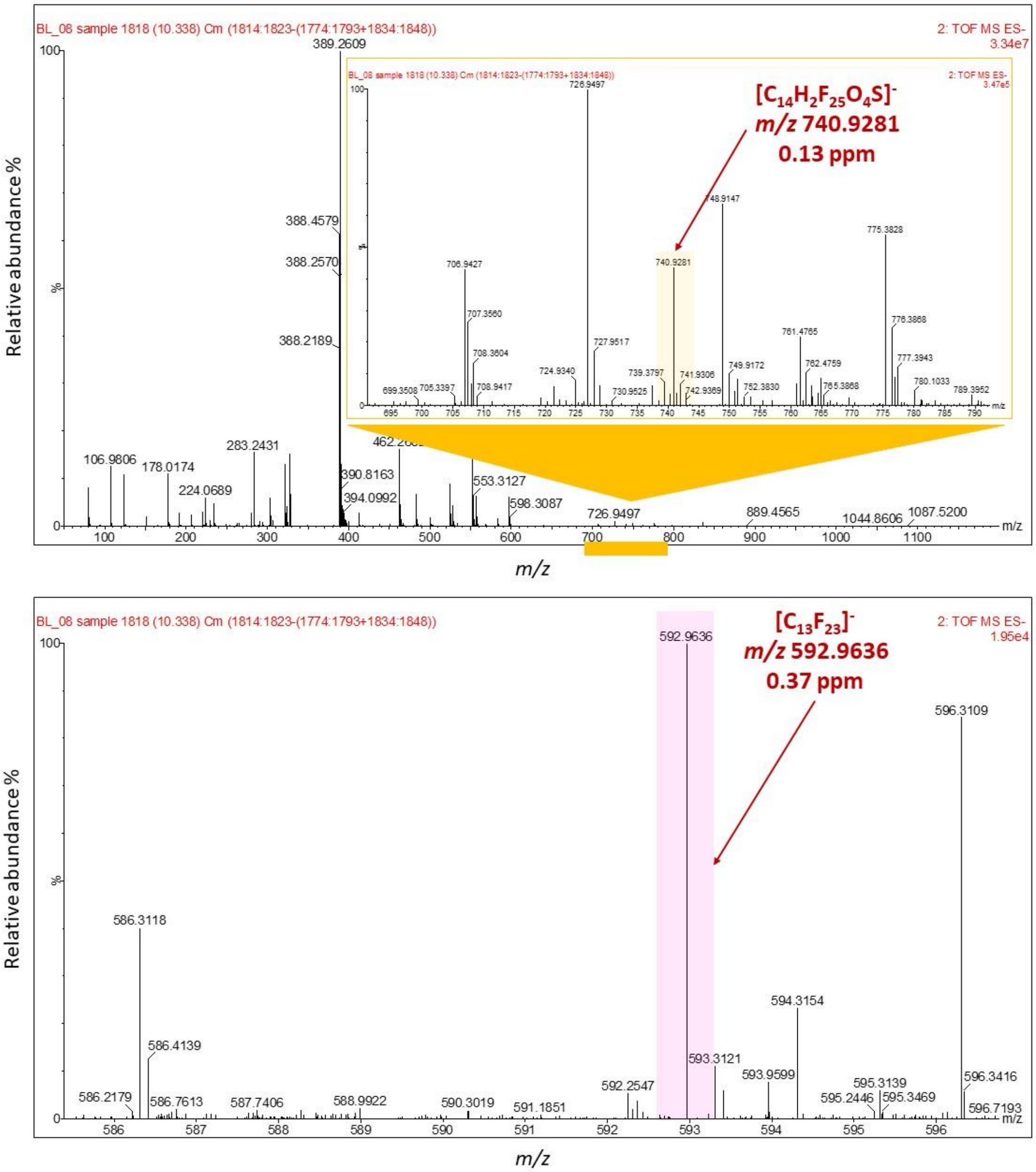

Table S19 – Tentatively identified polyfluoroalkyl sulfonyl sulfonamides and their chemical identifiers.

Class	Proposed structure	Acronym	$[M-H]^-$	*m/z*	Observed *m/z*	Mass accuracy (ppm)	Rt (min.)	CL
n:2 FTSAm	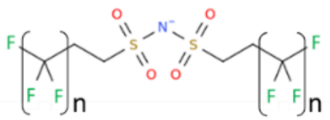	$[C_{16}H_9F_{26}NO_4S_2]$	$[C_{16}H_8F_{26}NO_4S_2]^-$	835.94937	835.9497	0.39	10.30	3b
		$[C_{18}H_9F_{30}NO_4S_2]$	$[C_{18}H_8F_{30}NO_4S_2]^-$	935.94299	935.9423	0.74	10.45	3b
		$[C_{20}H_9F_{34}NO_4S_2]$	$[C_{20}H_8F_{34}NO_4S_2]^-$	1035.93660	1035.9330	3.48	10.56	4

Class	Molecular formula	IUPAC	SMILES	CAS RN	PubChem CID
n:2 FTSAm	$C_{16}H_9F_{26}NO_4S_2$	3,3,4,4,5,5,6,6,7,7,8,8,8-tridecafluoro-N-(3,3,4,4,5,5,6,6,7,7,8,8,8-tridecafluorooctanesulfonyl)octane-1-sulfonamide	O=S(=O)(CCC(F)(F)C(F)(F)C(F)(F)C(F)(F)C(F)(F)C(F)(F)F)NS(=O)(=O)CCC(F)(F)C(F)(F)C(F)(F)C(F)(F)C(F)(F)C(F)(F)F	-	-
	$C_{18}H_9F_{30}NO_4S_2$	3,3,4,4,5,5,6,6,7,7,8,8,9,9,9-pentadecafluoro-N-(3,3,4,4,5,5,6,6,7,7,8,8,9,9,9-pentadecafluorononanesulfonyl)nonane-1-sulfonamide	O=S(=O)(CCC(F)(F)C(F)(F)C(F)(F)C(F)(F)C(F)(F)C(F)(F)C(F)(F)F)NS(=O)(=O)CCC(F)(F)C(F)(F)C(F)(F)C(F)(F)C(F)(F)C(F)(F)C(F)(F)F	-	-
	$C_{20}H_9F_{34}NO_4S_2$	3,3,4,4,5,5,6,6,7,7,8,8,9,9,10,10,10-heptadecafluoro-N-(3,3,4,4,5,5,6,6,7,7,8,8,9,9,10,10,10-heptadecafluorodecanesulfonyl)decane-1-sulfonamide	O=S(=O)(CCC(F)(F)C(F)(F)C(F)(F)C(F)(F)C(F)(F)C(F)(F)C(F)(F)C(F)(F)F)NS(=O)(=O)CCC(F)(F)C(F)(F)C(F)(F)C(F)(F)C(F)(F)C(F)(F)C(F)(F)C(F)(F)F	-	-

Figure S14a – The figure shows a chromatogram of three homologs classified as n:2 FTSAm. The homolog at *m/z* 835.9493 was detected in the analytical standard of DPOSA, and the corresponding chromatogram is displayed. The fragment ion mass spectrum highlights the molecular ion (yellow) and its isotopologues (red). On the right, the simulated isotopologue pattern for the proposed molecular formula is shown, featuring the five most intense isotopologues with their respective masses and relative intensities.

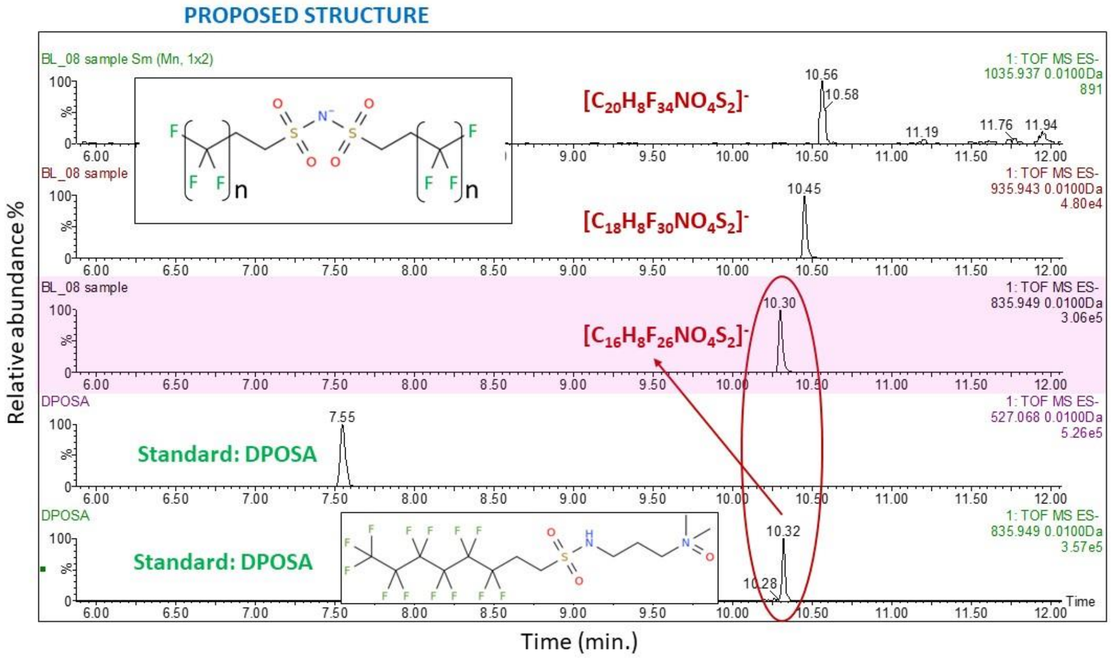

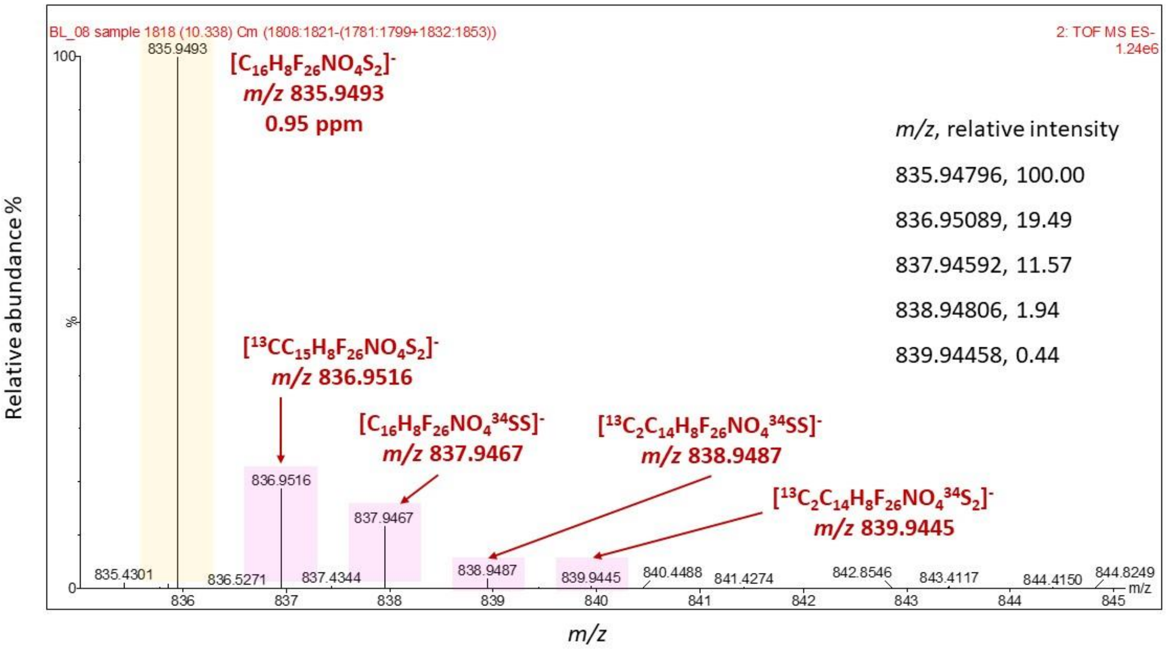

Figure S14b – The figure shows the chromatogram of molecular ion (*m/z* 835.9493) alongside the corresponding fragment ion mass spectrum. The highlighted section of the chromatogram corresponds to the highlighted *m/z* value in the mass spectrum.

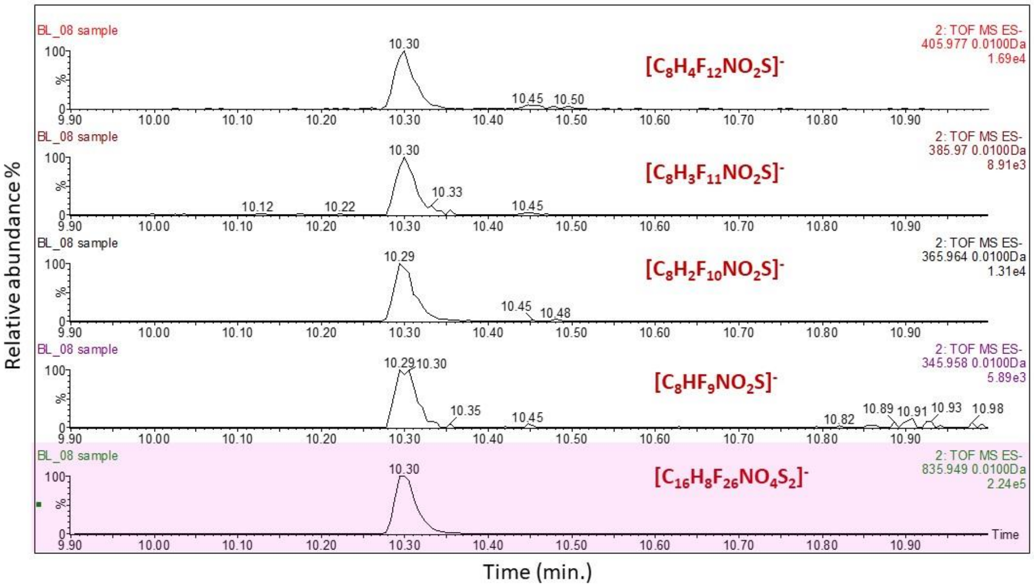

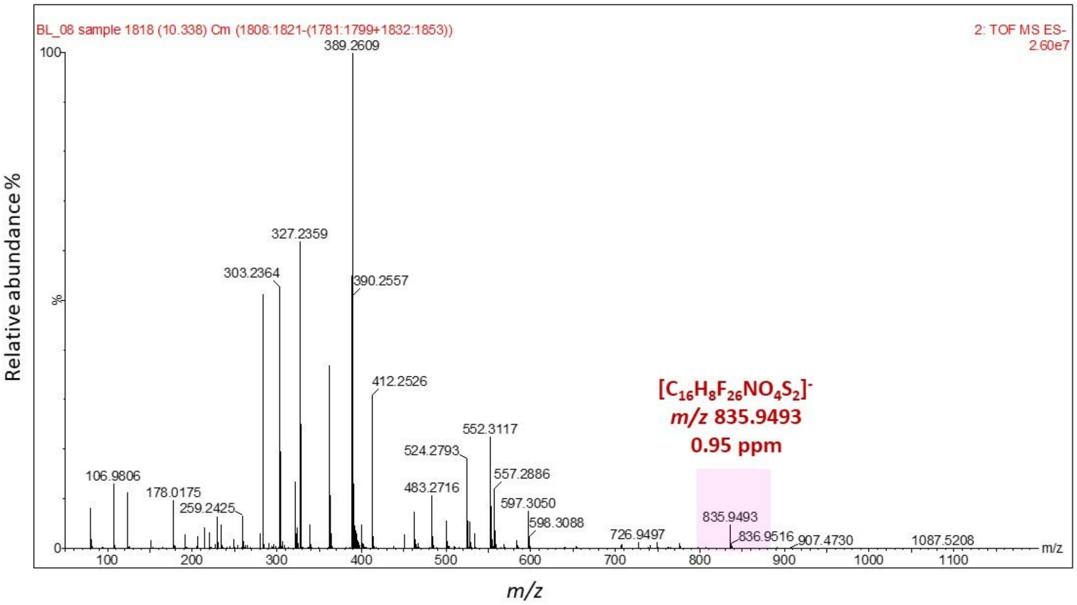

Figure S14c – The figure shows the chromatogram alongside the corresponding fragment ion mass spectrum fragment for 6:2/6:2 FTSAm (*m/z* 835.9493). The highlighted section of the chromatogram corresponds to the highlighted *m/z* value in the fragment ion spectrum, indicating the detected fragment.

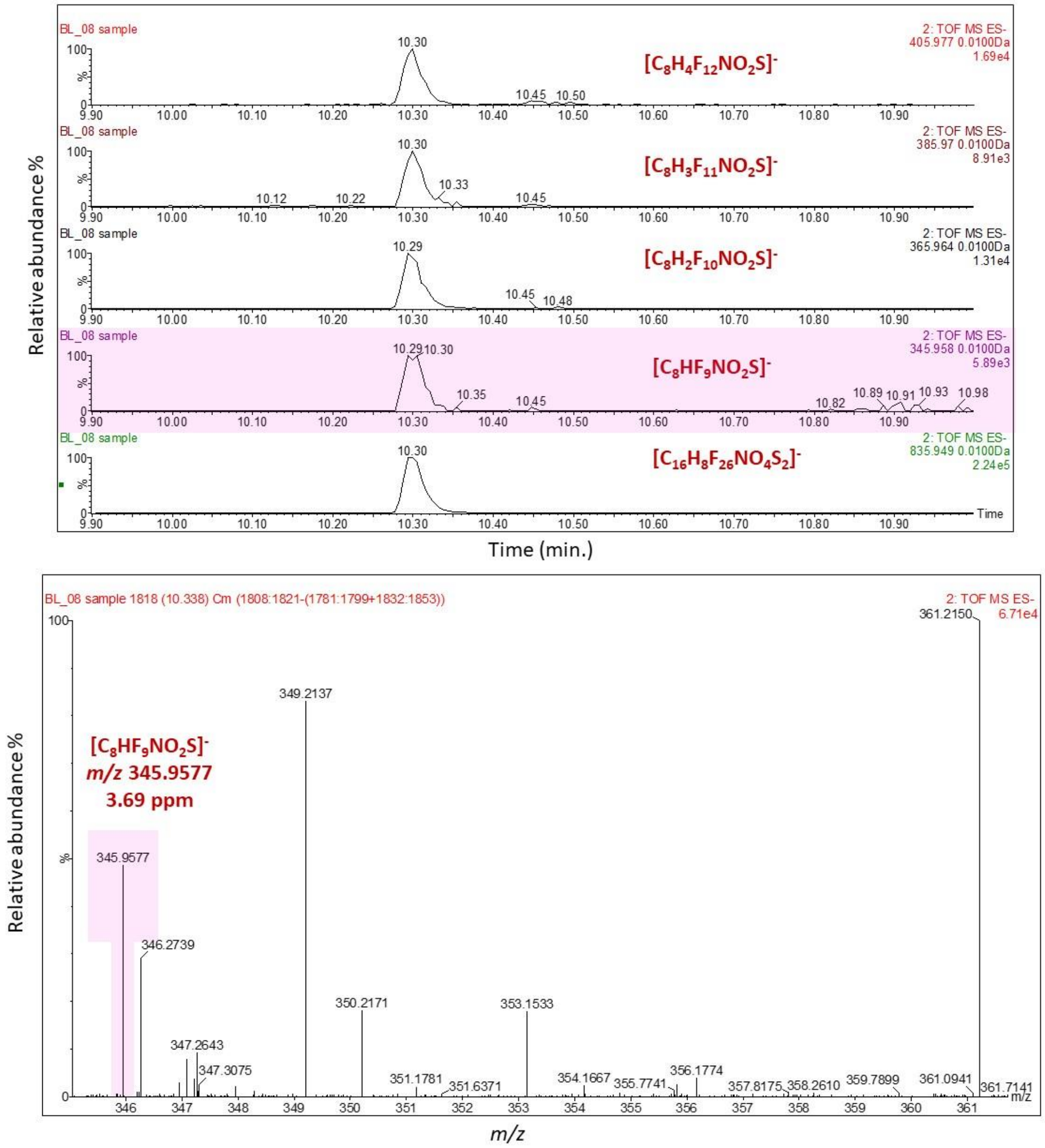

Figure S14d – The figure shows the chromatogram alongside the corresponding fragment ion mass spectrum fragments of 6:2/6:2 FTSAm (*m/z* 835.9493). The highlighted section of the chromatogram corresponds to the highlighted *m/z* values in the fragment ion spectrum, indicating the detected fragments.

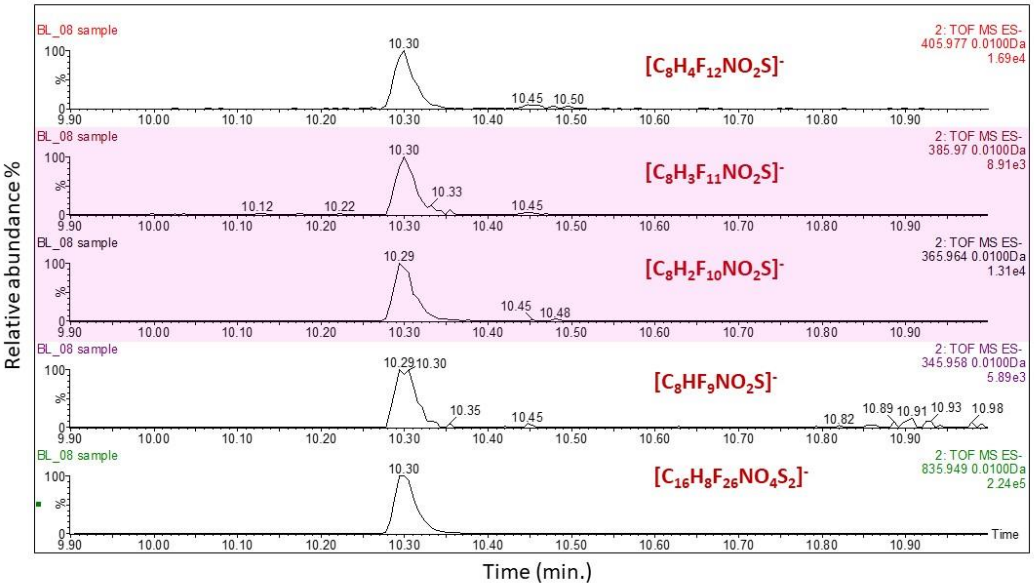

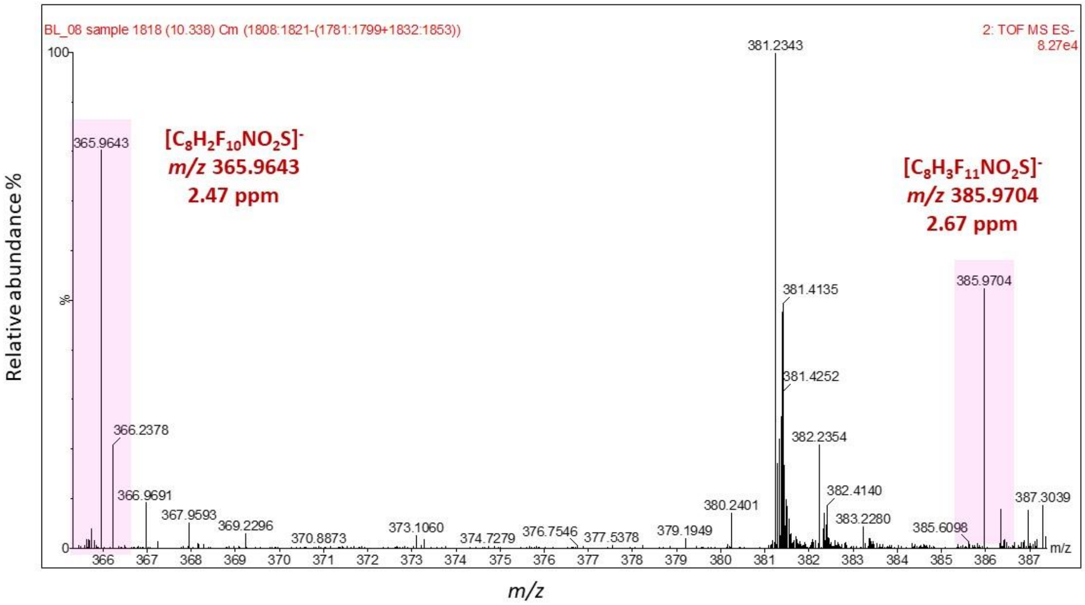

Figure S14e – The figure shows the chromatogram alongside the corresponding fragment ion mass spectrum fragment for 6:2/6:2 FTSAm (*m/z* 835.9493). The highlighted section of the chromatogram corresponds to the highlighted *m/z* value in the fragment ion spectrum, indicating the detected fragment.

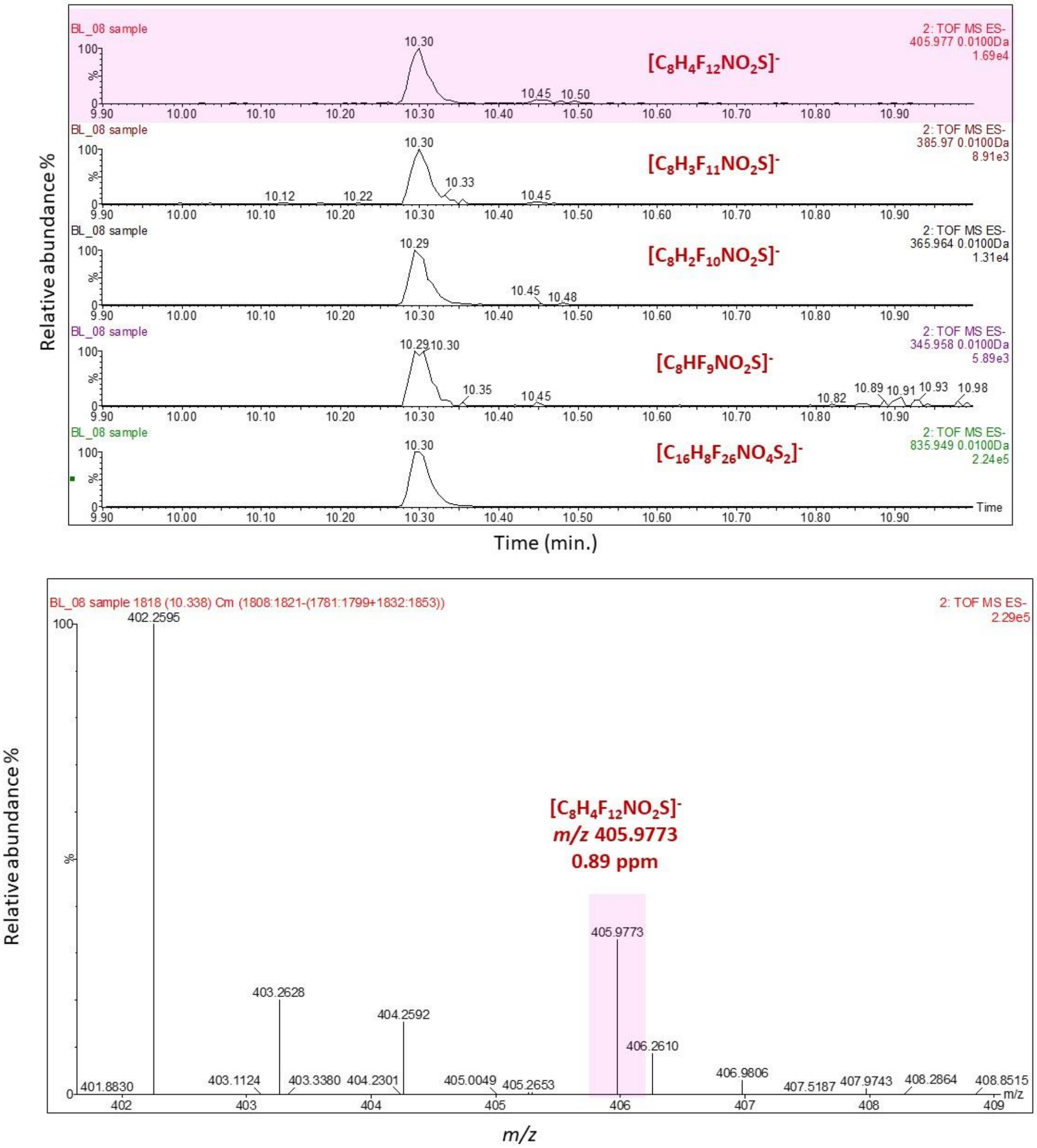

Figure S14f – The chromatogram displays the fragments detected 6:2/8:2 FTSAm (*m/z* 935.9427), highlighting the fragmentation pattern specific to this compound.

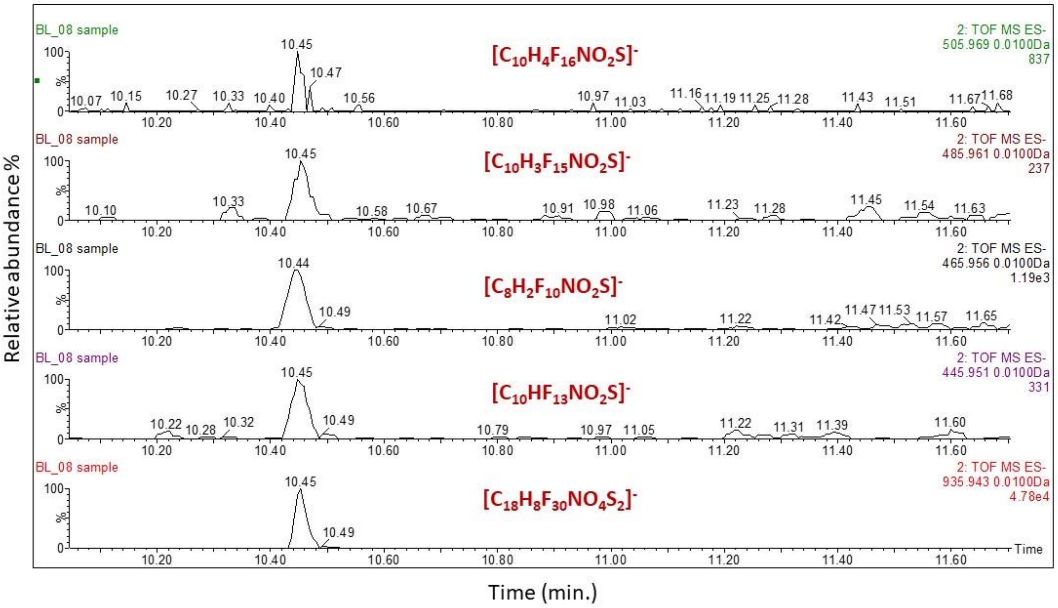

Figure S14g – Fragment ion mass spectrum of 6:2/8:2 FTSAm (*m/z* 935.9427). The molecular ion and two fragments are highlighted in the zoomed-in mass spectrum A, with two additional fragments shown in zoomed-in spectrum B.

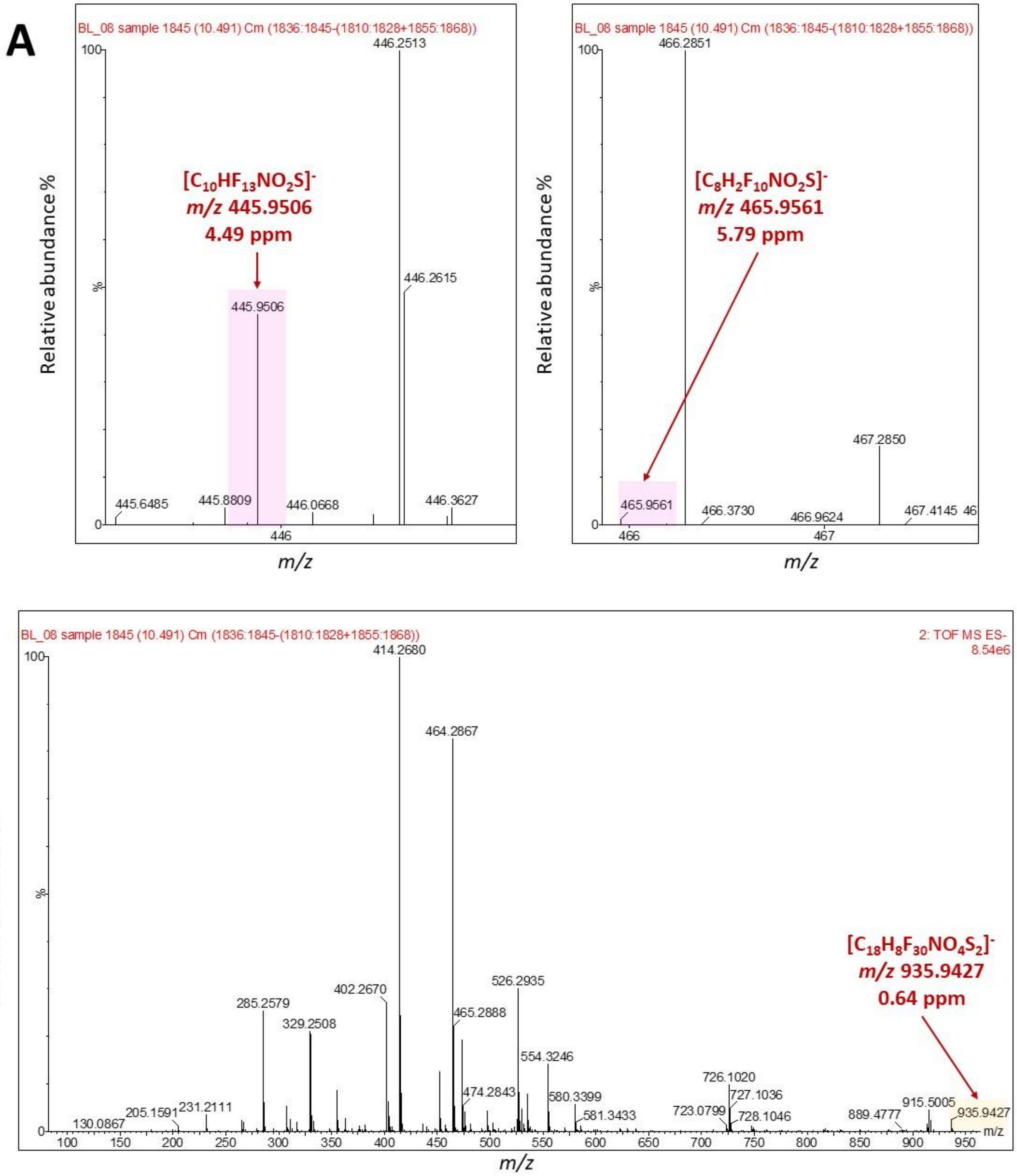

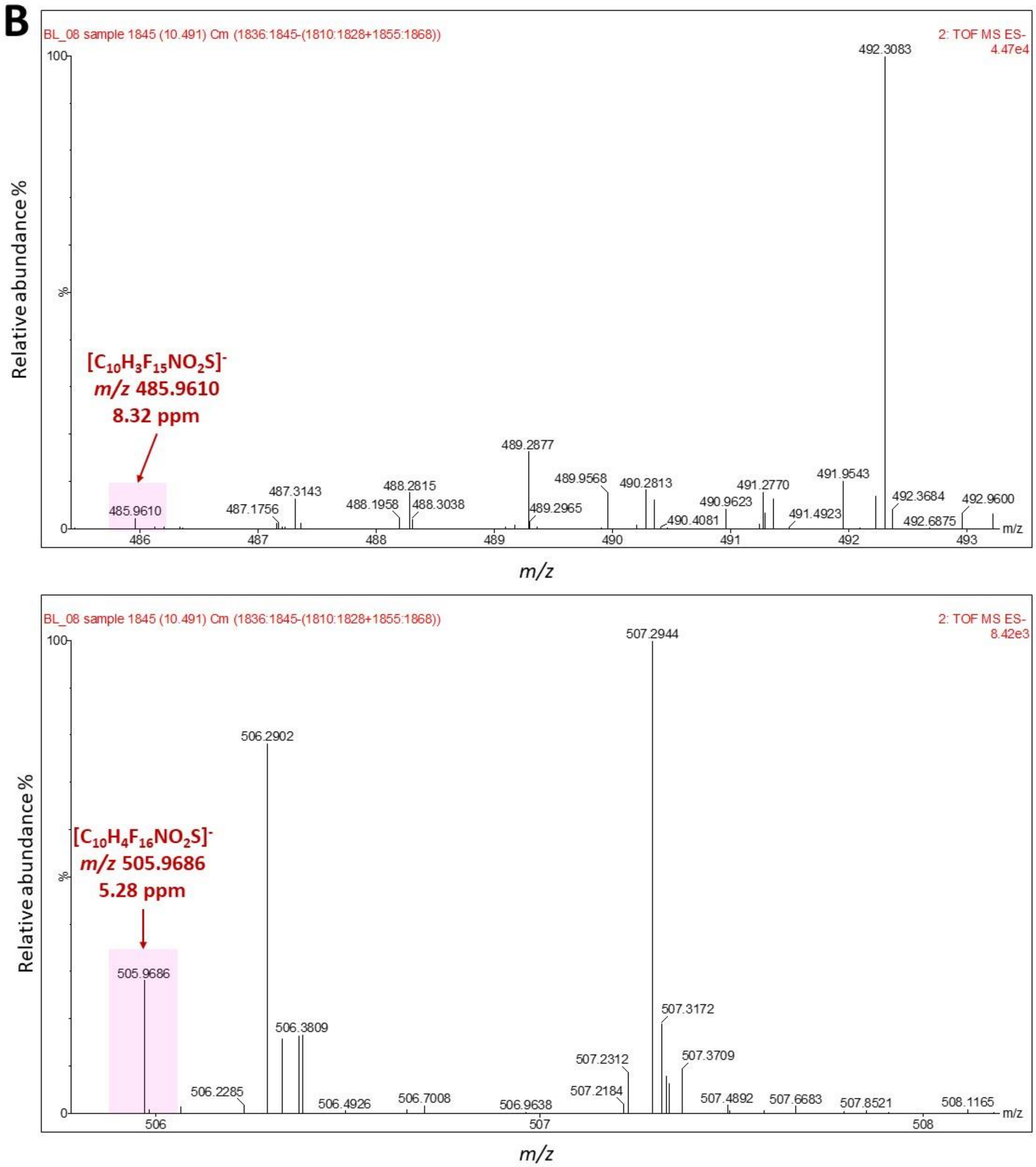
B
BL_08 sample 1845 (10.491) Cm (1836:1845-(1810:1828+1855:1868))
2: TOF MS ES-
4.47e4
Relative abundance %
[$C_{10}H_3F_{15}NO_2S$]$^-$
m/z 485.9610
8.32 ppm
485.9610
487.1756
487.3143
488.1958
488.2815
488.3038
489.2877
489.2965
489.9568
490.2813
490.4081
490.9623
491.2770
491.4923
491.9543
492.3083
492.3684
492.6875
492.9600
m/z
BL_08 sample 1845 (10.491) Cm (1836:1845-(1810:1828+1855:1868))
2: TOF MS ES-
8.42e3
Relative abundance %
[$C_{10}H_4F_{16}NO_2S$]$^-$
m/z 505.9686
5.28 ppm
505.9686
506.2285
506.2902
506.3809
506.4926
506.7008
506.9638
507.2184
507.2312
507.2944
507.3172
507.3709
507.4892
507.6683
507.8521
508.1165
m/z

Table S20 – Tentatively identified perfluoroalkyl sulfonyl amino acids. No diagnostic fragments were observed for this group. The table provides the number of entries found in the PubChem database for the corresponding molecular formula.

Class	Molecular formula	$[M-H]^-$	*m/z*	Observed *m/z*	Mass accuracy (ppm)	Rt (min.)	CL	PubChem entries	PubChem CID
Unknown	$C_{13}H_{10}F_{17}NO_4S$	$[C_{13}H_9F_{17}NO_4S]^-$	597.99863	597.9969	2.89	8.25	4	15	86229431<br>162786<br>6455028<br>18402033<br>20272204<br>53737205<br>92036866<br>153706121<br>157067787<br>101646941<br>101646944<br>140420497<br>162541618<br>163324923<br>166652815
	$C_{14}H_{10}F_{19}NO_4S$	$[C_{14}H_9F_{19}NO_4S]^-$	647.99544	647.9947	1.14	8.94	4	-	
	$C_{15}H_{10}F_{21}NO_4S$	$[C_{15}H_9F_{21}NO_4S]^-$	697.99224	697.9913	1.35	9.61	4	1	163324777
	$C_{16}H_{10}F_{23}NO_4S$	$[C_{16}H_9F_{23}NO_4S]^-$	747.98905	747.9884	0.87	10.13	4	-	
	$C_{17}H_{10}F_{25}NO_4S$	$[C_{17}H_9F_{25}NO_4S]^-$	797.98586	797.9869	1.30	10.32	4	-	
	$C_{18}H_{10}F_{27}NO_4S$	$[C_{18}H_9F_{27}NO_4S]^-$	847.98266	847.9829	0.28	10.41	4	-	

Figure S15 – Chromatograms of six homologs classified as "Unknown" in this study. No fragments were observed for this group. The figure includes the mass spectrum of the highlighted homolog, showing the molecular ion (yellow) and its isotopologues (red). The predicted *m/z* values and intensities for the given molecular formula are also displayed in the precursor ion mass spectrum.

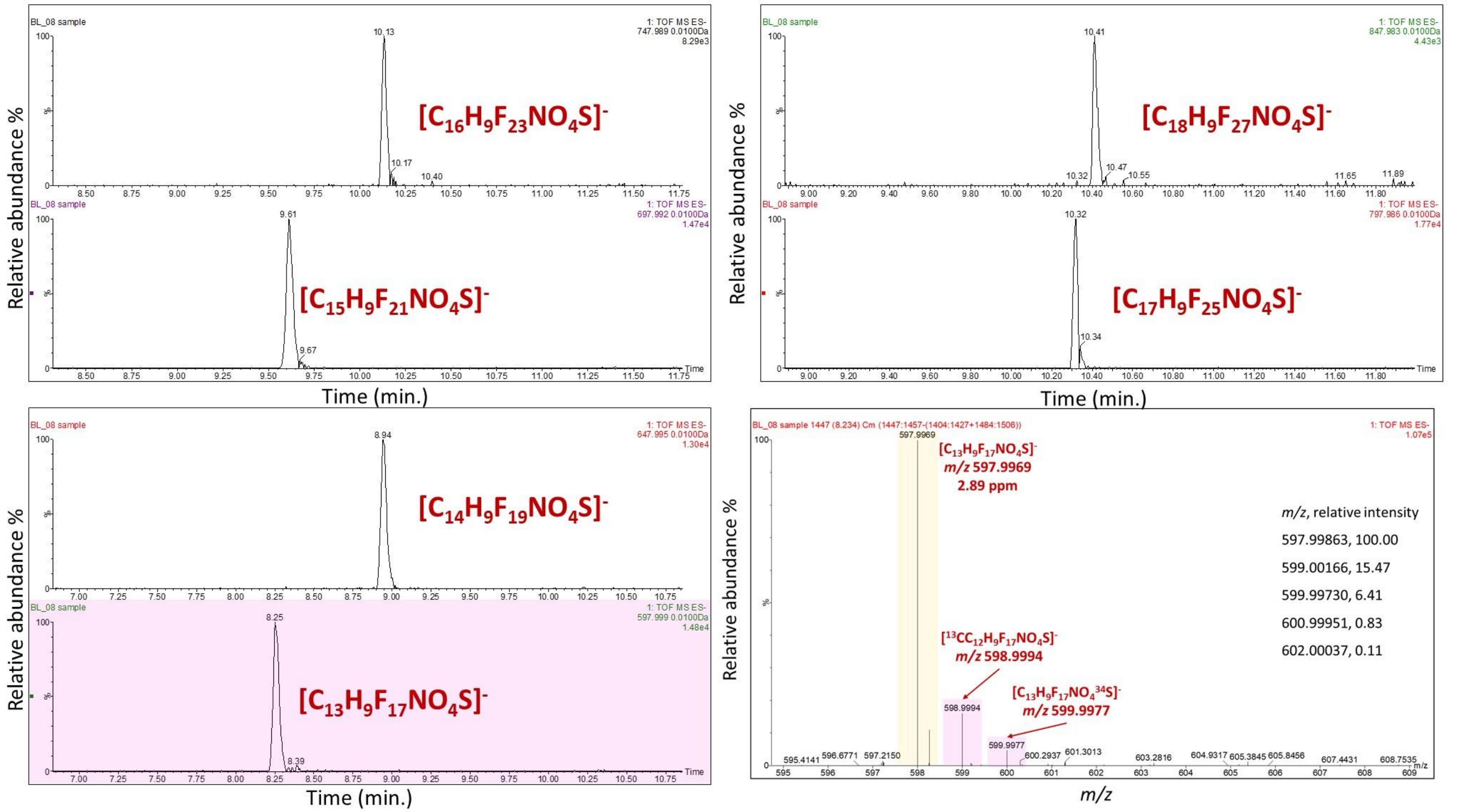

Table S21 – Tentatively identified bis(perfluoroalkylsulfonyl)imides and their chemical identifiers.

Class	Proposed structure	Acronym	$[M-H]^{-}$	*m/z*	Observed *m/z*	Mass accuracy (ppm)	Rt (min.)	CL
Bis(perfluoroalkylsulfonyl)imides	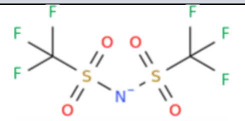	Bistriflimide	$[C_2F_6NO_4S_2]^-$	279.91784	279.9163	5.35	5.69	1a
	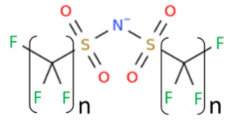	$[C_{10}HF_{22}NO_4S_2]$	$[C_{10}F_{22}NO_4S_2]^-$	679.89229	679.8928	0.75	9.45	4
		$[C_{11}HF_{24}NO_4S_2]$	$[C_{11}F_{24}NO_4S_2]^-$	729.88910	729.8885	0.82	9.96	4
		$[C_{12}HF_{26}NO_4S_2]$	$[C_{12}F_{26}NO_4S_2]^-$	779.88591	779.8859	0.01	10.21	4
		$[C_{13}HF_{28}NO_4S_2]$	$[C_{13}F_{28}NO_4S_2]^-$	829.88271	829.8802	3.02	10.32	4
		$[C_{14}HF_{30}NO_4S_2]$	$[C_{14}F_{30}NO_4S_2]^-$	879.87952	879.8777	2.07	10.40	4

Molecular formula	IUPAC	SMILES	CAS RN.	PubChem CID
$C_2HF_6NO_4S_2$	1,1,1-trifluoro-*N*-(trifluoromethylsulfonyl)methanesulfonamide	O=S(=O)(NS(=O)(=O)C(F)(F)F)C(F)(F)F	91742-21-1	157857
$C_{10}HF_{22}NO_4S_2$	1,1,2,2,3,3,4,4,5,5,5-undecafluoro-*N*-(1,1,2,2,3,3,4,4,5,5,5-undecafluoropentylsulfonyl)pentane-1-sulfonamide	O=S(=O)(NS(=O)(=O)C(F)(F)C(F)(F)C(F)(F)C(F)(F)C(F)(F)F)C(F)(F)C(F)(F)C(F)(F)C(F)(F)C(F)(F)F	752232-72-7	88203921
$C_{11}HF_{24}NO_4S_2$	1,1,2,2,3,3,4,4,5,5,5-undecafluoro-N-(1,1,2,2,3,3,4,4,5,5,6,6,6-tridecafluorohexanesulfonyl)pentane-1-sulfonamide	O=S(=O)(NS(=O)(=O)C(F)(F)C(F)(F)C(F)(F)C(F)(F)C(F)(F)C(F)(F)F)C(F)(F)C(F)(F)C(F)(F)C(F)(F)C(F)(F)F	-	-
$C_{12}HF_{26}NO_4S_2$	1,1,2,2,3,3,4,4,5,5,6,6,6-tridecafluoro-N-(1,1,2,2,3,3,4,4,5,5,6,6,6-tridecafluorohexylsulfonyl)hexane-1-sulfonamide	O=S(=O)(NS(=O)(=O)C(F)(F)C(F)(F)C(F)(F)C(F)(F)C(F)(F)C(F)(F)F)C(F)(F)C(F)(F)C(F)(F)C(F)(F)C(F)(F)C(F)(F)F	213342-85-9	10898051
$C_{13}HF_{28}NO_4S_2$	1,1,2,2,3,3,4,4,5,5,6,6,6-tridecafluoro-N-(1,1,2,2,3,3,4,4,5,5,6,6,7,7,7-pentadecafluoroheptanesulfonyl)hexane-1-sulfonamide	O=S(=O)(NS(=O)(=O)C(F)(F)C(F)(F)C(F)(F)C(F)(F)C(F)(F)C(F)(F)C(F)(F)F)C(F)(F)C(F)(F)C(F)(F)C(F)(F)C(F)(F)C(F)(F)F	-	-
$C_{14}HF_{30}NO_4S_2$	1,1,2,2,3,3,4,4,5,5,6,6,7,7,7-pentadecafluoro-N-(1,1,2,2,3,3,4,4,5,5,6,6,7,7,7-pentadecafluoroheptylsulfonyl)heptane-1-sulfonamide	O=S(=O)(NS(=O)(=O)C(F)(F)C(F)(F)C(F)(F)C(F)(F)C(F)(F)C(F)(F)C(F)(F)F)C(F)(F)C(F)(F)C(F)(F)C(F)(F)C(F)(F)C(F)(F)C(F)(F)F	1040629-78-4	154704528

Figure S16a – Chromatograms of six homologs classified as bis-FASI in this study. One homolog, Bistriflimide, includes the chromatogram of the analytical standard, shown in green. For the highlighted homolog (pink color), the precursor ion mass spectrum displays the molecular ion (yellow color) and its isotopologues, alongside simulated isotopologues and their corresponding intensities.

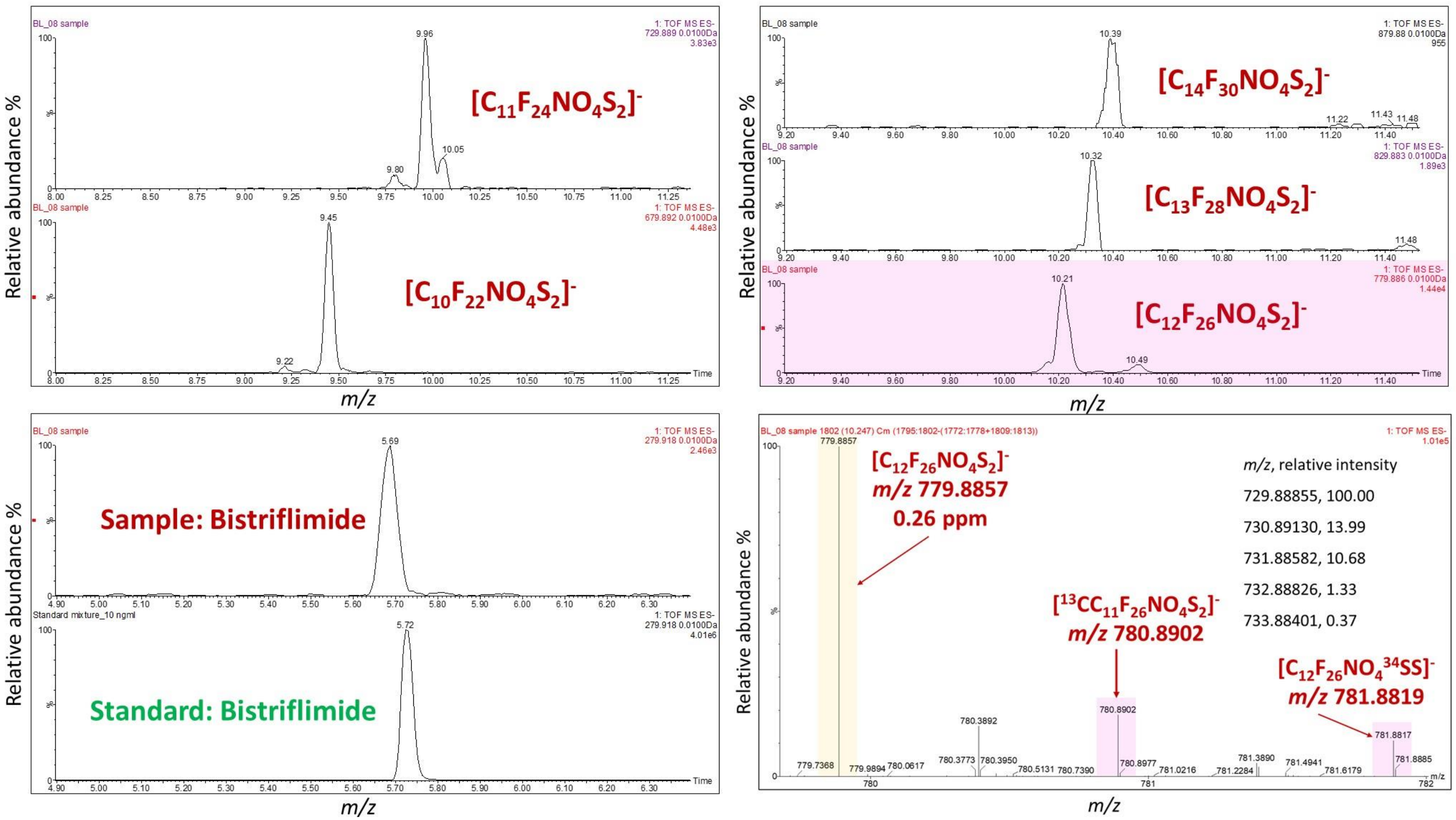

Figure S16b – Fragment ion spectra of Bistriflimide measured in the bream liver sample compared to the analytical standard. The mass spectrum shows the common fragmentation pattern, which is highlighted in the zoomed-in mass spectra (blue, yellow, and green for the molecular ion).

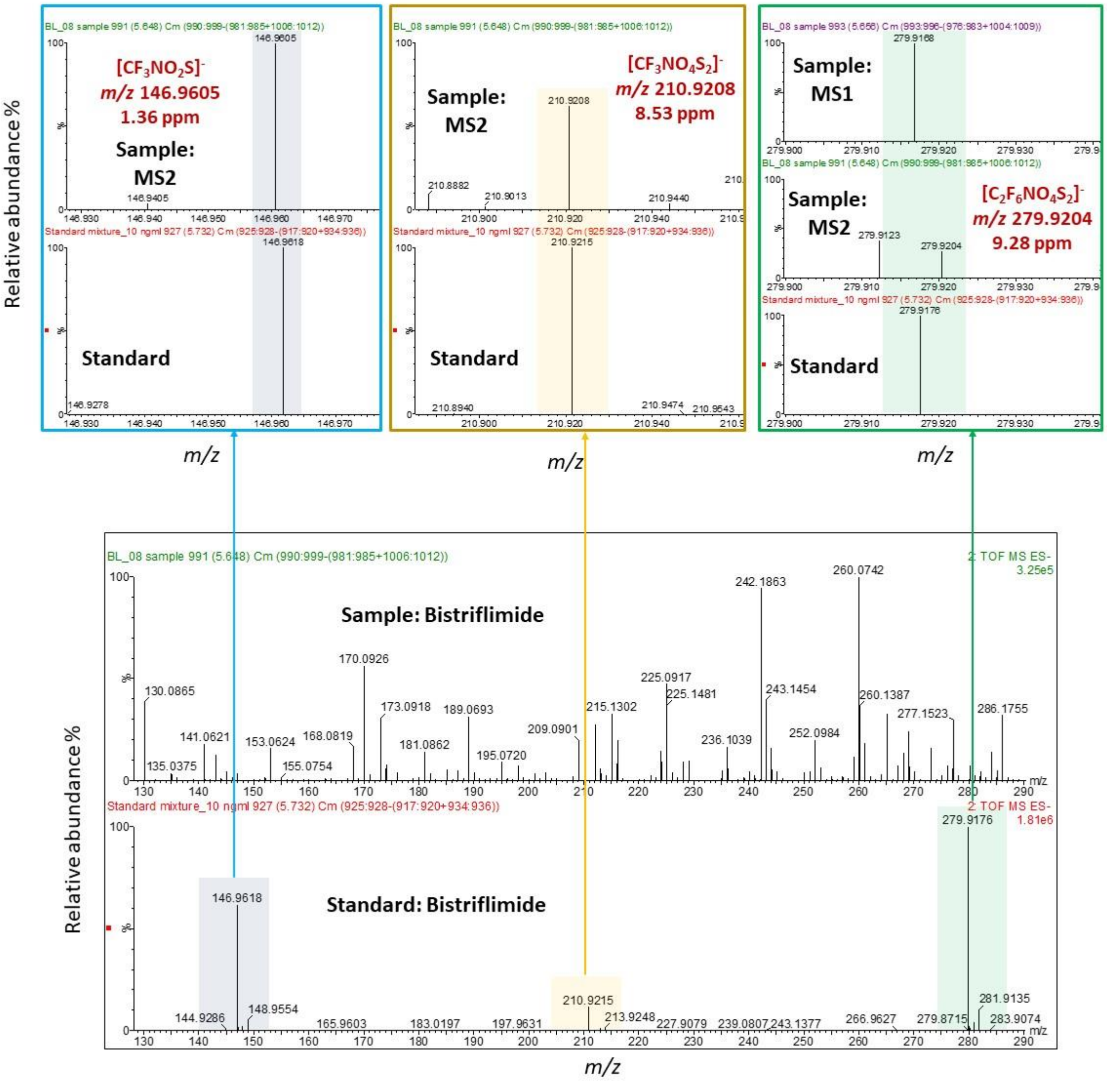

Figure S16c – Fragment ion mass spectrum of one of the homologs from the bis-FASI group (*m/z* 779.8857) along with the aligned peaks shown in the chromatograms.

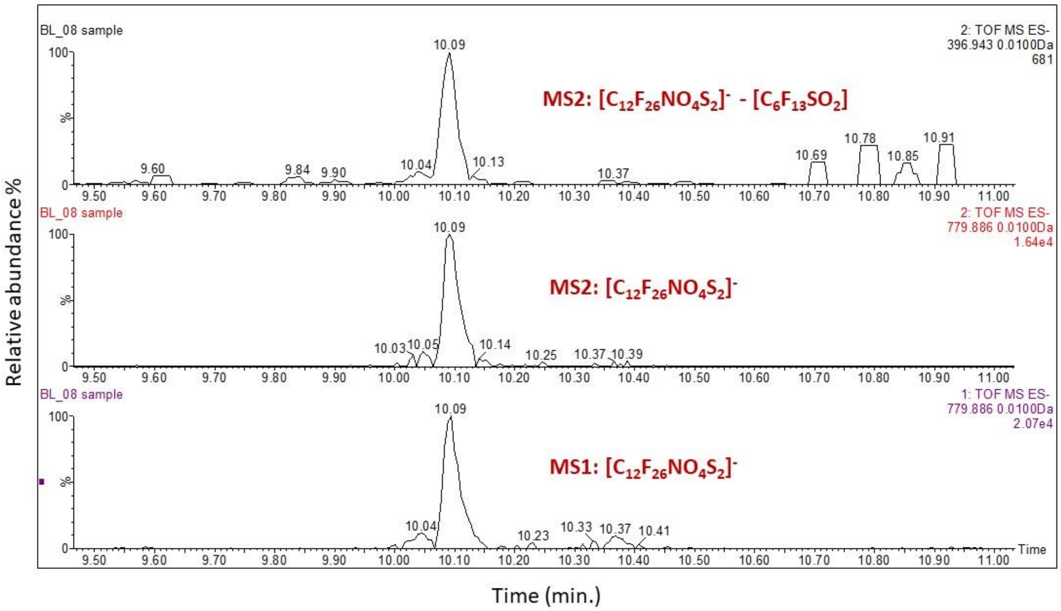

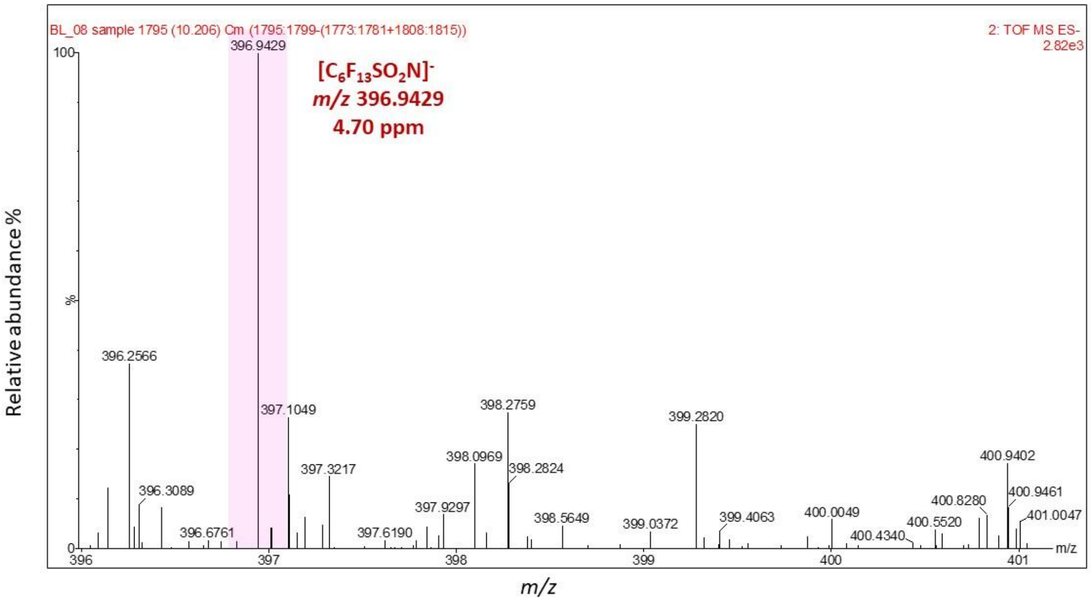

Figure S16d – Fragment ion mass spectrum and chromatogram of co-eluting compounds, highlighted in different colors. The chromatogram shows co-eluting peaks, confirming that the signals are not fragments of the same molecule but represent three distinct molecules eluting shortly one after the other.

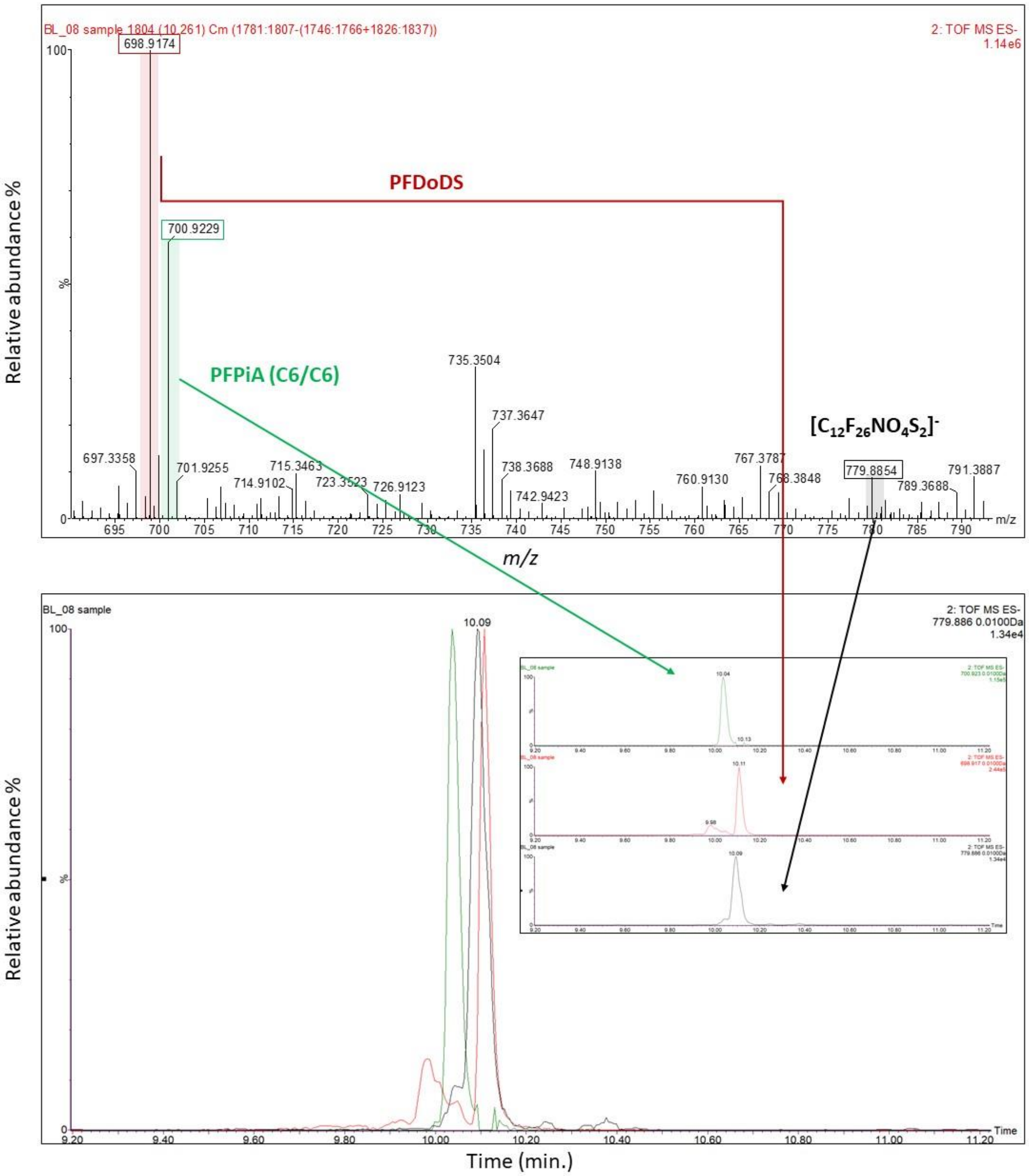

Table S22 – Tentatively identified PFOS derivatives and their chemical identifiers.

Class	Proposed structure	Acronym	$[M-H]^-$	*m/z*	Observed *m/z*	Mass accuracy (ppm)	Rt (min.)	CL
PFOS derivatives	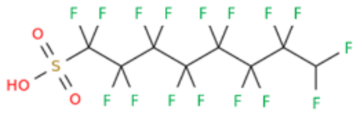	H-PFOS	$[C_8HF_{16}O_3S]^-$	480.93964	480.9383	2.79	6.90-7.58	4
	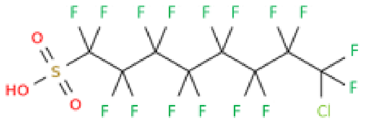	Cl-PFOS	$[C_8F_{16}ClO_3S]^-$	514.90067	514.9011	0.84	7.86, 8.03, 8.13	1a
	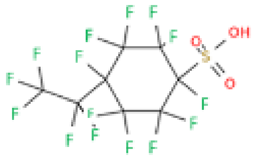	PFECHS	$[C_8F_{15}O_3S]^-$	460.93341	460.9317	3.71	7.30	1b

Molecular formula	IUPAC	SMILES	CAS RN	PubChem CID
$C_8H_2F_{16}O_3S$	1,1,2,2,3,3,4,4,5,5,6,6,7,8,8,8-hexadecafluorooctane-1-sulfonic acid	C(C(C(C(C(C(C(F)(F)S(=O)(=O)O)(F)F)(F)F)(F)F)(F)F)(F)F)(C(F)(F)F)F	2089109-58-8	139596859
$C_8HF_{16}ClO_3S$	8-chloro-1,1,2,2,3,3,4,4,5,5,6,6,7,7,8,8-hexadecafluorooctane-1-sulfonic acid	C(C(C(C(C(F)(F)Cl)(F)F)(F)F)(F)F)(C(C(C(F)(F)S(=O)(=O)O)(F)F)(F)F)(F)F	777011-38-8	15564880
$C_8HF_{15}O_3S$	1,2,2,3,3,4,5,5,6,6-decafluoro-4-(1,1,2,2,2-pentafluoroethyl)cyclohexane-1-sulfonic acid	C1(C(C(C(C(C1(F)F)(F)F)(F)(F)S(=O)(=O)O)(F)F)(F)F)(C(C(F)(F)F)(F)F)F	646-83-3	101650

Figure S17a – Chromatogram (upper) and fragment ion mass spectrum of H-PFOS. The chromatogram displays multiple eluting peaks between 6.90 and 7.58 minutes, corresponding to the positional isomers of H-PFOS. The fragment ion mass spectrum shows the molecular formula along with the corresponding isotopologues and their measured masses (red font). Additionally, the spectrum shows the predicted *m/z* and intensities of the five most intense isotopologues associated with the molecular formula of H-PFOS.

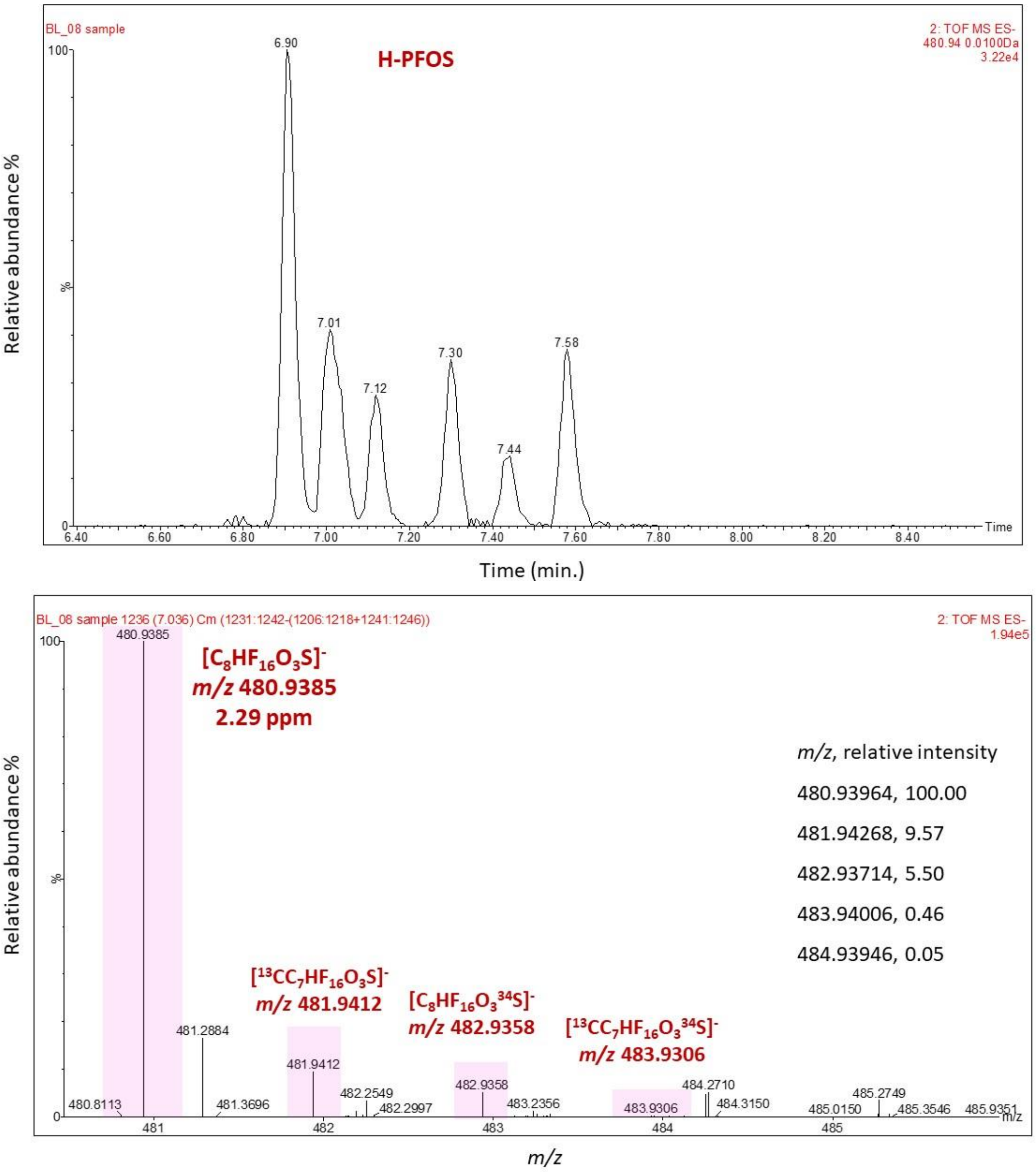

Figure S17b – The MS1 chromatogram of H-PFOS shows multiple peaks eluting at different retention times. The upper precursor ion mass spectrum is zoomed in to display the mass of H-PFOS, with different colors representing its various isomers as shown in the chromatogram.

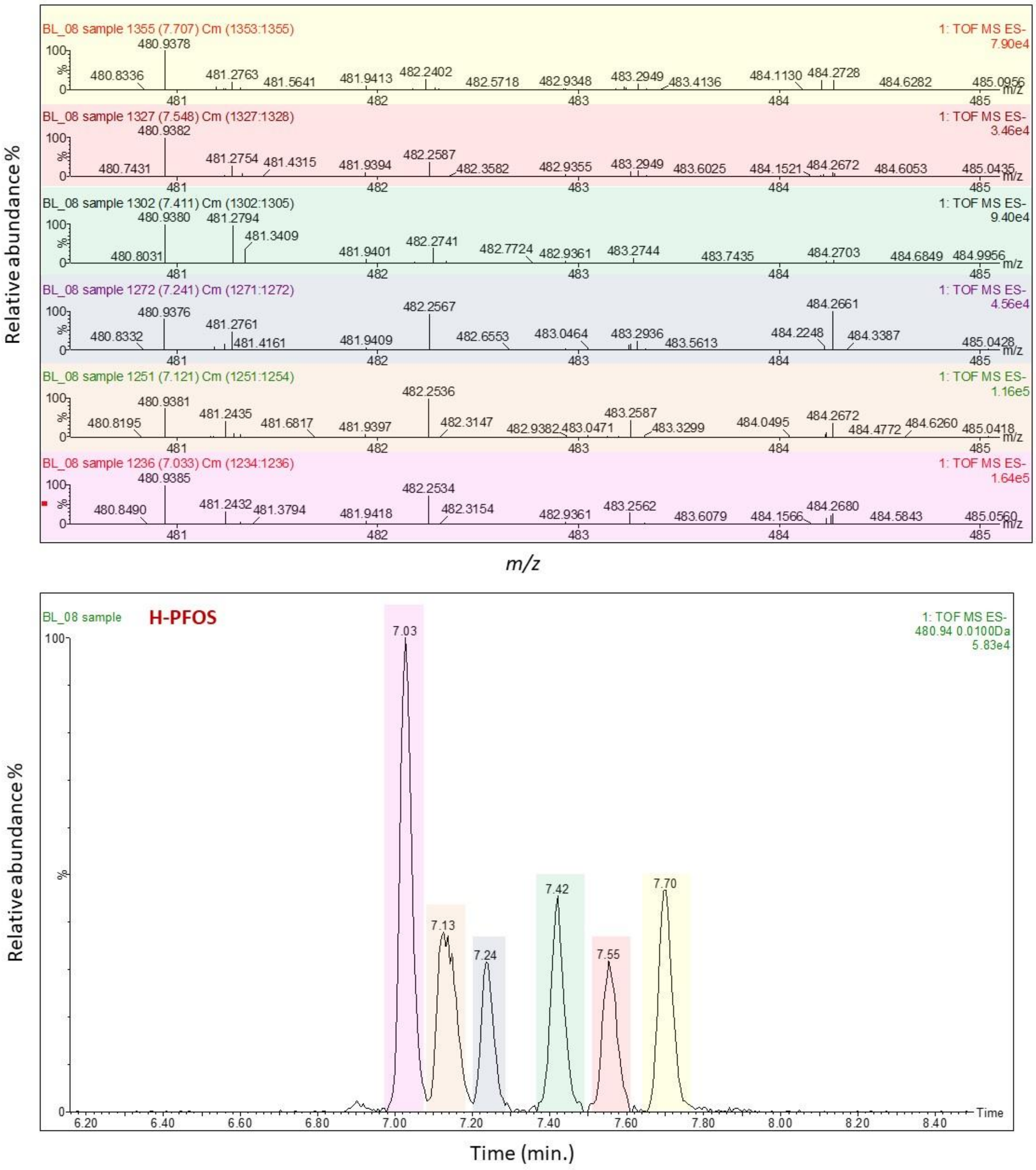

Figure S17c – Chromatogram and fragment ion mass spectrum of Cl-PFOS. The chromatogram displays the analytical standard of Cl-PFOS eluting at 8.21 min, alongside three peaks eluting at 7.86, 8.03, and 8.13 min, corresponding to positional isomers of Cl-PFOS in the sample. No fragments were observed in the fragment ion mass spectra of the bream liver sample. The mass spectrum shows the molecular ion and its isotopologues, confirming the presence of chlorine (Cl) within the molecular formula.

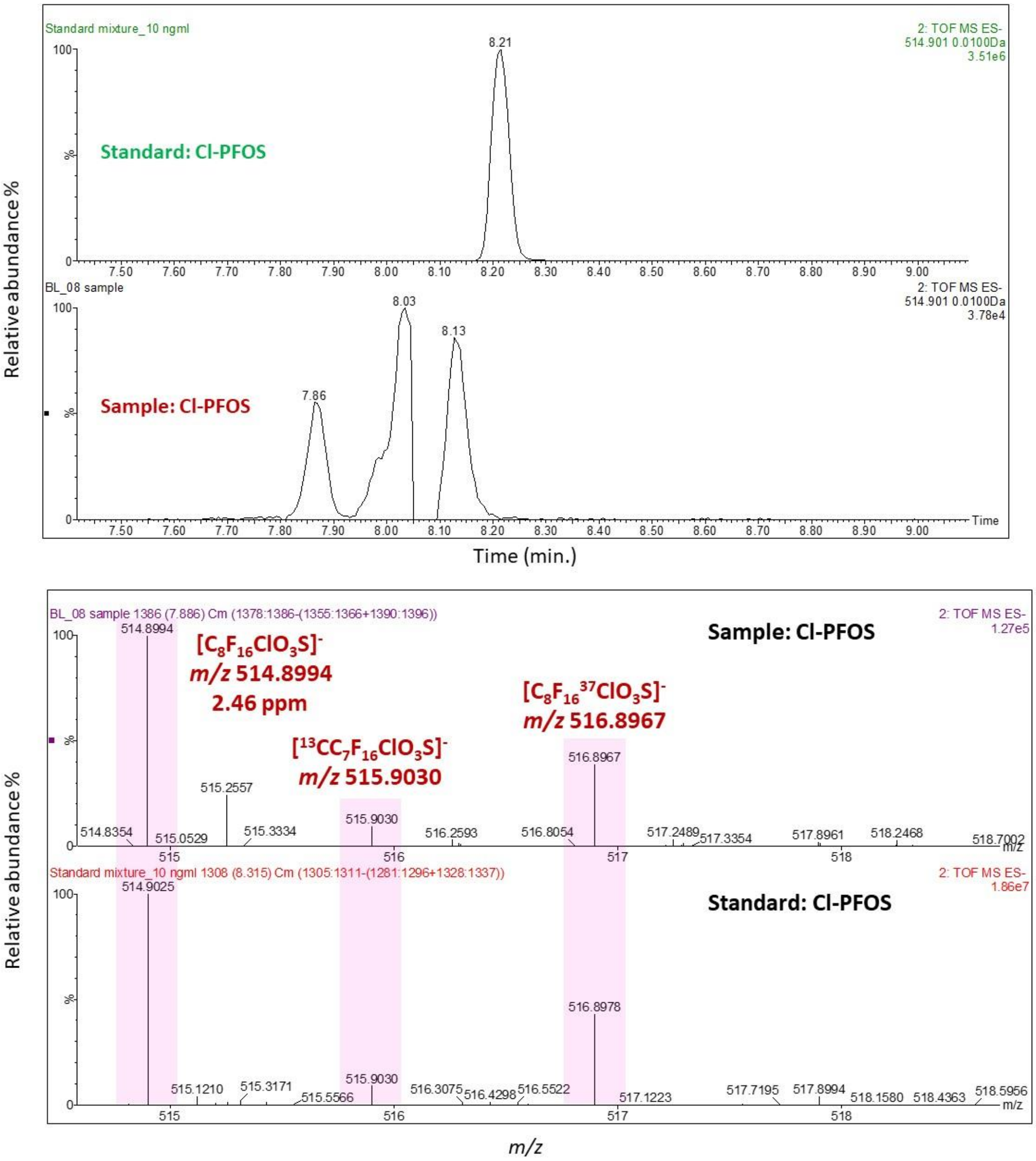

Figure S17d – Chromatogram and precursor ion mass spectrum of Cl-PFOS. The chromatogram displays three different isomers highlighted in different colors that correspond to the precursor ion mass spectra shown in the upper part of the figure.

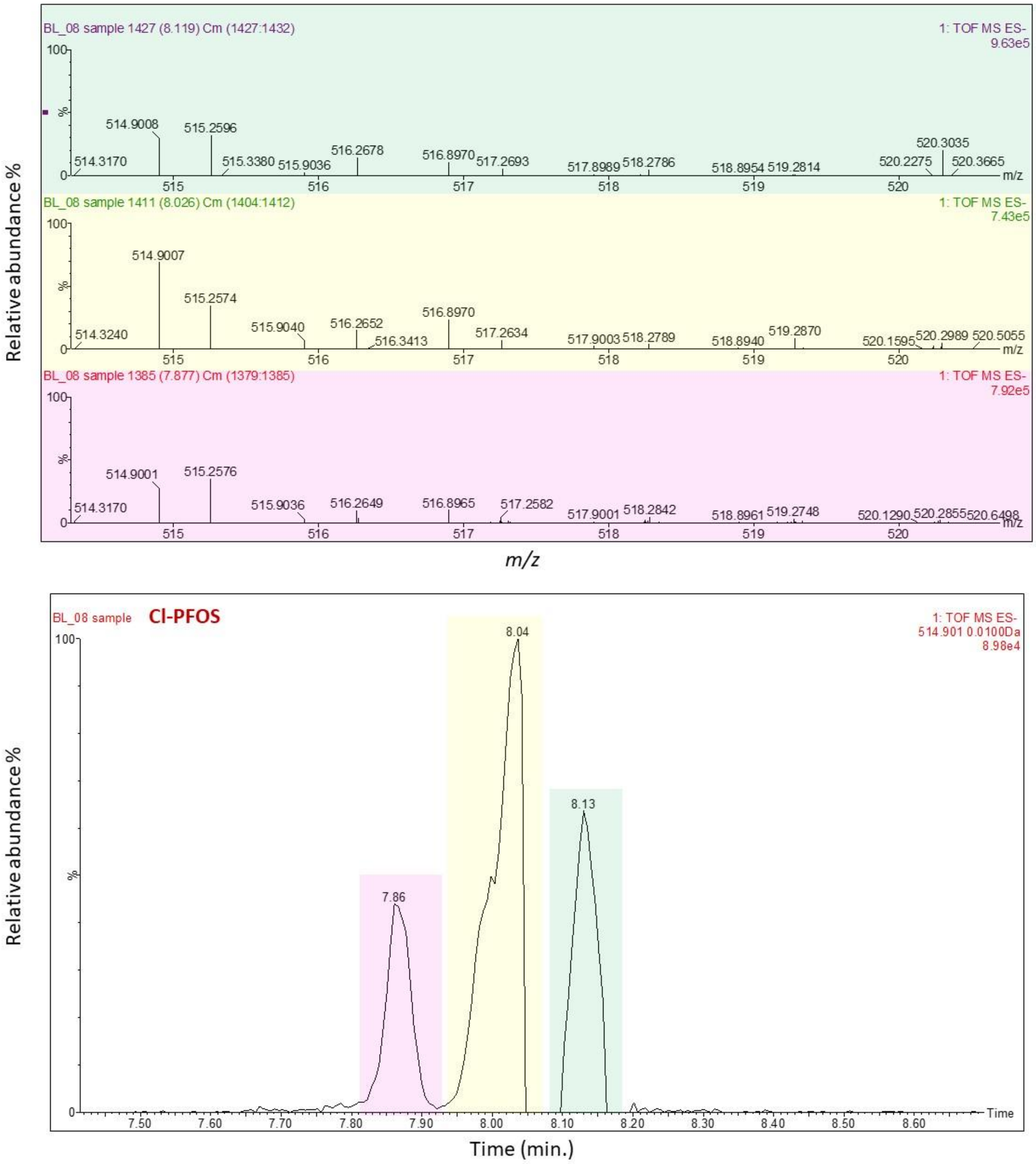

Figure S17e – Fragment ion mass spectrum of PFECHS in both the sample and the standard. The molecular ion is highlighted in yellow, while the common fragment observed in both the standard and the sample is shown in blue. The colored mass spectra provide a zoomed-in view, emphasizing the observed masses and confirming the structural consistency between the sample and the standard.

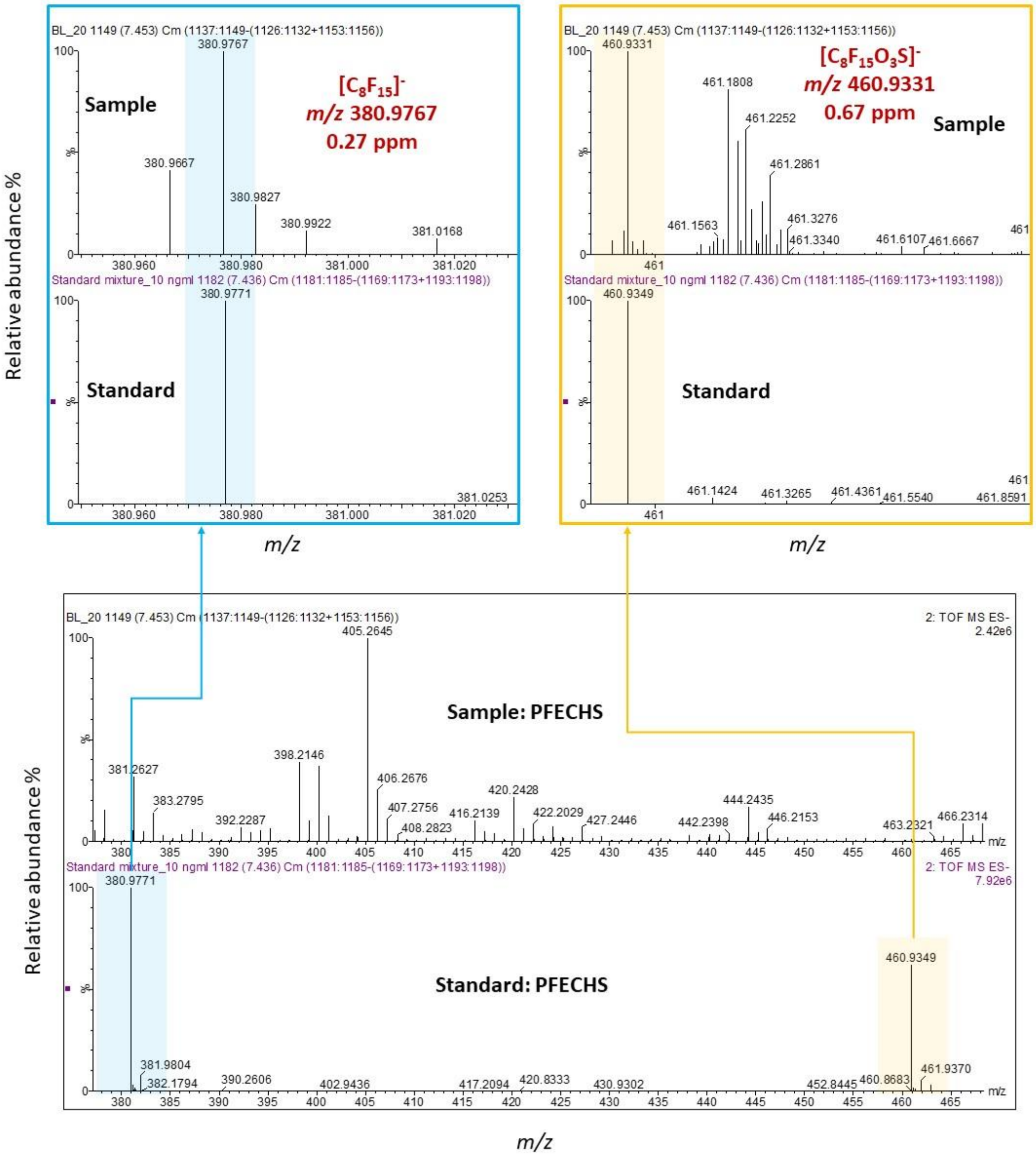

Table S23 – Tentatively identified perfluorinated compounds and their chemical identifiers.

**Proposed structure**	**Acronym**	**$[M\text{-}H]^-$**	***m/z***	**Observed *m/z***	**Mass accuracy (ppm)**	**Rt (min.)**	**CL**
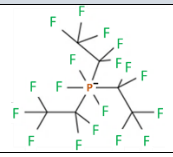	FAP	$[C_6F_{18}P]^-$	444.94557	444.9443	2.85	7.77	1a
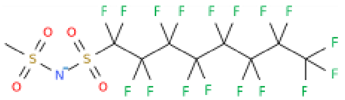	HMSO	$[C_9H_3F_{17}NO_4S_2]^-$	575.92375	575.9231	1.13	8.03	4
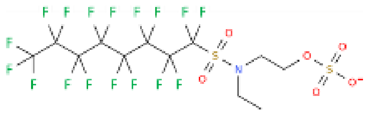	EtFOSA-Sulfate	$[C_{12}H_9F_{17}NO_6S_2]^-$	649.96053	649.9602	0.51	9.18	4

**Molecular formula**	**IUPAC**	**SMILES**	**CAS RN.**	**PubChem CID**
$C_{12}H_{11}F_{18}N_2P$	1-Ethyl-3-methylimidazolium tris(pentrafluoroethyl)trifluorophosphate	CCN1C=C[N+](=C1)C.C(C(F)(F)F)([P-](C(C(F)(F)F)(F)F)(C(C(F)(F)F)(F)F)(F)(F)F)(F)F	377739-43-0	164679371
$C_9H_4F_{17}NO_4S_2$	1,1,2,2,3,3,4,4,5,5,6,6,7,7,8,8,8-heptadecafluoro-*N*-methylsulfonyloctane-1-sulfonamide	CS(=O)(=O)NS(=O)(=O)C(C(C(C(C(C(C(C(F)(F)F)(F)F)(F)F)(F)F)(F)F)(F)F)(F)F)(F)F	-	140529081
$C_{12}H_{10}F_{17}NO_6S_2$	2-[ethyl(1,1,2,2,3,3,4,4,5,5,6,6,7,7,8,8,8-heptadecafluorooctylsulfonyl)amino]ethyl hydrogen sulfate	CCN(CCOS(=O)(=O)O)S(=O)(=O)C(C(C(C(C(C(C(C(F)(F)F)(F)F)(F)F)(F)F)(F)F)(F)F)(F)F)(F)F	2558-75-0	17509

Figure S18a – Fragment ion mass spectrum showcasing FAP detected in the bream liver sample, compared to the analytical standard. The mass spectrum highlights the molecular ion (yellow) and the common fragmentation pattern observed in both the sample and the analytical standard.

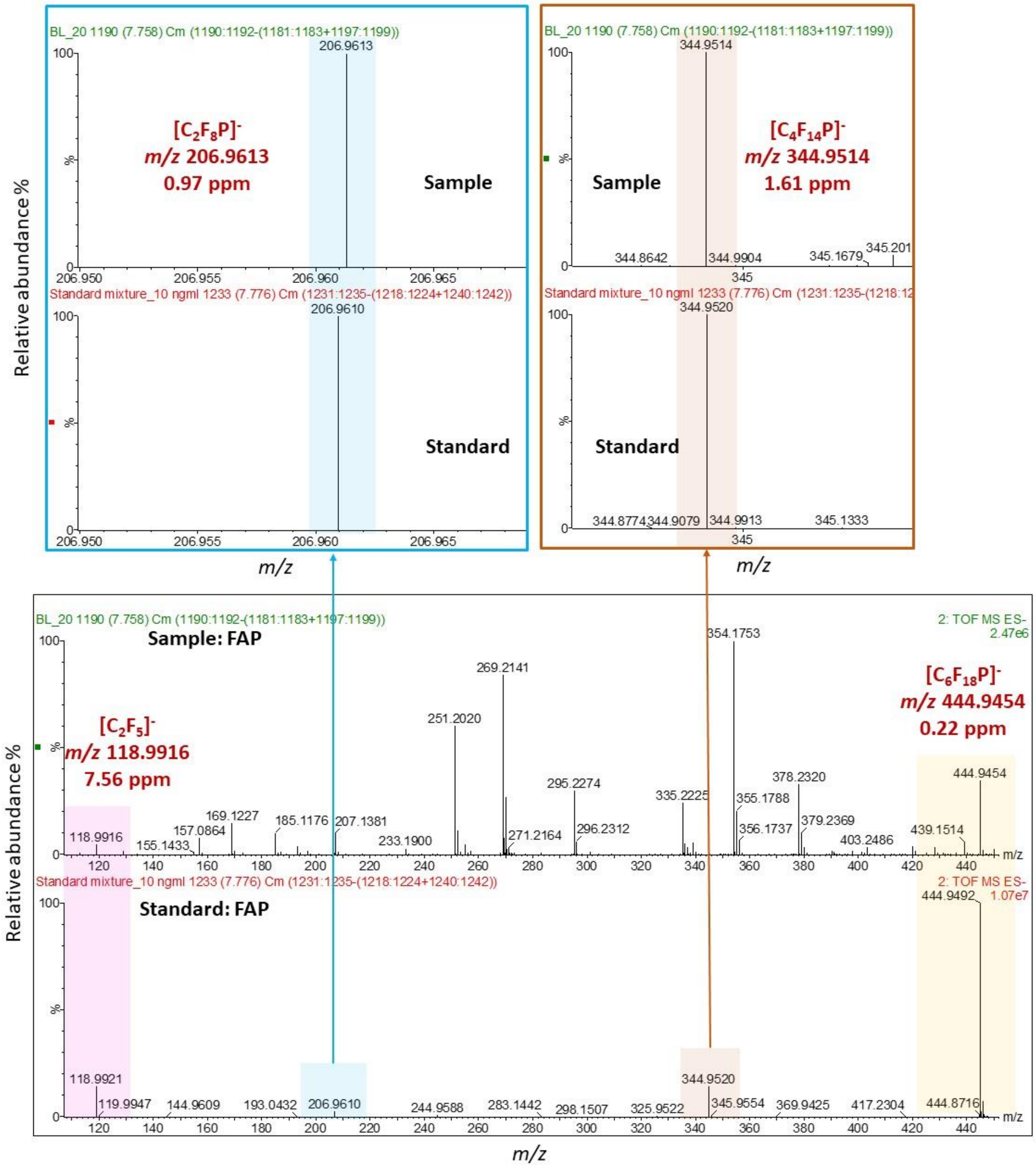

Figure S18b – Fragment ion mass spectrum of *m/z* 575.9238 detected in the bream liver sample. The bottom spectrum displays the molecular ion along with its isotopologues, and the predicted isotopologues for the proposed molecular formula. The upper spectrum highlights two observed fragments supporting the proposed molecular structure.

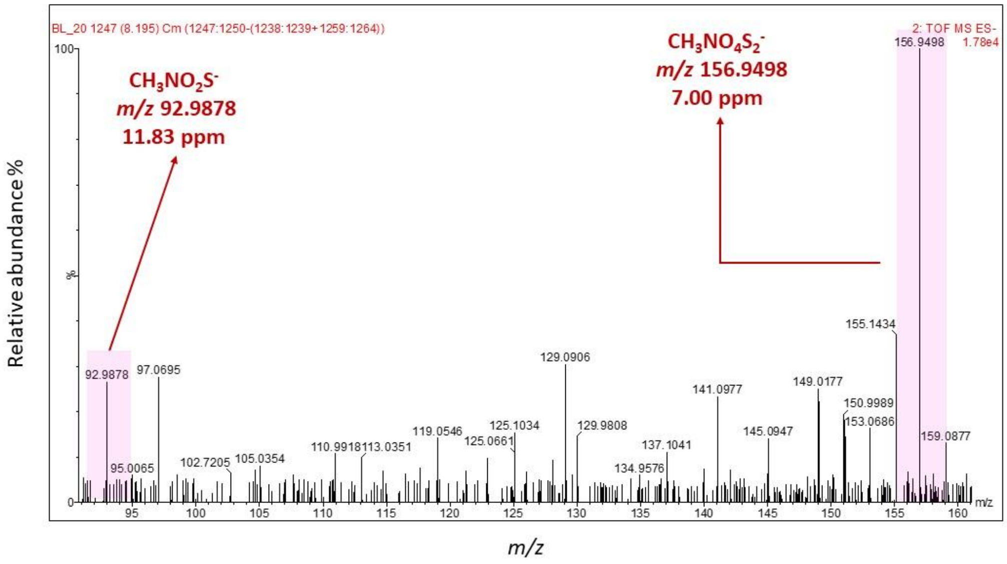

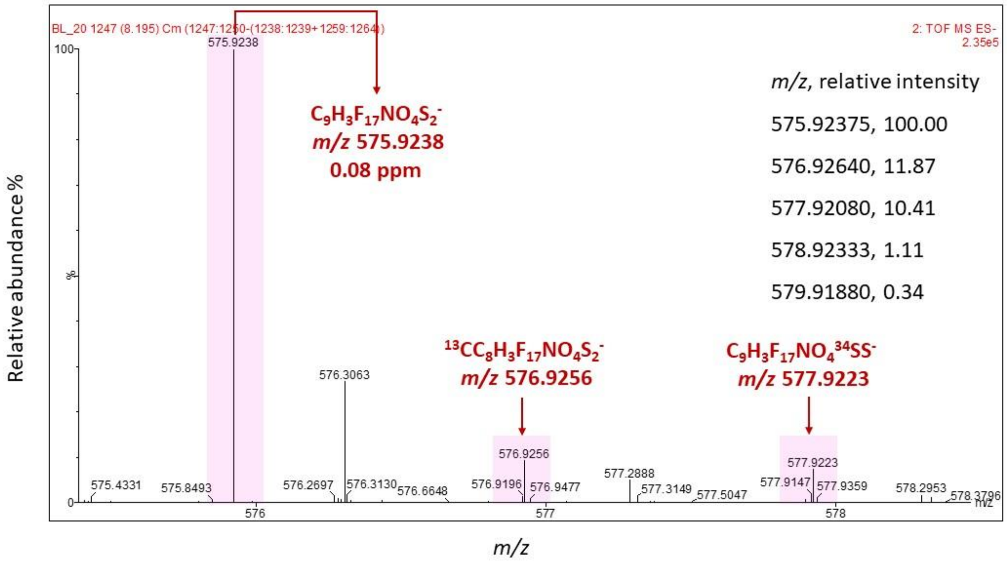

Figure S18c – Fragment ion mass spectrum for *m/z* 649.9607 measured in the bream liver sample. The spectrum shows both measured and predicted isotopologues. The blue-highlighted portion of the structure corresponds to the fragment detected in fragment ion mass spectrum.

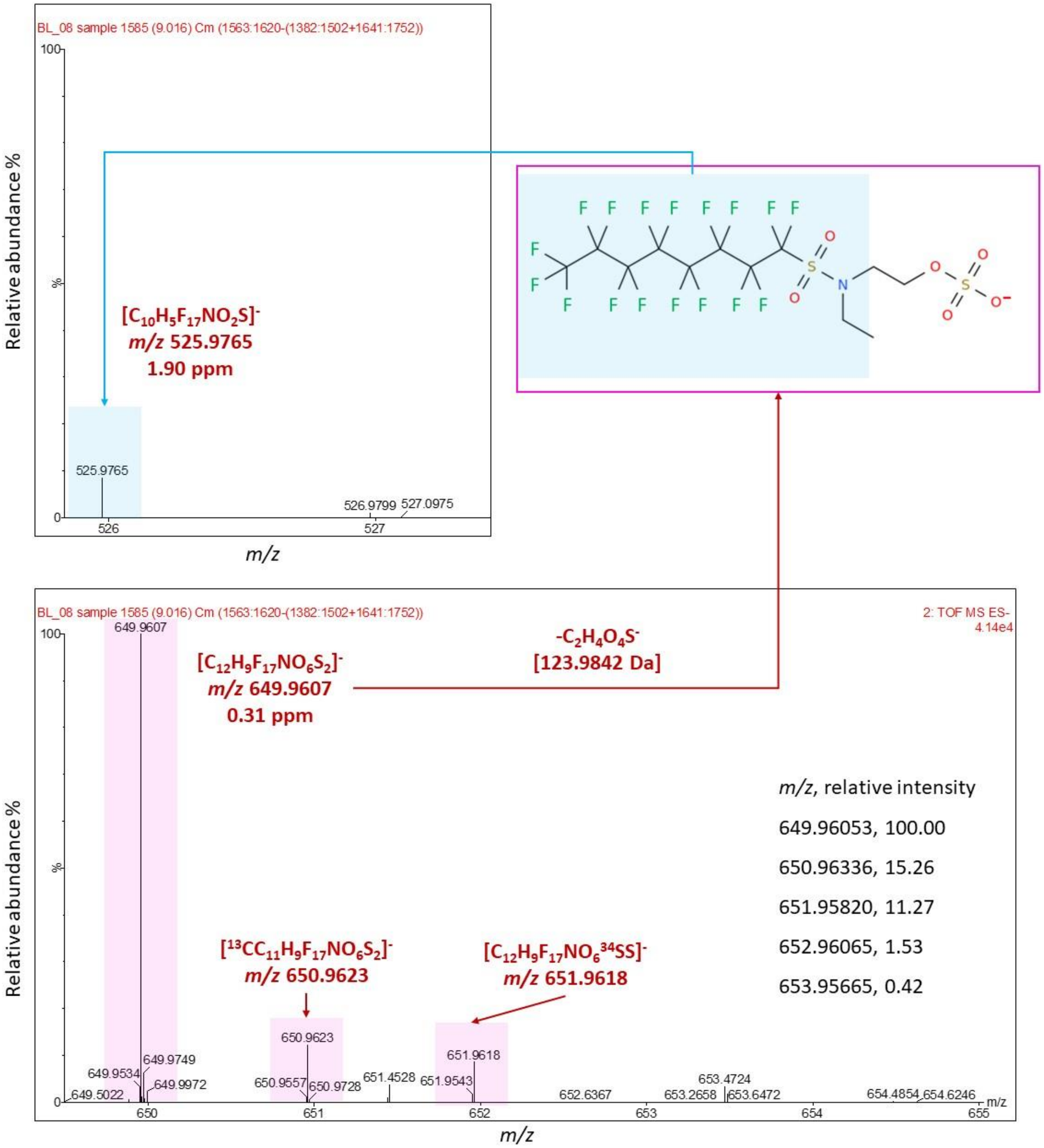

Figure S19 – Visualization of co-eluting signals in the mass spectrum, with co-eluted signals highlighted in pink. The chromatogram displays overlaid mass spectra for three homologs from the PFSA and PFESA series, demonstrating different elution times for lower and higher homologs within the same series.

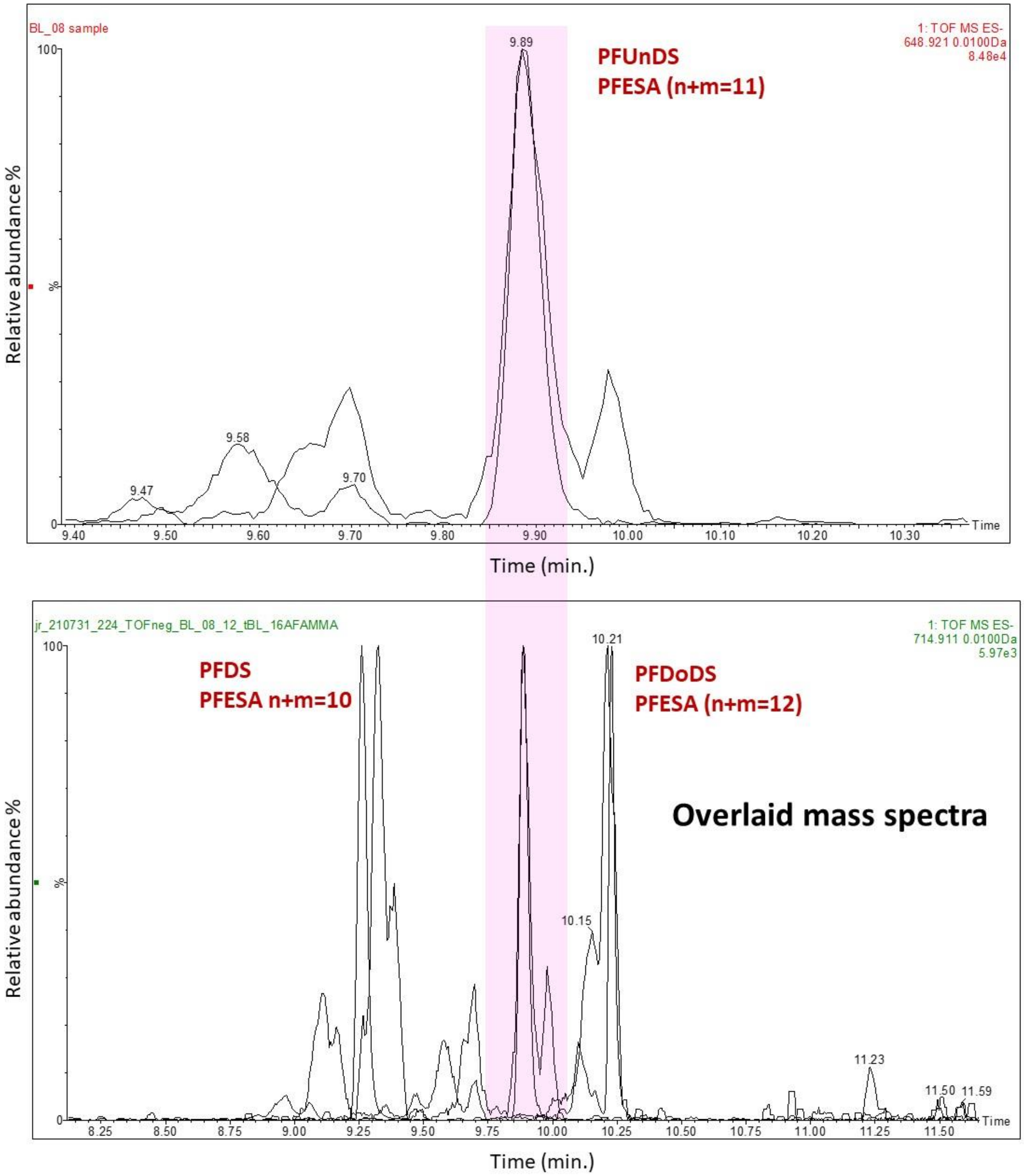

Figure S20 – Chromatograms demonstrating the individuality of co-eluting compounds, where fragment ions perfectly overlay with their corresponding parent ions, confirming their distinct identity and alignment in retention time.

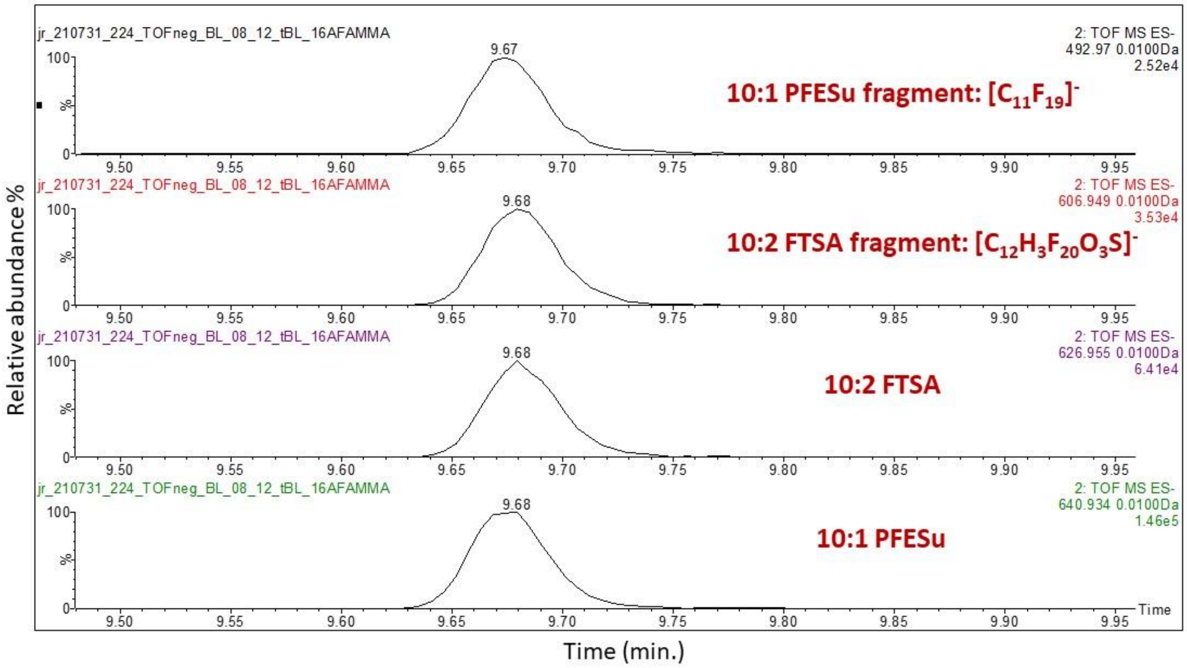

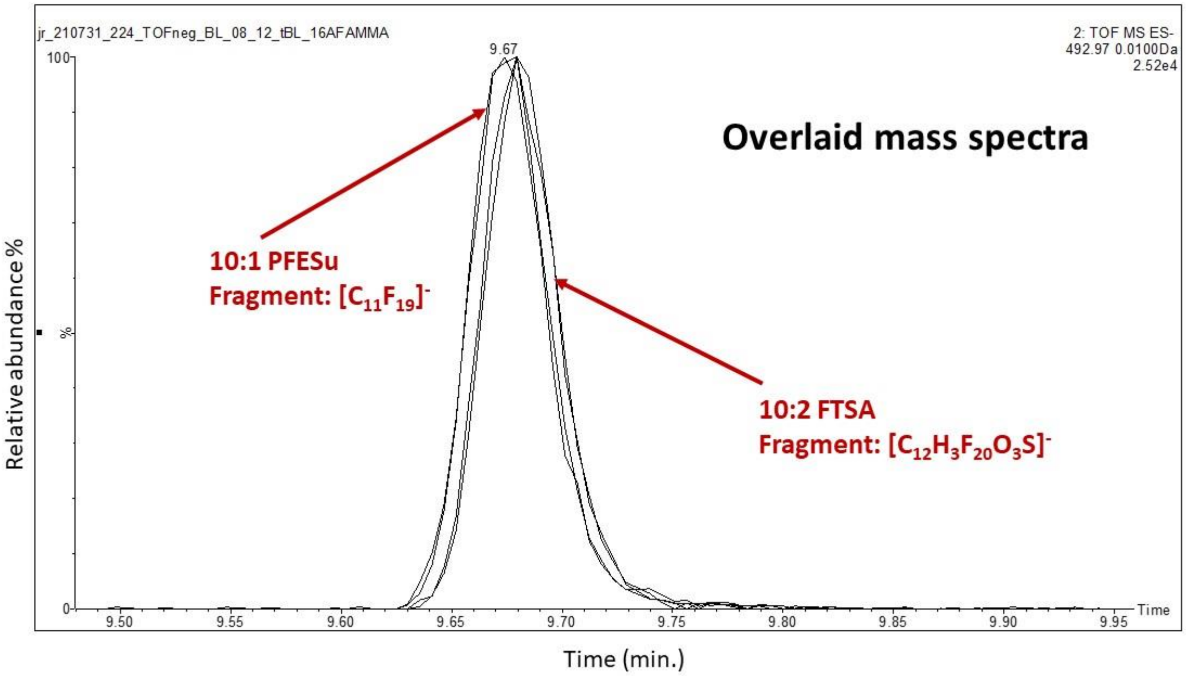

References:

(1) Katajamaa, M.; Miettinen, J.; Oresic, M. MZmine: Toolbox for Processing and Visualization of Mass Spectrometry Based Molecular Profile Data. *Bioinformatics* **2006**, *22* (5), 634–636. https://doi.org/10.1093/bioinformatics/btk039.

(2) Schmid, R.; Heuckeroth, S.; Korf, A.; Smirnov, A.; Myers, O.; Dyrlund, T. S.; Bushuiev, R.; Murray, K. J.; Hoffmann, N.; Lu, M.; Sarvepalli, A.; Zhang, Z.; Fleischauer, M.; Dührkop, K.; Wesner, M.; Hoogstra, S. J.; Rudt, E.; Mokshyna, O.; Brungs, C.; Ponomarov, K.; Mutabdžija, L.; Damiani, T.; Pudney, C. J.; Earll, M.; Helmer, P. O.; Fallon, T. R.; Schulze, T.; Rivas-Ubach, A.; Bilbao, A.; Richter, H.; Nothias, L.-F.; Wang, M.; Orešič, M.; Weng, J.-K.; Böcker, S.; Jeibmann, A.; Hayen, H.; Karst, U.; Dorrestein, P. C.; Petras, D.; Du, X.; Pluskal, T. Integrative Analysis of Multimodal Mass Spectrometry Data in MZmine 3. *Nat Biotechnol* **2023**, *41* (4), 447–449. https://doi.org/10.1038/s41587-023-01690-2.

(3) *KNIME Analytics Platform | KNIME*. https://www.knime.com/knime-analytics-platform (accessed 2024-05-03).

(4) *CompTox Chemicals Dashboard*. https://comptox.epa.gov/dashboard/chemical-lists/pfasmaster (accessed 2023-01-25).

(5) Patiny, L.; Borel, A. ChemCalc: A Building Block for Tomorrow's Chemical Infrastructure. *J. Chem. Inf. Model.* **2013**, *53* (5), 1223–1228. https://doi.org/10.1021/ci300563h
